# Accuracy of symptom checker for the diagnosis of sexually transmitted infections using machine learning and Bayesian network algorithms

**DOI:** 10.1186/s12879-024-10285-4

**Published:** 2024-12-18

**Authors:** Nyi Nyi Soe, Janet M Towns, Phyu Mon Latt, Owen Woodberry, Mark Chung, David Lee, Jason J Ong, Eric P.F. Chow, Lei Zhang, Christopher K. Fairley

**Affiliations:** 1 Melbourne Sexual Health Centre, Alfred Health, 580 Swanston Street, Carlton, Melbourne, VIC 3053 Australia; 2 School of Translational Medicine, Faculty of Medicine, Nursing and Health Sciences, Monash University, Melbourne, Australia; 3 Faculty of Information Technology, Monash Data Futures Institute, Monash University, Melbourne, Australia; 4 China-Australia Joint Research Centre for Infectious Diseases, School of Public Health, Xi’an Jiaotong University Health Science Center, Xi’an, Shaanxi, 710061 China; 5 Centre for Epidemiology and Biostatistics, Melbourne School of Population and Global Health, The University of Melbourne, Melbourne, Australia

**Keywords:** Sexually transmitted infections, Machine learning, Bayesian networks, Symptom Checker

## Abstract

**Background:**

A significant proportion of individuals with symptoms of sexually transmitted infection (STI) delay or avoid seeking healthcare, and digital diagnostic tools may prompt them to seek healthcare earlier. Unfortunately, none of the currently available tools fully mimic clinical assessment or cover a wide range of STIs.

**Methods:**

We prospectively invited attendees presenting with STI-related symptoms at Melbourne Sexual Health Centre to answer gender-specific questionnaires covering the symptoms of 12 common STIs using a computer-assisted self-interviewing system between 2015 and 2018. Then, we developed an online symptom checker (iSpySTI.org) using Bayesian networks. In this study, various machine learning algorithms were trained and evaluated for their ability to predict these STI and anogenital conditions. We used the Z-test to compare their average area under the ROC curve (AUC) scores with the Bayesian networks for diagnostic accuracy.

**Results:**

The study population included 6,162 men (median age 30, IQR: 26–38; approximately 40% of whom had sex with men in the past 12 months) and 4,358 women (median age 27, IQR: 24–31). Non-gonococcal urethritis (NGU) (23.6%, 1447/6121), genital warts (11.7%, 718/6121) and balanitis (8.9%, 546/6121) were the most common conditions in men. Candidiasis (16.6%, 722/4538) and bacterial vaginosis (16.2%, 707/4538) were the most common conditions in women. During evaluation with unseen datasets, machine learning models performed well for most male conditions, with the AUC ranging from 0.81 to 0.95, except for urinary tract infections (UTI) (AUC 0.72). Similarly, the models achieved AUCs ranging from 0.75 to 0.95 for female conditions, except for cervicitis (AUC 0.58). Urethral discharge and other urinary symptoms were important features for predicting urethral gonorrhoea, NGU and UTIs. Similarly, participants selected skin images that were similar to their own lesions, and the location of the anogenital skin lesions were also strong predictors. The vaginal discharge (odour, colour) and itchiness were important predictors for bacterial vaginosis and candidiasis. The performance of the machine learning models was significantly better than Bayesian models for male balanitis, molluscum contagiosum and genital warts (*P* < 0.05) but was similar for the other conditions.

**Conclusions:**

Both machine learning and Bayesian models could predict correct diagnoses with reasonable accuracy using prospectively collected data for 12 STIs and other common anogenital conditions. Further work should expand the number of anogenital conditions and seek ways to improve the accuracy, potentially using patient collected images to supplement questionnaire data.

**Supplementary Information:**

The online version contains supplementary material available at 10.1186/s12879-024-10285-4.

## Introduction

Sexually transmitted infections (STIs) are increasing globally [1, 2], with syphilis, gonorrhoea, chlamydia and trichomoniasis causing over 1 million new infections daily [1]. The rising incidence of syphilis is a particular concern, because increasing maternal cases translate into congenital infections [3]. Unfortunately, global efforts are failing to achieve the 2025 targets for reducing STIs. The World Health Organization (WHO) recommends improving primary prevention, diagnosis, and treatment, using innovative approaches such as telehealth and digital health services, and raising public awareness of STIs [1, 4]. 

The early recognition of symptoms of an STI that prompt timely health-seeking behaviour and early treatment is an important part of effective STI control, in addition to health benefits for individuals and their partners [5]. A significant proportion of individuals (28–82%) with STI symptoms who present for care have delayed seeking healthcare for more than 7 days from the onset of symptoms [5, 6]. Of greater concern are those who never present to health care. These delayed presentations arise from limited access to healthcare, lack of knowledge, poor symptom awareness, stigma and embarrassment [7–9]. The time between the onset of symptoms and healthcare seeking varied widely (3–60 days) across different STIs. Individuals with primary syphilis often seek care later than those with genital herpes [10]. This delay is particularly concerning as, for example, a chancre from primary syphilis will resolve without treatment, leading individuals to potentially forgo healthcare while still being able to transmit syphilis and unknowingly advancing to long-term complications.

Digital tools for STI diagnosis could potentially improve health-seeking behaviour among symptomatic individuals. One-third of adults used the internet to check health conditions, with most attempting self-diagnosis through symptom exploration [11, 12]. In Australia, 80% of people searched for health information online, and 40% sought online self-treatment advice [13]. STI-related searches are popular on Google and there was a positive correlation between gonorrhoea rates and the frequency of gonorrhoea-related Google searches across U.S. states [14]. However, existing STI diagnostic apps have significant limitations [15]. Many provide only health information without interactivity, assess only STI risk for asymptomatic individuals, or charge fees. A review by Gibbs et al. found that only 29% of STI apps met basic quality standards (modified Health On the Net (HON) scores), less than half covered multiple STIs, and over a quarter contained potentially harmful information [16]. Current tools lack gender-specific questionnaires and transparency in algorithm development. To address these limitations, the Melbourne Sexual Health Centre (MSHC) designed a gender-specific symptom checker based on prospectively collected questionnaire data of common STI symptoms and anogenital conditions. Data were collected over three years and developed “iSpySTI” [17], a web-based symptom checker using a Bayesian Network model (hereafter referred to as iSpySTI). The iSpySTI generated a probability of specific diagnoses based on users’ responses and referral letters for users to take to their health professionals. The iSpySTI achieved reasonable accuracy (80–95% sensitivity and 38–76% specificity) in identifying 12 STIs and anogenital conditions when testing with prospective data. We have not formally evaluated the impact of iSpySTI on STI care since its deployment. MSHC is currently planning to redesign the user interface to enhance the user experience based on feedback received [18]. 

Machine learning approaches have shown promise in HIV and STI public health interventions. Bao et al., [19] and Xu et al., [20–22] developed algorithms using demographic and sexual behaviour data to predict HIV and STI risk. Latt et al., [23] used machine learning-predicted scores and Gini index to examine HIV and STI distribution disparities. For symptomatic individuals, Soe et al., [24] achieved 84% accuracy in differentiating STIs from non-STI conditions by applying a machine learning model on manually extracted data from clinical text. Artificial Intelligence (AI)-based symptom checkers have shown potential to enhance health-seeking behaviour and reduce anxiety [25]. These AI algorithms, incorporating comprehensive data, improved the diagnosis accuracy compared to ordinary symptom checkers (59–74% vs. 40–54% accuracy) [13]. This study aimed to develop comprehensive machine learning models for checking STI symptoms and compare their performance with the current Bayesian models used for iSpySTI. Redesigning the iSpySTI with machine learning models in Python could enable integration with MSHC’s existing digital health tools, such as MySTIRisk and image recognition tools [26]. This would provide users with a comprehensive package of services.

## Methods

### Study design

Our study used prospectively collected data from a previous study of clients presenting with symptoms to MSHC which is the largest public sexual health clinic in Australia [15]. Our study involved four main steps: 1 developing classification models, 2 evaluating the models, 3 interpreting the predictions, and 4 conducting statistical analyses for each STI in both male and female sexual health clinic attendees. We followed the Transparent Reporting of a multivariable prediction model for Individual Prognosis Or Diagnosis (TRIPOD) guidelines [27]. 

### Data source

The data were collected from March 2015 to April 2018 for the “What is it?” project aimed at developing the iSpySTI [17] (Alfred Hospital Ethics Committee, 332/14). Before these data were collected, we developed the questions by retrospectively reviewing 200 cases each for twelve common conditions in the model to determine the common presenting symptoms for each specific condition. We then systematically designed questionnaires to cover these common symptoms [15] (Table S1). We included images of the conditions in the questionnaire and asked individuals to say if these images were ‘nothing like’, ‘a bit like’ or ‘exactly like’ their condition. Symptomatic individuals attending MSHC were invited to participate in a study and complete the questions using a Computer-Assisted Self-Interviewing (CASI) system. Given the different nature of presenting symptoms and images for these conditions in men and women, two separate questionnaires were designed. After completing the questionnaire, participants had a consultation with an experienced sexual health clinician who was not aware of their responses to these questions. A clinical diagnosis was made based on history, examination and the results of laboratory tests. For each consultation, clinicians entered the diagnoses from a pre-specified diagnostic list. The datasets were cleaned by removing the rows with missing values and outliers. The male dataset contained 6,121 unique participants, while the female dataset contained 4,358 participants.

### Predictors and outcomes

The male dataset comprised 44 variables, including demographics, sexual behaviours, anogenital skin lesions, urethral discharge symptoms, and urinary symptoms (Table S1). The female dataset comprised 45 variables, including demographics, sexual behaviours, anogenital skin lesions, vaginal discharge symptoms, pelvic pain symptoms, vaginal bleeding and dyspareunia symptoms, and urinary symptoms. The primary outcome was the confirmed diagnosis by the clinician. The diagnoses for male conditions were balanitis, urethral gonorrhoea, genital herpes, molluscum contagiosum, primary syphilis, non-gonococcal urethritis (NGU), urinary tract infection (UTI) and genital warts. The diagnoses for female conditions were bacterial vaginosis, candidiasis, cervicitis, cystitis, genital herpes, molluscum contagiosum, genital warts and pelvic inflammatory disease (PID).

### Bayesian networks model used to develop iSpySTI

We used Bayesian networks to develop the iSpySTI [17] in 2017–2018. Bayesian networks [28] are mathematical models used for reasoning under uncertainty and are widely used in medical decision support. In our Bayesian network models, nodes represent the variable of interest and edges show the relationship between these variables. We conducted data pre-processing, variable selection, discretization, and structure learning with causal minimum message length (CaMML) software [29]. Parameterization was done using expectation-maximization algorithms. We validated the models using 5-fold cross-validation. The models performed well with reasonably good predictive value and deployed it as a public web-based symptom checker (ispysti.org).

### Machine learning model development

**Fig. 1:**
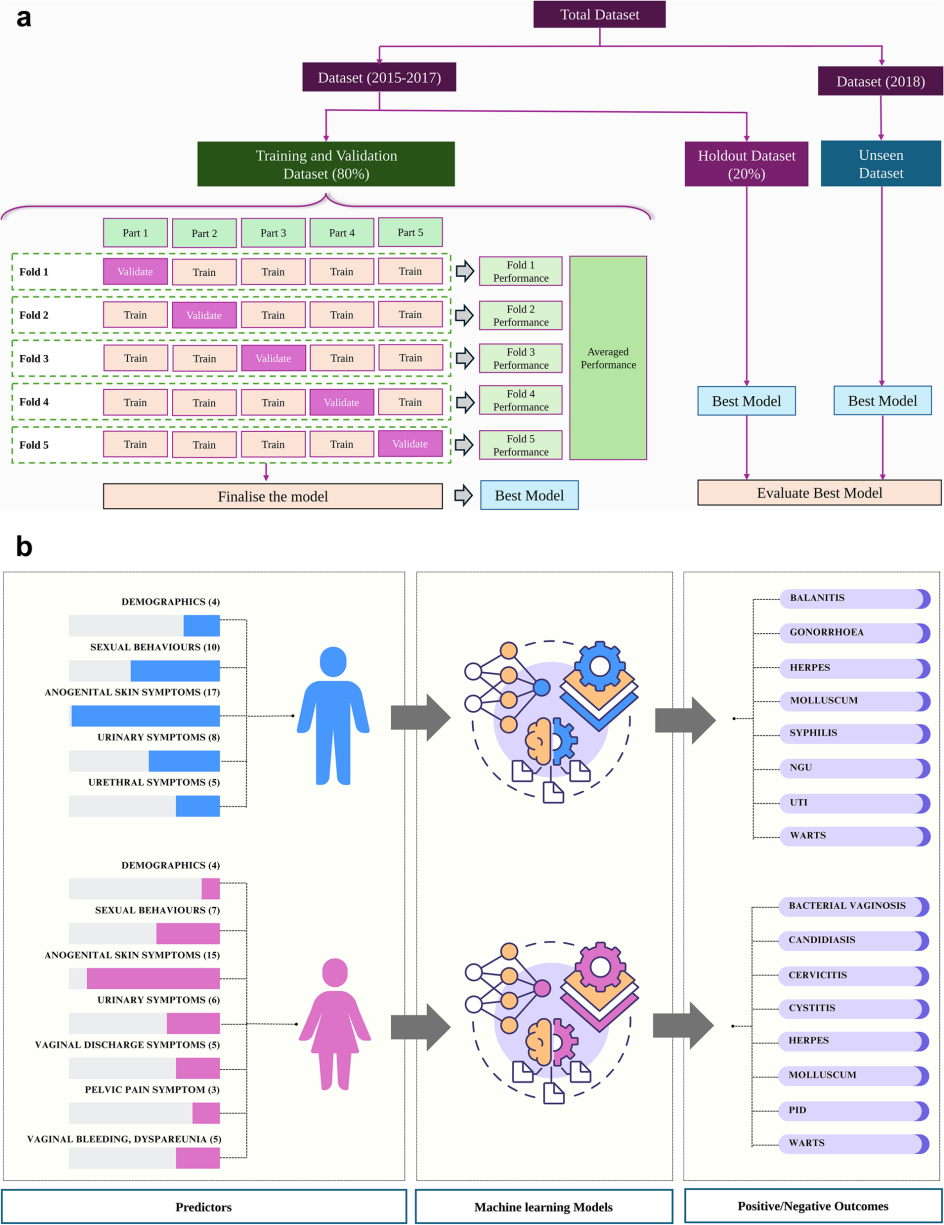
Workflows for model development, evaluation and predictions

First, we divided the main dataset into two subsets based on the date of data collection: one subset containing data from 2015 to 2017, and the other containing data from 2018 (Fig. 1A). The 2015–2017 dataset was further divided into a training and validation set (80%) and a holdout set (20%). We used stratified random sampling to ensure proportionate inclusion of positive and negative cases in both sets. The 2018 dataset was kept as an unseen dataset for evaluating the final models.

We used the PyCaret python library [30] within the Jupyter Notebook environment. We trained binary classification models for each disease condition, classifying positive or negative outcomes for each disease condition based on the predictor variables (Fig. 1B). During the training process, we employed the Synthetic Minority Over-sampling Technique (SMOTE) to address the imbalance between the higher number of negative cases and the lower number of positive cases in the dataset [30]. We also implemented a 5-fold cross-validation technique, partitioning the training and validation set into five equal subsets. In each iteration, one subset was used as a validation set, while the model was trained on the remaining four subsets. We repeated this process five times and calculated the average area under the ROC curve (AUC) score to assess model performance. The AUC score is derived by plotting true positive rate (TPR) against false positive rate (FPR). It ranges from 0 (worst performance) to 1 (best performance), with scores below 0.5 indicating a random classification model.

We explored various machine learning algorithms and selected the top four models with the highest AUC scores. We also employed ensemble techniques, namely blending and stacking, to combine the predictions of these four models into a single ensemble model. We obtained six models for each disease condition. Then, we conducted the sensitivity analysis to examine the variation in AUC scores and corresponding 95% confidence intervals across these models.

### Machine learning model evaluation

To assess the robustness and generalizability of the six models, we evaluated their performance on the holdout set (testing) and the 2018 dataset (external testing). This approach could verify whether the models could consistently perform well on unseen data. We used a two-way analysis of variance (ANOVA) without replication to calculate F-scores and *p*-values to identify significant differences in mean AUC scores between the different models, as well as to examine the variation in AUC scores across validation, testing and external testing. Then, we selected the best performing model with the highest AUC scores for each disease condition.

### Machine learning model interpretation

We used the SHapely Additive exPlanations (SHAP) library [31, 32] to examine how the models utilized important predictors for making final predictions and to assess whether their decision-making process aligned with clinicians. For each disease condition, we generated summary plots using SHAP, and these visualizations were reviewed and validated by experienced sexual health clinicians. Then, we identified the most influential predictor variables for top-performing models corresponding to each disease condition.

### Performance comparison between machine learning and bayesian models

We conducted a comparative analysis to assess the performance of the top machine learning models against Bayesian models currently deployed in the iSpySTI, using unseen testing datasets (2018 data and 20% holdout data, respectively). We performed bootstrapping 1,000 times on the unseen dataset to calculate the 95% confidence level for the performance indicators (AUC, accuracy, sensitivity, specificity, precision and F-1 scores). To compare the AUC scores between the machine learning and Bayesian models, we used Z-test, with standard errors obtained from the bootstrap confidence intervals. We also conducted the visual comparison of precision-recall curves and sensitivity-specificity curves for a comprehensive comparison between the machine learning and Bayesian models. Precision-recall curves show the balance between correctly identifying positive cases (positive predictive value) and finding all positive cases (sensitivity) at various thresholds. Sensitivity-specificity curves show the trade-off between sensitivity and specificity at different thresholds.

## Results

### Demographic characteristics and presenting symptoms

The median age of men was 30 (IQR: 26–38) and women was 27 years (IQR: 24–31) (Tables 1 and 2). Around two-fifths (2492/6121) were men who have sex with men (MSM). Consistent condom in the past 12 months use was reported by less than one-third of men with male partners, and 17% of the men with female partners within the past 12 months. Nearly 13% (508/4358) of female participants reported consistent condom use with casual male partners.

**Table 1:** Demographic characteristics and presenting symptoms by diagnosis in male clients

Male	Total (*N* = 6121)	Syphilis (*n* = 92, 1.5%)	Warts (*n* = 718, 11.7%)	UTI (*n* = 12, 0.2%)	Urethritis (*n* = 1447, 23.6%)	Urethral Gonorrhoea (*n* = 219, 3.6%)	Herpes (*n* = 249, 4.1%)	Balanitis (*n* = 546, 8.9%)	Molluscum Contagiosum (*n* = 168, 2.7%)
**I.** **Demographics and sexual behaviours**									
**Age (Median**,** IQR)**	30 (26–38)	32 (28–42)	30 (25–37)	46 (33–59)	31 (26–40)	31 (26–38)	31 (26–41)	29 (25–38)	28 (24–35)
** Country of birth**									
Australia or New Zealand	3312 (54.1%)	60 (65.2%)	390 (54.3%)	8 (66.7%)	771 (53.3%)	108 (49.3%)	127 (51.0%)	295 (54.0%)	94 (56.0%)
Other	2552 (41.7%)	28 (30.4%)	303 (42.2%)	3 (25.0%)	618 (42.7%)	103 (47.0%)	114 (45.8%)	242 (44.3%)	64 (38.1%)
Prefer not to answer	257 (4.2%)	4 (4.4%)	25 (3.5%)	1 (8.3%)	58 (4.0%)	8 (3.7%)	8 (3.2%)	9 (1.7%)	10 (6.0%)
** Recent arrival to Australia**									
Within 1 year	183 (7.2%)	0 (0.0%)	23 (7.4%)	0 (0.0%)	52 (8.4%)	10 (10.0%)	8 (7.0%)	15 (6.2%)	4 (6.3%)
1–5 years	1110 (43.4%)	8 (32.0%)	150 (48.2%)	0 (0.0%)	275 (44.6%)	52 (52.0%)	50 (43.9%)	98 (40.7%)	33 (51.6%)
> 5 years	1267 (49.5%)	17 (68.0%)	138 (44.4%)	3 (100.0%)	290 (47.0%)	38 (38.0%)	56 (49.1%)	128 (53.1%)	27 (42.2%)
** Had sex oversea within 1 year**									
Yes	2221 (38.2%)	27 (32.5%)	247 (36.2%)	3 (25.0%)	581 (42.0%)	76 (36.7%)	91 (38.4%)	205 (38.8%)	63 (38.2%)
No	3396 (58.3%)	48 (57.8%)	408 (59.8%)	9 (75.0%)	786 (56.8%)	130 (62.8%)	142 (59.9%)	313 (59.3%)	97 (58.8%)
Prefer not to answer	204 (3.5%)	8 (9.6%)	27 (4.0%)	0 (0.0%)	16 (1.2%)	1 (0.5%)	4 (1.7%)	10 (1.9%)	5 (3.0%)
** Have ever had sex with men**									
Yes	2492 (40.7%)	69 (75.0%)	221 (30.8%)	6 (50.0%)	641 (44.3%)	149 (68.0%)	110 (44.2%)	111 (20.3%)	28 (16.7%)
No	3629 (59.3%)	23 (25.0%)	497 (69.2%)	6 (50.0%)	806 (55.7%)	70 (32.0%)	139 (55.8%)	435 (79.7%)	140 (83.3%)
** Number of sexual partners within past 12 months (Median**,** IQR)**									
Men	6 (3–15)	10 (5–20)	6 (2–11)	5 (1–6)	7 (3–20)	4 (2–10)	6 (3–20)	5 (2–10)	6 (2–20)
Women	4 (2–6)	3 (2–5)	3 (2–6)	5 (3–5)	4 (2–7)	5 (3–8)	4 (2–8)	4 (2–6)	5 (2–7)
** Condom use with male partners in the past 12 months**									
Always/Usually	624 (29.4%)	10 (17.0%)	56 (28.6%)	1 (25.0%)	118 (21.3%)	24 (17.5%)	31 (32.0%)	34 (39.1%)	13 (50.0%)
Sometimes	1286 (60.5%)	40 (67.8%)	115 (58.7%)	2 (50.0%)	375 (67.7%)	101 (73.7%)	58 (59.8%)	40 (46.0%)	11 (42.3%)
Never	215 (10.1%)	9 (15.3%)	25 (12.8%)	1 (25.0%)	61 (11.0%)	12 (8.8%)	8 (8.3%)	13 (14.9%)	2 (7.7%)
Not Applicable/ Unknown	3911 (0.0%)	31 (0.0%)	516 (0.0%)	8 (0.0%)	870 (0.0%)	74 (0.0%)	150 (0.0%)	457 (0.0%)	142 (0.0%)
** Condom use with female partners in the past 12 months**									
Always/Usually	633 (16.7%)	4 (16.0%)	68 (14.0%)	1 (11.1%)	131 (14.7%)	17 (20.2%)	14 (9.2%)	78 (17.5%)	30 (21.4%)
Sometimes	2598 (68.5%)	18 (72.0%)	324 (66.8%)	8 (88.9%)	649 (72.9%)	61 (72.6%)	107 (69.9%)	305 (68.2%)	98 (70.0%)
Never	564 (14.9%)	3 (12.0%)	93 (19.2%)	0 (0.0%)	110 (12.4%)	6 (7.1%)	32 (20.9%)	64 (14.3%)	12 (8.6%)
Not Applicable/ Unknown	2249 (0.0%)	63 (0.0%)	223 (0.0%)	3 (0.0%)	542 (0.0%)	130 (0.0%)	95 (0.0%)	94 (0.0%)	28 (0.0%)
**II.** **Anogenital skin symptoms presented (more than 1 symptom may present)**									
** Itch**									
Yes	1987 (32.5%)	23 (25.0%)	109 (15.2%)	2 (16.7%)	593 (41.0%)	71 (32.4%)	65 (26.1%)	294 (53.9%)	28 (16.7%)
No	4134 (67.5%)	69 (75.0%)	609 (84.8%)	10 (83.3%)	854 (59.0%)	148 (67.6%)	184 (73.9%)	252 (46.2%)	140 (83.3%)
** Rash**									
Yes	1043 (17.0%)	15 (16.3%)	58 (8.1%)	1 (8.3%)	139 (9.6%)	23 (10.5%)	44 (17.7%)	238 (43.6%)	17 (10.1%)
No	5078 (83.0%)	77 (83.7%)	660 (91.9%)	11 (91.7%)	1308 (90.4%)	196 (89.5%)	205 (82.3%)	308 (56.4%)	151 (89.9%)
** Lumps**									
Yes	1163 (19.0%)	19 (20.7%)	409 (57.0%)	2 (16.7%)	80 (5.5%)	10 (4.6%)	47 (18.9%)	43 (7.9%)	77 (45.8%)
No	4958 (81.0%)	73 (79.4%)	309 (43.0%)	10 (83.3%)	1367 (94.5%)	209 (95.4%)	202 (81.1%)	503 (92.1%)	91 (54.2%)
** Spots**									
Yes	1260 (20.6%)	29 (31.5%)	172 (24.0%)	1 (8.3%)	108 (7.5%)	14 (6.4%)	63 (25.3%)	216 (39.6%)	77 (45.8%)
No	4861 (79.4%)	63 (68.5%)	546 (76.0%)	11 (91.7%)	1339 (92.5%)	205 (93.6%)	186 (74.7%)	330 (60.4%)	91 (54.2%)
** Blisters/sores**									
Yes	763 (12.5%)	49 (53.3%)	63 (8.8%)	2 (16.7%)	111 (7.7%)	23 (10.5%)	129 (51.8%)	74 (13.6%)	27 (16.1%)
No	5358 (87.5%)	43 (46.7%)	655 (91.2%)	10 (83.3%)	1336 (92.3%)	196 (89.5%)	120 (48.2%)	472 (86.5%)	141 (83.9%)
** No anogenital skin symptoms**									
Yes	1804 (29.5%)	12 (13.0%)	115 (16.0%)	7 (58.3%)	654 (45.2%)	110 (50.2%)	49 (19.7%)	34 (6.2%)	9 (5.4%)
No	4317 (70.5%)	80 (87.0%)	603 (84.0%)	5 (41.7%)	793 (54.8%)	109 (49.8%)	200 (80.3%)	512 (93.8%)	159 (94.6%)
** Site**									
Around the bottom or anus	482 (11.2%)	6 (7.5%)	111 (18.4%)	0 (0.0%)	33 (4.2%)	3 (2.8%)	34 (17.0%)	9 (1.8%)	4 (2.5%)
Groin crease	500 (11.6%)	8 (10.0%)	60 (10.0%)	1 (20.0%)	38 (4.8%)	8 (7.3%)	16 (8.0%)	13 (2.5%)	60 (37.7%)
Glans penis	2067 (47.9%)	45 (56.3%)	183 (30.4%)	4 (80.0%)	567 (71.5%)	87 (79.8%)	77 (38.5%)	445 (86.9%)	12 (7.6%)
Shaft of penis	933 (21.6%)	15 (18.8%)	217 (36.0%)	0 (0.0%)	112 (14.1%)	8 (7.3%)	67 (33.5%)	33 (6.5%)	72 (45.3%)
Testicles or scrotum	335 (7.8%)	6 (7.5%)	32 (5.3%)	0 (0.0%)	43 (5.4%)	3 (2.8%)	6 (3.0%)	12 (2.3%)	11 (6.9%)
Not applicable	1804 (0.0%)	12 (0.0%)	115 (0.0%)	7 (0.0%)	654 (0.0%)	110 (0.0%)	49 (0.0%)	34 (0.0%)	9 (0.0%)
** Pain level of blisters/sores**									
Very painful	65 (8.5%)	9 (18.4%)	4 (6.4%)	0 (0.0%)	15 (13.5%)	4 (17.4%)	20 (15.5%)	5 (6.8%)	0 (0.0%)
A bit painful	389 (51.0%)	27 (55.1%)	21 (33.3%)	2 (100.0%)	71 (64.0%)	18 (78.3%)	80 (62.0%)	35 (47.3%)	8 (29.6%)
Not painful	309 (40.5%)	13 (26.5%)	38 (60.3%)	0 (0.0%)	25 (22.5%)	1 (4.4%)	29 (22.5%)	34 (46.0%)	19 (70.4%)
Not applicable	5358 (0.0%)	13 (0.0%)	655 (0.0%)	10 (0.0%)	1336 (0.0%)	196 (0.0%)	120 (0.0%)	472 (0.0%)	141 (0.0%)
**III.** **Urethral symptoms presented**									
** Pain/discomfort at urethra**									
Yes	1713 (28.0%)	19 (20.7%)	49 (6.8%)	8 (66.7%)	1019 (70.4%)	168 (76.7%)	62 (24.9%)	130 (23.8%)	13 (7.7%)
No	4408 (72.0%)	73 (79.4%)	669 (93.2%)	4 (33.3%)	428 (29.6%)	51 (23.3%)	187 (75.1%)	416 (76.2%)	155 (92.3%)
** Itch at urethra**									
Yes	1277 (20.9%)	22 (23.9%)	60 (8.4%)	1 (8.3%)	542 (37.5%)	61 (27.9%)	30 (12.1%)	238 (43.6%)	10 (6.0%)
No	4844 (79.1%)	70 (76.1%)	658 (91.6%)	11 (91.7%)	905 (62.5%)	158 (72.2%)	219 (88.0%)	308 (56.4%)	158 (94.1%)
** Penile discharge**									
Yes	656 (10.7%)	4 (4.4%)	18 (2.5%)	2 (16.7%)	496 (34.3%)	167 (76.3%)	6 (2.4%)	46 (8.4%)	4 (2.4%)
No	5465 (89.3%)	88 (95.7%)	700 (97.5%)	10 (83.3%)	951 (65.7%)	52 (23.7%)	243 (97.6%)	500 (91.6%)	164 (97.6%)
** No urethral symptoms**									
Yes	3497 (57.1%)	61 (66.3%)	620 (86.4%)	2 (16.7%)	108 (7.5%)	8 (3.7%)	168 (67.5%)	227 (41.6%)	148 (88.1%)
No	2624 (42.9%)	31 (33.7%)	98 (13.7%)	10 (83.3%)	1339 (92.5%)	211 (96.4%)	81 (32.5%)	319 (58.4%)	20 (11.9%)
** Discharge type**									
Thickish, green or yellow (Onset: 1–2 days after sex)	306 (46.7%)	3 (75.0%)	6 (33.3%)	1 (50.0%)	257 (51.8%)	140 (83.8%)	1 (16.7%)	12 (26.1%)	1 (25.0%)
Thin, clear or cloudy (Onset: >5 days after sex)	350 (53.4%)	1 (25.0%)	12 (66.7%)	1 (50.0%)	239 (48.2%)	27 (16.2%)	5 (83.3%)	34 (73.9%)	3 (75.0%)
Not applicable	5465 (0.0%)	88 (0.0%)	0 (0.0%)	10 (0.0%)	(0.0%)	(0.0%)	243 (0.0%)	(0.0%)	164 (0.0%)
**IV.** **Urinary symptoms presented**									
** Painful or discomfort urination**									
Yes	1448 (23.7%)	15 (16.3%)	36 (5.0%)	7 (58.3%)	959 (66.3%)	174 (79.5%)	54 (21.7%)	99 (18.1%)	13 (7.7%)
No	4673 (76.3%)	77 (83.7%)	682 (95.0%)	5 (41.7%)	488 (33.7%)	45 (20.6%)	195 (78.3%)	447 (81.9%)	155 (92.3%)
** Urge or frequent urination**									
Yes	736 (12.0%)	8 (8.7%)	18 (2.5%)	7 (58.3%)	469 (32.4%)	69 (31.5%)	20 (8.0%)	54 (9.9%)	0 (0.0%)
No	5385 (88.0%)	84 (91.3%)	700 (97.5%)	5 (41.7%)	978 (67.6%)	150 (68.5%)	229 (92.0%)	492 (90.1%)	168 (100.0%)
** Blood in urine**									
Yes	54 (0.9%)	1 (1.1%)	1 (0.1%)	1 (8.3%)	33 (2.3%)	6 (2.7%)	2 (0.8%)	5 (0.9%)	1 (0.6%)
No	6067 (99.1%)	91 (98.9%)	717 (99.9%)	11 (91.7%)	1414 (97.7%)	213 (97.3%)	247 (99.2%)	541 (99.1%)	167 (99.4%)
** Associated with fever**									
Yes	253 (4.1%)	7 (7.6%)	9 (1.3%)	2 (16.7%)	137 (9.5%)	21 (9.6%)	9 (3.6%)	20 (3.7%)	2 (1.2%)
No	5868 (95.9%)	85 (92.4%)	709 (98.8%)	10 (83.3%)	1310 (90.5%)	198 (90.4%)	240 (96.4%)	526 (96.3%)	166 (98.8%)
** Pain or discomfort level during urination**									
Mild	1304 (68.0%)	7 (35.0%)	40 (70.2%)	2 (25.0%)	764 (66.7%)	105 (56.2%)	31 (47.0%)	112 (73.7%)	11 (78.6%)
Moderate	519 (27.1%)	10 (50.0%)	13 (22.8%)	5 (62.5%)	327 (28.5%)	65 (34.8%)	25 (37.9%)	32 (21.1%)	2 (14.3%)
Severe	75 (3.9%)	3 (15.0%)	3 (5.3%)	1 (12.5%)	48 (4.2%)	17 (9.1%)	9 (13.6%)	6 (4.0%)	0 (0.0%)
Unbearable	19 (1.0%)	0 (0.0%)	1 (1.8%)	0 (0.0%)	7 (0.6%)	0 (0.0%)	1 (1.5%)	2 (1.3%)	1 (7.1%)
**Duration of urinary symptoms (days) (Median**,** IQR)**	7 (3–14)	14 (7–28)	14 (4–60)	5 (3–9)	6 (3–14)	3 (2–5)	5 (3–7)	14 (5–30)	12 (7–28)

**Table 2:** Demographic characteristics and presenting symptoms by diagnosis in female clients

Female	Total (*N* = 4,358)	PID (*n* = 281, 6.4%)	Cervicitis (*n* = 66, 1.5%)	Warts (*n* = 191, 4.4%)	Herpes (*n* = 174, 4.0%)	UTI Cystitis (*n* = 272, 6.2%)	Bacterial Vaginosis (*n* = 707, 16.2%)	Candidiasis (*n* = 722, 16.6%)	Molluscum Contagiosum (*n* = 55, 1.3%)
**I.** **Demographics and sexual behaviours**									
Age (Median, IQR)	27 (24–31)	26 (23–30)	26 (24–29)	27 (23–32)	26 (24–31)	26 (23–30)	27 (24–31)	26 (23–30)	25 (24–30)
** Country of birth**									
Australia or New Zealand	1680 (38.6%)	93 (33.1%)	24 (36.4%)	55 (28.8%)	64 (36.8%)	91 (33.5%)	254 (35.9%)	262 (36.3%)	24 (43.6%)
Other	2486 (57.0%)	176 (62.6%)	41 (62.1%)	127 (66.5%)	101 (58.1%)	168 (61.8%)	416 (58.8%)	426 (59.0%)	30 (54.6%)
Unknown	192 (4.4%)	12 (4.3%)	1 (1.5%)	9 (4.7%)	9 (5.2%)	13 (4.8%)	37 (5.2%)	34 (4.7%)	1 (1.8%)
** Arrival to Australia**									
Within 1 year	251 (10.3%)	22 (12.6%)	2 (5.0%)	10 (8.1%)	17 (16.8%)	21 (12.5%)	50 (12.2%)	49 (11.8%)	2 (6.9%)
1–5 years	1535 (63.1%)	104 (59.8%)	30 (75.0%)	85 (68.6%)	62 (61.4%)	98 (58.3%)	270 (65.9%)	261 (62.7%)	22 (75.9%)
> 5 years	647 (26.6%)	48 (27.6%)	8 (20.0%)	29 (23.4%)	22 (21.8%)	49 (29.2%)	90 (22.0%)	106 (25.5%)	5 (17.2%)
** Sex oversea within 1 year**									
Yes	1744 (40.0%)	121 (43.1%)	26 (39.4%)	80 (41.9%)	63 (36.2%)	112 (41.2%)	309 (43.7%)	301 (41.7%)	22 (40.0%)
No	2330 (53.5%)	147 (52.3%)	35 (53.0%)	98 (51.3%)	104 (59.8%)	146 (53.7%)	354 (50.1%)	385 (53.3%)	28 (50.9%)
Prefer not to answer	284 (6.5%)	13 (4.6%)	5 (7.6%)	13 (6.8%)	7 (4.0%)	14 (5.2%)	44 (6.2%)	36 (5.0%)	5 (9.1%)
** Number of casual sexual partners within past 12 months**									
Men	3 (2–5)	3 (2–6)	3 (2–5)	3 (2–5)	3 (2–5)	3 (2–5)	4 (2–6)	3 (2–5)	3 (1–6)
Women	1 (1–3)	1 (1–2)	1 (1–3)	1 (1–3)	1 (1–2)	2 (1–3)	3 (1–3)	2 (1–3)	1 (1–2)
** Condom use with male partners in the past 12 months**									
Always/Usually	508 (12.7%)	24 (8.9%)	5 (8.2%)	25 (14.0%)	16 (9.8%)	30 (11.6%)	59 (8.9%)	84 (12.4%)	13 (25.5%)
Sometimes	2950 (74.0%)	208 (77.0%)	51 (83.6%)	129 (72.1%)	119 (73.0%)	188 (72.6%)	510 (77.3%)	522 (77.0%)	35 (68.6%)
Never	530 (13.3%)	38 (14.1%)	5 (8.2%)	25 (14.0%)	28 (17.2%)	41 (15.8%)	91 (13.8%)	72 (10.6%)	3 (5.9%)
Not Applicable/ Unknown	370 (0.0%)	11 (0.0%)	5 (0.0%)	12 (0.0%)	11 (0.0%)	13 (0.0%)	47 (0.0%)	(0.0%)	(0.0%)
** Condom use with female partners in the past 12 months**									
Always/Usually	9 (10.2%)	1 (12.5%)	0 (0.0%)	0 (0.0%)	0 (0.0%)	1 (25.0%)	4 (14.3%)	1 (9.1%)	0 (0.0%)
Sometimes	4 (4.6%)	3 (37.5%)	0 (0.0%)	0 (0.0%)	1 (16.7%)	0 (0.0%)	3 (10.7%)	0 (0.0%)	0 (0.0%)
Never	75 (85.2%)	4 (50.0%)	1 (100.0%)	1 (100.0%)	5 (83.3%)	3 (75.0%)	21 (75.0%)	10 (90.9%)	2 (100.0%)
Not Applicable/ Unknown	4270 (0.0%)	273 (0.0%)	65 (0.0%)	190 (0.0%)	168 (0.0%)	268 (0.0%)	679 (0.0%)	711 (0.0%)	53 (0.0%)
**II.** **Anogenital skin symptoms presented**									
** Itch**									
Yes	1927 (44.2%)	97 (34.5%)	34 (51.5%)	61 (31.9%)	92 (52.9%)	90 (33.1%)	281 (39.8%)	581 (80.5%)	17 (30.9%)
No	2431 (55.8%)	184 (65.5%)	32 (48.5%)	130 (68.1%)	82 (47.1%)	182 (66.9%)	426 (60.3%)	141 (19.5%)	38 (69.1%)
** Rash**									
Yes	454 (10.4%)	21 (7.5%)	9 (13.6%)	17 (8.9%)	38 (21.8%)	19 (7.0%)	62 (8.8%)	134 (18.6%)	6 (10.9%)
No	3904 (89.6%)	260 (92.5%)	57 (86.4%)	174 (91.1%)	136 (78.2%)	253 (93.0%)	645 (91.2%)	588 (81.4%)	49 (89.1%)
** Lumps**									
Yes	729 (16.7%)	26 (9.3%)	4 (6.1%)	115 (60.2%)	70 (40.2%)	12 (4.4%)	72 (10.2%)	98 (13.6%)	36 (65.5%)
No	3629 (83.3%)	255 (90.8%)	62 (93.9%)	76 (39.8%)	104 (59.8%)	260 (95.6%)	635 (89.8%)	624 (86.4%)	19 (34.6%)
** Spots**									
Yes	431 (9.9%)	23 (8.2%)	7 (10.6%)	49 (25.7%)	38 (21.8%)	11 (4.0%)	46 (6.5%)	52 (7.2%)	24 (43.6%)
No	3927 (90.1%)	258 (91.8%)	59 (89.4%)	142 (74.4%)	136 (78.2%)	261 (96.0%)	661 (93.5%)	670 (92.8%)	31 (56.4%)
** Blisters/sores**									
Yes	411 (9.4%)	16 (5.7%)	3 (4.6%)	21 (11.0%)	95 (54.6%)	18 (6.6%)	44 (6.2%)	64 (8.9%)	6 (10.9%)
No	3947 (90.6%)	265 (94.3%)	63 (95.5%)	170 (89.0%)	79 (45.4%)	254 (93.4%)	663 (93.8%)	658 (91.1%)	49 (89.1%)
** No anogenital skin symptoms**									
Yes	1696 (38.9%)	157 (55.9%)	30 (45.5%)	21 (11.0%)	6 (3.5%)	158 (58.1%)	353 (49.9%)	91 (12.6%)	2 (3.6%)
No	2662 (61.1%)	124 (44.1%)	36 (54.6%)	170 (89.0%)	168 (96.6%)	114 (41.9%)	354 (50.1%)	631 (87.4%)	53 (96.4%)
** Site**									
Around the bottom or anus	260 (9.8%)	10 (8.1%)	2 (3.0%)	39 (22.9%)	27 (16.1%)	7 (6.1%)	27 (7.6%)	28 (4.4%)	11 (20.8%)
Groin crease	116 (4.4%)	5 (4.0%)	1 (1.5%)	3 (1.8%)	6 (3.6%)	2 (1.8%)	12 (3.4%)	9 (1.4%)	11 (20.8%)
Pubic hair area	186 (7.0%)	5 (4.0%)	21 (31.8%)	7 (4.1%)	10 (6.0%)	8 (7.0%)	24 (6.8%)	15 (2.4%)	15 (28.3%)
Vulva	1413 (53.1%)	65 (52.4%)	12 (18.2%)	97 (57.1%)	106 (63.1%)	43 (37.7%)	172 (48.6%)	378 (59.9%)	15 (28.3%)
Inside vagina	687 (25.8%)	39 (31.5%)	30 (45.5%)	24 (14.1%)	19 (11.3%)	54 (47.4%)	119 (33.6%)	201 (31.9%)	1 (1.9%)
** Pain level of blisters/sores**									
Very painful	86 (20.9%)	4 (25.0%)	1 (33.3%)	3 (14.3%)	36 (37.9%)	3 (16.7%)	5 (11.4%)	12 (18.8%)	0 (0.0%)
A bit painful	254 (61.8%)	10 (62.5%)	2 (66.7%)	10 (47.6%)	55 (57.9%)	14 (77.8%)	27 (61.4%)	41 (64.1%)	4 (66.7%)
Not painful	71 (17.3%)	2 (12.5%)	0 (0.0%)	8 (38.1%)	4 (4.2%)	1 (5.6%)	12 (27.3%)	11 (17.2%)	2 (33.3%)
**III. Vaginal discharge symptoms presented**									
** Unusual vaginal discharge**									
Yes	2029 (46.6%)	158 (56.2%)	42 (63.6%)	47 (24.6%)	73 (42.0%)	101 (37.1%)	465 (65.8%)	474 (65.7%)	13 (23.6%)
No	2329 (53.4%)	123 (43.8%)	24 (36.4%)	144 (75.4%)	101 (58.1%)	171 (62.9%)	242 (34.2%)	248 (34.4%)	42 (76.4%)
** Unusual smell**									
Yes	1381 (31.7%)	111 (39.5%)	30 (45.5%)	39 (20.4%)	51 (29.3%)	69 (25.4%)	494 (69.9%)	239 (33.1%)	11 (20.0%)
No	2977 (68.3%)	170 (60.5%)	36 (54.6%)	152 (79.6%)	123 (70.7%)	203 (74.6%)	213 (30.1%)	483 (66.9%)	44 (80.0%)
** No vaginal discharge symptoms**									
Yes	1869 (42.9%)	94 (33.5%)	16 (24.2%)	121 (63.4%)	76 (43.7%)	143 (52.6%)	97 (13.7%)	199 (27.6%)	40 (72.7%)
No	2489 (57.1%)	187 (66.6%)	50 (75.8%)	70 (36.7%)	98 (56.3%)	129 (47.4%)	610 (86.3%)	523 (72.4%)	15 (27.3%)
** Discharge type**									
Thickish, clumpy or white	988 (49.7%)	75 (49.3%)	13 (29.6%)	28 (52.8%)	35 (48.6%)	45 (49.5%)	140 (26.2%)	306 (67.4%)	6 (46.2%)
Pale or greyish discharge with fishy smell or worse after sex	1002 (50.4%)	77 (50.7%)	31 (70.5%)	25 (47.2%)	37 (51.4%)	46 (50.6%)	395 (73.8%)	148 (32.6%)	7 (53.9%)
** Discharge associated with itch**									
Yes	1460 (58.7%)	98 (52.4%)	29 (58.0%)	46 (65.7%)	68 (69.4%)	72 (55.8%)	296 (48.5%)	451 (86.2%)	4 (26.7%)
No	1028 (41.3%)	89 (47.6%)	21 (42.0%)	24 (34.3%)	30 (30.6%)	57 (44.2%)	314 (51.5%)	72 (13.8%)	11 (73.3%)
**IV. Pain in pelvis or genital area**									
Yes	2050 (47.0%)	236 (84.0%)	37 (56.1%)	43 (22.5%)	117 (67.2%)	184 (67.7%)	306 (43.3%)	349 (48.3%)	8 (14.6%)
No	2308 (53.0%)	45 (16.0%)	29 (43.9%)	148 (77.5%)	57 (32.8%)	88 (32.4%)	401 (56.7%)	373 (51.7%)	47 (85.5%)
** Pain location**									
Inside vagina	389 (21.2%)	32 (15.0%)	6 (18.8%)	4 (11.1%)	19 (17.8%)	52 (31.7%)	62 (22.4%)	78 (25.2%)	2 (28.6%)
Pelvis/lower part of tummy	975 (53.1%)	166 (77.9%)	19 (59.4%)	20 (55.6%)	17 (15.9%)	85 (51.8%)	155 (56.0%)	102 (32.9%)	3 (42.9%)
Vulva	473 (25.8%)	15 (7.0%)	7 (21.9%)	12 (33.3%)	71 (66.4%)	27 (16.5%)	60 (21.7%)	130 (41.9%)	2 (28.6%)
** Level of pain**									
Mild	1058 (51.6%)	101 (42.8%)	16 (43.2%)	24 (55.8%)	33 (28.2%)	72 (39.1%)	161 (52.6%)	173 (49.6%)	4 (50.0%)
Moderate	779 (38.0%)	108 (45.8%)	19 (51.4%)	15 (34.9%)	57 (48.7%)	85 (46.2%)	115 (37.6%)	138 (39.5%)	3 (37.5%)
Severe	183 (8.9%)	24 (10.2%)	1 (2.7%)	4 (9.3%)	26 (22.2%)	24 (13.0%)	25 (8.2%)	34 (9.7%)	1 (12.5%)
Unbearable	30 (1.5%)	3 (1.3%)	1 (2.7%)	0 (0.0%)	1 (0.9%)	3 (1.6%)	5 (1.6%)	4 (1.2%)	(0.0%)
**V. Symptoms of vaginal bleeding or dyspareunia**									
** Bleeding from vagina after sex**									
Yes	514 (11.8%)	61 (21.7%)	13 (19.7%)	8 (4.2%)	13 (7.5%)	42 (15.4%)	103 (14.6%)	69 (9.6%)	3 (5.5%)
No	3844 (88.2%)	220 (78.3%)	53 (80.3%)	183 (95.8%)	161 (92.5%)	230 (84.6%)	604 (85.4%)	653 (90.4%)	52 (94.6%)
** Bleeding or spotting between periods**									
Yes	709 (16.3%)	70 (24.9%)	11 (16.7%)	19 (10.0%)	26 (14.9%)	47 (17.3%)	118 (16.7%)	92 (12.7%)	5 (9.1%)
No	3649 (83.7%)	211 (75.1%)	55 (83.3%)	172 (90.1%)	148 (85.1%)	225 (82.7%)	589 (83.3%)	630 (87.3%)	50 (90.9%)
** Pain during sex**									
Yes	1179 (27.1%)	142 (50.5%)	22 (33.3%)	25 (13.1%)	52 (29.9%)	94 (34.6%)	192 (27.2%)	262 (36.3%)	6 (10.9%)
No	3179 (73.0%)	139 (49.5%)	44 (66.7%)	166 (86.9%)	122 (70.1%)	178 (65.4%)	515 (72.8%)	460 (63.7%)	49 (89.1%)
** No vaginal bleeding or dyspareunia symptoms**									
Yes	2460 (56.5%)	82 (29.2%)	32 (48.5%)	147 (77.0%)	100 (57.5%)	128 (47.1%)	392 (55.5%)	356 (49.3%)	42 (76.4%)
No	1898 (43.6%)	199 (70.8%)	34 (51.5%)	44 (23.0%)	74 (42.5%)	144 (52.9%)	315 (44.6%)	366 (50.7%)	13 (23.6%)
** Location of pain during sex**									
Pelvis/lower part of tummy	450 (38.2%)	97 (68.3%)	8 (36.4%)	9 (36.0%)	10 (19.2%)	39 (41.5%)	86 (44.8%)	61 (23.3%)	0 (0.0%)
Vulva or vagina	729 (61.8%)	45 (31.7%)	14 (63.6%)	16 (64.0%)	42 (80.8%)	55 (58.5%)	106 (55.2%)	201 (76.7%)	6 (100.0%)
**VI. Urinary symptoms presented**									
** Painful or discomfort urination**									
Yes	1111 (25.5%)	86 (30.6%)	20 (30.3%)	25 (13.1%)	91 (52.3%)	216 (79.4%)	140 (19.8%)	246 (34.1%)	4 (7.3%)
No	3247 (74.5%)	195 (69.4%)	46 (69.7%)	166 (86.9%)	83 (47.7%)	56 (20.6%)	567 (80.2%)	476 (65.9%)	51 (92.7%)
** Urge or frequent urination**									
Yes	984 (22.6%)	97 (34.5%)	19 (28.8%)	25 (13.1%)	35 (20.1%)	190 (69.9%)	140 (19.8%)	159 (22.0%)	7 (12.7%)
No	3374 (77.4%)	184 (65.5%)	47 (71.2%)	166 (86.9%)	139 (79.9%)	82 (30.2%)	567 (80.2%)	563 (78.0%)	48 (87.3%)
** Blood in urine**									
Yes	153 (3.5%)	16 (5.7%)	2 (3.0%)	4 (2.1%)	4 (2.3%)	52 (19.1%)	19 (2.7%)	19 (2.6%)	1 (1.8%)
No	4205 (96.5%)	265 (94.3%)	64 (97.0%)	187 (97.9%)	170 (97.7%)	220 (80.9%)	688 (97.3%)	703 (97.4%)	54 (98.2%)
** Associated with fever**									
Yes	449 (10.3%)	52 (18.5%)	2 (3.0%)	8 (4.2%)	39 (22.4%)	55 (20.2%)	68 (9.6%)	75 (10.4%)	2 (3.6%)
No	3909 (89.7%)	229 (81.5%)	64 (97.0%)	183 (95.8%)	135 (77.6%)	217 (79.8%)	639 (90.4%)	647 (89.6%)	53 (96.4%)
** No urinary symptoms**									
Yes	2565 (58.9%)	123 (43.8%)	36 (54.6%)	148 (77.5%)	61 (35.1%)	20 (7.4%)	460 (65.1%)	363 (50.3%)	46 (83.6%)
No	1793 (41.1%)	158 (56.2%)	30 (45.5%)	43 (22.5%)	113 (64.9%)	252 (92.7%)	247 (34.9%)	359 (49.7%)	9 (16.4%)
**Duration of urinary symptoms (days) (Median**,** IQR)**	7 (4–21)	14 (7–30)	14 (6–21)	14 (6–29)	5 (3–7)	6 (3–14)	14 (4–30)	7 (4–14)	14 (2–49)

Among men (*N* = 6,121), NGU was the most common condition (*n* = 1447, 23.6%), followed by genital warts (*n* = 718, 11.7%) and balanitis (*n* = 546, 8.9%). UTI was the least common (*n* = 12, 0.2%). The remaining conditions ranged from 92 cases of primary syphilis (1.5%) to 249 cases of genital herpes (*n* = 249, 4.1%). Among women (*N* = 4,358), candidiasis (*n* = 722, 16.6%) and bacterial vaginosis (*n* = 707, 16.2%) were the most common conditions. The remaining conditions ranged from 55 cases of molluscum contagiosum (1.3%) to 272 cases of UTI/cystitis (6.2%). Anogenital skin lesions (itch, rash, lumps, spots, or blisters) were common, reported by 70.5% of men and 61.1% of women. Among them, itch was the most prevalent (32.5% in men, 44.2% in women), while blisters/sores were the least common (12.5% in men, 9.4% in women). Additionally, 42.9% of males reported urethral symptoms (pain or discharge), while 41.3% of women reported unusual vaginal discharge or smell. Nearly half (47.0%) of women reported pain in the pelvic or genital areas, and 41.1% reported urinary symptoms (pain, frequency, or urgency). Details are presented in Tables 1 and 2.

### Model performance during training and validation

Figures S1 and S2 present the performance comparison (mean AUC with 95% confidence intervals) of different machine learning models during model training and validation across various disease conditions. In the male dataset, the models demonstrated the highest predictive performance for urethral gonorrhoea (AUC 0.92), followed by NGU (0.91), molluscum contagiosum (0.89), genital warts (0.87), balanitis (0.86), syphilis (0.82), herpes (0.79), and the lowest for UTI (0.72). In the female dataset, the highest AUC was achieved for molluscum contagiosum (0.93), followed by herpes (0.86), cystitis and genital warts (0.83), candidiasis (0.78), bacterial vaginosis and PID (0.77), and the lowest for cervicitis (0.58).

### Model evaluation with unseen datasets

**Fig. 2:**
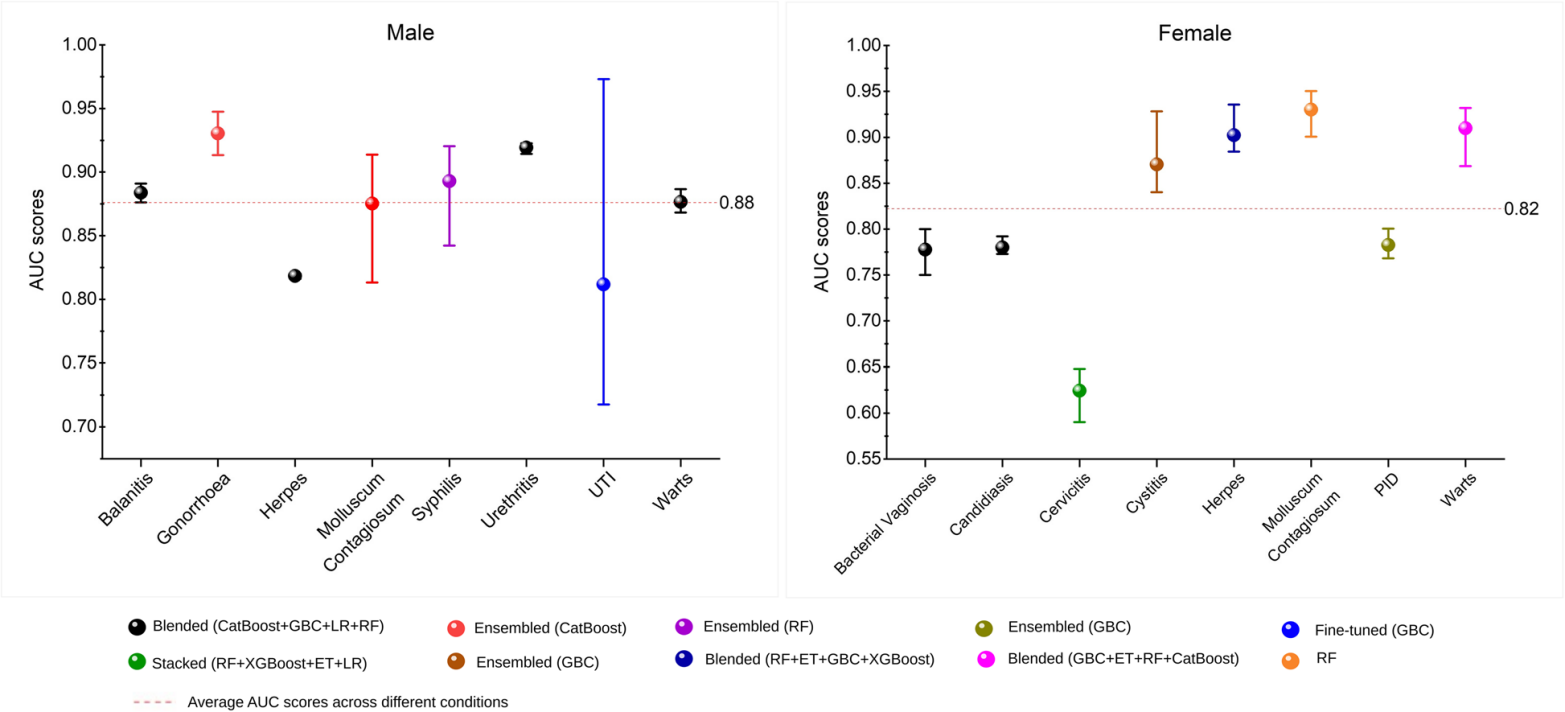
The AUC for the best performing machine learning models throughout validation, testing and external testing across different diseases

 The models’ predictive performance was evaluated across validation, testing and external testing datasets to identify the most consistently well-performing models. Figure 2 presents the best-performing models for each disease condition.

For male-specific disease conditions, the ensemble CatBoost model was the top performer for diagnosing urethral gonorrhoea (mean AUC 0.93, two-way ANOVA, *p* = 0.05) and molluscum contagiosum (AUC 0.88, *p* = 0.13). The blended model, combining CatBoost, Gradient Boosting Classifier (GBC), Logistic Regression (LR), and Random Forest (RF) algorithms, outperformed others for balanitis (AUC 0.88, *p* < 0.05), herpes (AUC 0.82, *p* = 0.60), NGU (AUC 0.92, *p* = 0.07), and genital warts (AUC 0.88, *p* < 0.05). For syphilis, the ensemble RF model outperformed other models (AUC 0.89, *p* < 0.05). However, models could not perform well for UTI due to limited positive cases. All models for male-specific conditions, except UTI, demonstrated robust performance with AUC scores ranging from 0.81 to 0.95 across datasets. The models’ performance varied across validation, testing and external testing datasets (*p* < 0.05) except for herpes (*p* = 0.43).

For female-specific conditions, the combination of models outperformed individual algorithms in bacterial vaginosis, candidiasis, genital herpes and genital warts. The blended model (CatBoost + GBC + LR + RF) outperformed other models for both bacterial vaginosis and candidiasis (AUC 0.78, *p* < 0.05). The blended model, combining RF, Extra Trees Classifier (ET), GBC, and Extreme Gradient Boosting (XGBoost) performed best for herpes (AUC 0.90, *p* < 0.05), while a similar blended model (GBC + ET + RF + CatBoost) performed best for genital warts (AUC 0.91, *p* < 0.05). The ensemble GBC achieved the best scores for both cystitis (AUC 0.87, *p* < 0.05) and PID (AUC 0.78, *p* < 0.05). The RF outperformed other models for molluscum contagiosum (AUC 0.93, *p* < 0.05). However, the models could not perform well for cervicitis with AUC scores lower than 0.65. All models for female-specific conditions, except cervicitis, demonstrated robust performance with AUC scores ranging from 0.75 to 0.95 across datasets. Tables S2 and S3 compare model performance during validation in detail.

### Model interpretation and key predictors

The SHAP analysis helped us understand the key predictors that increase the likelihood of positive outcomes for each predicted condition. The models identified a combination of demographic factors, sexual behaviours, symptoms, and clinical presentations that are important for different STIs and genital conditions. For urethral gonorrhoea, NGU, and UTIs, the presence of urethral discharge and urinary symptoms (such as dysuria, frequent urination, and urgency) were crucial predictors. For bacterial vaginosis and candidiasis, the characteristics of vaginal discharge (odour, colour) and associated symptoms like itchiness were important predictors. For PID and cervicitis, pelvic pain, dyspareunia, and unusual vaginal discharge with odour were relevant predictors. For conditions like molluscum contagiosum, genital warts, balanitis, herpes, and syphilis, the similarity to the images we presented, and location of the skin lesions, were important predictors. Risky sexual behaviours, such as having unprotected sex, multiple casual sexual partners, and being an MSM, were identified as important predictors for various STIs. The model interpretation showed that our models identified the most important symptoms for diagnosing each specific anogenital condition, which is similar to how a clinician considers symptoms according to the guidelines [33]. Detailed interpretations for each condition are presented in Figure S3.

### Performance comparison between machine learning and bayesian models

Both machine learning and Bayesian models demonstrated robust performance, with accuracies ranging from 0.68 to 0.99 and AUC values consistently over 0.75 (Table 3). Machine learning models were similar to Bayesian models for most conditions. There was no statistically significant difference machine learning models and Bayesian models except for male balanitis, molluscum contagiosum and genital warts (*p*-value < 0.05) where machine learning performed better. In contrast, Bayesian models performed better in predicting male urethral gonorrhoea, female PID and UTI (*p*-value > 0.05). The precision-recall and sensitivity-specificity curves (Figure S4) shows the trade-offs between key metrics (sensitivity, specificity, precision/positive predictive values) across various thresholds for machine learning and Bayesian models.

**Table 3:** Performance comparison between machine learning and bayesian network models

**Males**	**Model**	**Accuracy**	**AUC**	**Sensitivity/Recall**	**specificity**	**Precision/PPV**	**F1**	***P*-value** **(AUC)**
Balanitis^#^	BN	0.855 (0.846–0.864)	0.851 (0.831–0.869)	0.734 (0.698–0.771)	0.866 (0.857–0.876)	0.350 (0.324–0.379)	0.474 (0.446–0.503)	0.002
	ML	0.903 (0.886–0.920)	0.901 (0.875–0.926)	0.606 (0.516–0.697)	0.932 (0.917–0.946)	0.465 (0.386–0.545)	0.526 (0.450–0.598)	
Gonorrhoea	BN	0.968 (0.963–0.972)	0.924 (0.902–0.944)	0.534 (0.467–0.598)	0.984 (0.980–0.987)	0.547 (0.481–0.613)	0.540 (0.480–0.597)	0.835
	ML	0.969 (0.958–0.978)	0.918 (0.859–0.964)	0.750 (0.612–0.875)	0.977 (0.968–0.985)	0.550 (0.426–0.672)	0.635 (0.514–0.730)	
Herpes	BN	0.946 (0.940–0.951)	0.736 (0.698–0.772)	0.281 (0.225–0.340)	0.974 (0.970–0.977)	0.313 (0.250–0.370)	0.296 (0.238–0.349)	0.308
	ML	0.954 (0.941–0.965)	0.776 (0.709–0.844)	0.280 (0.158–0.419)	0.982 (0.974–0.989)	0.400 (0.241–0.567)	0.329 (0.195–0.457)	
Molluscum Contagiosum^#^	BN	0.946 (0.940–0.952)	0.852 (0.817–0.884)	0.458 (0.383–0.532)	0.960 (0.955–0.965)	0.244 (0.192–0.291)	0.318 (0.259–0.373)	0.022
	ML	0.937 (0.923–0.949)	0.918 (0.868–0.959)	0.765 (0.613–0.909)	0.942 (0.928–0.954)	0.274 (0.184–0.363)	0.403 (0.292–0.500)	
Syphilis	BN	0.977 (0.973–0.981)	0.858 (0.816–0.897)	0.261 (0.173–0.349)	0.988 (0.985–0.991)	0.250 (0.172–0.342)	0.255 (0.176–0.330)	0.054
	ML	0.988 (0.980–0.994)	0.899 (0.801–0.971)	0.278 (0.071–0.500)	0.998 (0.996–1.000)	0.714 (0.333–1.000)	0.400 (0.118–0.629)	
Non-gonococcal Urethritis	BN	0.854 (0.845–0.862)	0.903 (0.899–0.912)	0.819 (0.800–0.839)	0.864 (0.854–0.874)	0.652 (0.631–0.672)	0.726 (0.710–0.742)	0.929
	ML	0.864 (0.844–0.886)	0.902 (0.880–0.922)	0.793 (0.743–0.836)	0.886 (0.864–0.907)	0.683 (0.633–0.736)	0.734 (0.696–0.770)	
Warts^#^	BN	0.863 (0.854–0.871)	0.843 (0.828–0.858)	0.630 (0.594–0.665)	0.894 (0.885–0.902)	0.441 (0.411–0.472)	0.519 (0.489–0.547)	0.033
	ML	0.882 (0.865–0.900)	0.878 (0.850–0.907)	0.653 (0.576–0.731)	0.912 (0.896–0.929)	0.497 (0.426–0.572)	0.565 (0.496–0.626)	
**Females**	**Model**	**Accuracy**	**AUC**	**Recall**	**specificity**	**Precision**	**F1**	
Bacterial Vaginosis	BN	0.816 (0.805–0.828)	0.778 (0.757–0.797)	0.588 (0.555–0.624)	0.861 (0.849–0.872)	0.450 (0.417–0.483)	0.510 (0.480–0.538)	0.563
	ML	0.826 (0.798–0.848)	0.792 (0.748–0.834)	0.575 (0.497–0.651)	0.874 (0.849–0.895)	0.468 (0.398–0.536)	0.516 (0.447–0.577)	
Candidiasis	BN	0.713 (0.700–0.726)	0.771 (0.754–0.790)	0.760 (0.727–0.791)	0.704 (0.689–0.718)	0.338 (0.315–0.360)	0.468 (0.443–0.492)	0.185
	ML	0.799 (0.773–0.826)	0.801 (0.759–0.840)	0.625 (0.543–0.702)	0.834 (0.806–0.861)	0.427 (0.359–0.495)	0.507 (0.439–0.571)	
Cystitis (UTI)	BN	0.939 (0.931–0.945)	0.867 (0.841–0.892)	0.419 (0.357–0.476)	0.973 (0.968–0.977)	0.509 (0.441–0.568)	0.460 (0.400–0.511)	0.434
	ML	0.937 (0.921–0.952)	0.843 (0.787–0.896)	0.426 (0.304–0.556)	0.971 (0.959–0.983)	0.489 (0.340–0.649)	0.455 (0.333–0.577)	
Herpes	BN	0.953 (0.947–0.959)	0.856 (0.823–0.886)	0.408 (0.335–0.480)	0.976 (0.971–0.981)	0.413 (0.341–0.486)	0.410 (0.342–0.474)	0.475
	ML	0.948 (0.931–0.963)	0.881 (0.811–0.933)	0.486 (0.308–0.645)	0.968 (0.955–0.980)	0.386 (0.245–0.526)	0.430 (0.289–0.556)	
Molluscum Contagiosum	BN	0.954 (0.948–0.960)	0.896 (0.850–0.936)	0.600 (0.468–0.724)	0.959 (0.953–0.964)	0.157 (0.110–0.205)	0.249 (0.179–0.313)	0.093
	ML	0.971 (0.960–0.982)	0.943 (0.906–0.974)	0.455 (0.167–0.750)	0.978 (0.967–0.987)	0.208 (0.056–0.400)	0.286 (0.083–0.478)	
PID	BN	0.873 (0.863–0.882)	0.781 (0.756–0.804)	0.395 (0.338–0.453)	0.906 (0.896–0.914)	0.224 (0.185–0.263)	0.286 (0.242–0.326)	0.402
	ML	0.678 (0.646–0.710)	0.752 (0.682–0.809)	0.821 (0.706–0.922)	0.668 (0.634–0.700)	0.145 (0.108–0.185)	0.247 (0.189–0.304)	
Warts	BN	0.953 (0.947–0.959)	0.845 (0.809–0.876)	0.414 (0.343–0.483)	0.977 (0.973–0.982)	0.454 (0.384–0.532)	0.433 (0.370–0.495)	0.681
	ML	0.952 (0.938–0.966)	0.861 (0.787–0.924)	0.474 (0.316–0.639)	0.974 (0.963–0.984)	0.450 (0.297–0.605)	0.462 (0.314–0.595)	

*BN* Bayesian Network Model, *ML* Machine Learning Model

^#^*p*-value less than 0.05 shows significant difference of AUC between machine learning and Bayesian models

*p*-values were calculated with Z-test, with standard errors obtained from the bootstrap confidence intervals

## Discussion

We used prospectively collected clinical dataset to determine if machine learning techniques were superior to Bayesian networks to predict a correct diagnosis for common STIs and anogenital conditions. Using Bayesian networks, we had previously found a reasonably good predictive value and had used this to develop a public web-based symptom checker (ispysti.org) that provided users with a numerical prediction of their likely diagnosis and a referral letter to a healthcare provider. In this study, we used the same database and 12 common STI conditions and analysed this with machine learning techniques. The machine learning models performed well at predicting the correct diagnosis for most male and female conditions (AUC 0.75–0.95) during model evaluation with unseen datasets, demonstrating reasonable generalizability except for UTI and cervicitis. We also identified key predictors that align closely with a correct diagnosis. Urethral discharge and other urinary symptoms were positive predictors for urethral gonorrhoea, NGU, and UTIs. Similarly, the appearance of skin lesions matching the presented images and their location were predictive of correct diagnosis of anogenital skin lesions. When compared with Bayesian models, machine learning models provided similar results except that machine learning models were better for male balanitis, molluscum contagiosum and genital warts (*p*-value < 0.05) and Bayesian models were better for male gonorrhoea, female PID and UTI (*p*-value > 0.05). We are not aware of other work using machine learning or Bayesian networks to predict the correct diagnosis.

Our study is the first study to use Bayesian networks and machine learning models to assess STI conditions in symptomatic individuals, using large, systematically designed datasets collected prospectively for the development of a STI symptom checker. While many studies have focused on predicting HIV and STI risks among asymptomatic individuals [19–22], only two previous studies have applied machine learning for checking STI symptoms. Our previous study [24] used text manually extracted data from clinical notes to distinguish STIs and non-STIs with the CatBoost model achieving a high AUC of 0.91. However, that study was limited by a small sample size (*n* = 1,315), lack of external validation, potential inconsistencies in clinical notes, and inability to comprehensively cover STIs and common anogenital conditions. In another study, Adeboye et al., [34] developed an AdaBoost model using self-reported data to distinguish STIs from non-STIs, achieving an AUC of 0.94. This study was also limited by a small sample size (*n* = 400, mostly women) from a school clinic and used only seven genitourinary symptoms as predictors. Both these studies relied on available Electronic Health Record (EHR) data, which may not fully capture all the predictors. In contrast, our current study used purposefully designed datasets for male and female conditions that mirror the thorough assessment process of sexual health specialists. This approach potentially offers more accurate and relevant predictors, enabling our model to provide a more comprehensive assessment and improve accuracy and clinical utility.

While our models performed well in assessing STI symptoms, we must carefully consider their capabilities, limitations, and potential impacts on both users and healthcare system. Our tool’s primary purpose is to provide personalised advice, raise symptom awareness, and promote health-seeking behaviours, not to replace physician consultations. Accurate diagnosis still requires a comprehensive assessment, including history taking, physical examination and laboratory tests and our online symptom checkers did not aim to replicate this. Our online symptoms checker is more comparable to telephone triage lines used in primary care (e.g. nurse on call) but are superior to internet search results [35]. Machine learning and Bayesian networks allow us to adjust sensitivity and specificity thresholds which may also influence their integration with the existing health care system. High sensitivity helps detect more cases but also might increase healthcare system burden if the specificity is low and individuals without STIs are directed to health services. Low specificity (resulting in false positive results) may cause unnecessary user anxiety. Conversely, lowering sensitivity could provide false reassurance to those with infections, especially for important diseases such as syphilis and PID, with both adverse individual and public health consequences.

We identified important insights into clients’ responses to questions about their anogenital skin symptoms that have not been previously documented. For example, less than half of those diagnosed with molluscum contagiosum reported having a lump, yet correctly selected an image of a lump (molluscum contagiosum) as the image that was the most similar to their condition. This was a feature across other terms, such as blisters and spots. This finding showed that user responses to terms that have a specific medical meaning could vary based on prior knowledge and understanding, even when simplified terms were used in questionnaires. It is likely that considerable research will be required if words alone, rather than images are used to collect data for these models. Our study suggests images are more important and less susceptible to misunderstanding about terms, especially for anogenital skin conditions.

There are three main ways to collect information from individuals about their symptoms. The first way is by asking questions using descriptive terms (e.g., a lump or spot? ). The second option is to ask individuals if their lesions resemble the images shown in questionnaires, allowing for a visual comparison. Lastly, individuals can also upload an image of their skin condition, which can be analysed using AI technology. In our symptom checker, we implemented the first two approaches. Our previous studies showed that CNN models could identify STI lesions in uploaded images with accuracies ranging from 59.2 to 82.1% [36, 37]. Combining image analysis with clinical text further improved model performance [38]. In our future work, we will compare the symptom checker’s performance with and without user-uploaded lesion images, investigating the potential benefits of incorporating CNN models in symptom checking.

The AUC scores from machine learning and Bayesian models were not significantly different for most conditions except for balanitis, molluscum contagiosum and genital warts. Both approaches have their strengths and limitations. While machine learning excels at handling complex, large-scale data, healthcare providers have expressed concerns about its ‘black box’ nature, which provides predictions without clear explanations. In contrast, Bayesian models offer greater interpretability to better understand the relationship between user responses (symptoms) and STI diagnosis. However, recent developments in machine learning interpretability, such as SHAP analysis, now offer valuable insights into symptom-diagnosis relationships (Figure S3). Moreover, machine learning offers greater flexibility for integration with other models, such as image recognition algorithms, due to the wide availability of open-source Python packages in our STI symptom checker. The choice between machine learning and Bayesian models depends on the specific health problem, data size and complexity, interpretability requirements, potential for model integration, and available expertise.

Despite being a large prospectively collected clinical study our study had a number of limitations. First, it was conducted at a single centre and may not be generalisable for information collected elsewhere. This may particularly influence the responses to some questions if these were systematically different in other populations. MSHC is, however, Australia’s largest sexual health centre, which provides services to a population of more than 5 million and is likely to have a similar client profile to other sexual health services in Australia. Second, we excluded the transgender and gender-diverse population, as well as conditions like chlamydia, secondary syphilis, proctitis, mpox, trichomoniasis and female gonococcal infection in our current models. Because we did not collect a sufficient number of positive cases necessary for model training. Moreover, due to low positivity rates of male UTI and female cervicitis in our dataset, we excluded these models in our symptom checker as their performance was insufficient during the evaluation. We collected information on anorectal symptoms (discharge, tenesmus, and altered bowel habits) during the later stages of the iSpySTI project, as these symptoms are commonly reported among MSM. However, we did not incorporate them in the current study. Including these conditions in future versions would make the tool more comprehensive. MSHC plans to improve the iSpySTI based on recent findings from the qualitative study [18]. Third, we used self-reported questionnaire data to train the machine learning models and it may introduce bias depending on users’ native language, literacy, understanding of the questions, recall and interpretation of their conditions. Fourth, we collected data as part of a study and only those who agreed to participate were included in the study. It is therefore possible that those who participated in the study were systematically different from those who did not. While we did not formally calculate the participation rate it was likely to be about 10–20% given the number of individuals with symptoms who came through the clinic. Finally, the comparison of performance between machine learning and Bayesian models was limited by the use of different testing datasets, although both were unseen by their respective models. We addressed this limitation through bootstrap resampling.

## Conclusion

In summary, we developed a machine learning-based symptom checker for 12 common STIs and anogenital conditions in both men and women, using demographics, sexual behaviours, and presenting symptoms. Machine learning significantly outperforms Bayesian models only in male balanitis, molluscum contagiosum and genital warts, and the choice between them depends on specific implementation requirements.

## Supplementary Information

Supplementary Material 1. Table 1. Demographic characteristics and presenting symptoms by diagnosis in male clients. Table 2. Demographic characteristics and presenting symptoms by diagnosis in female clients. Table 3. Performance comparison between machine learning and Bayesian Network models. Figure 1. Workflows for model development, evaluation and predictions. Figure 2. The AUC for the best performing machine learning models throughout validation, testing and external testing across different diseases. Table S1. Predictor variables and outcome variables. Table S2. Performance difference across validation, testing and external validation in male clients. Table S3. Performance difference across validation, testing and external validation in female clients. Figure S1. Performance comparison of machine learning models during training and Validation in male clients. Figure S2. Performance comparison of machine learning models during training and validation in female clients. Figure S3. Interpretation of machine learning models for conditions in males. Figure S4. Precision-Recall and Sensitivity-Specificity curves for Bayesian and Machine learning models for each disease condition.

## Data Availability

The dataset used in this study is not publicly available due to privacy and consent limitations.
