# Supplementary material for: Accuracy of symptom checker for the diagnosis of sexually transmitted infections using machine learning and Bayesian network algorithms
Source: BMC Infect Dis. 2024 Dec 18;24:1408. doi: 10.1186/s12879-024-10285-4 (PMC11654068; doi:10.1186/s12879-024-10285-4)

Table S1. Predictor variables and outcome variables

| **Predictor variables** | **Male** | **Female** |
| --- | --- | --- |
| **Demographics** | | |
| Age (Years) (Median, IQR) | ✓ | ✓ |
| Country of birth | ✓ | ✓ |
| Duration since arrival in Australia | ✓ | ✓ |
| **Sexual behaviours** | | |
| Had unprotected anal sex after your last HIV test | ✓ | ✗ |
| Sex oversea | ✓ | ✓ |
| Ever MSM | ✓ | ✗ |
| Had sex with men in the past 12 months | ✓ | ✓ |
| Number of men you had sex with | ✓ | ✓ |
| Had sex with women in the past 12 months | ✓ | ✓ |
| Number of women you had sex with | ✓ | ✓ |
| Condom use with male partner | ✓ | ✓ |
| Condom use with female partner | ✓ | ✓ |
| **Anogenital skin symptoms** | | |
| Itch | ✓ | ✓ |
| Rash | ✓ | ✓ |
| Lumps | ✓ | ✓ |
| Spots | ✓ | ✓ |
| Blisters and/ sores | ✓ | ✓ |
| None of skin symptoms | ✓ | ✓ |
| Site of skin symptoms | ✓ | ✓ |
| Pain level of blisters and/sores | ✓ | ✓ |
| 2 Pictures of genital warts | ✓ | ✓ |
| Picture of balanitis | ✓ | ✗ |
| Picture of molluscum contagiosum | ✓ | ✓ |
| Picture of syphilis | ✓ | ✗ |
| 2 Pictures of herpes simplex | ✓ | ✓ |
| Picture of Tinea cruris | ✓ | ✓ |
| Picture of pearly penile papule | ✓ | ✗ |
| Picture of Fordyce spot | ✓ | ✗ |
| Picture of Vestibular glands | ✗ | ✓ |
| **Urethral symptoms** | | |
| Pain | ✓ | ✗ |
| Pain scale | ✓ | ✗ |
| Itch | ✓ | ✗ |
| Unusual discharge | ✓ | ✗ |
| None of urethral symptoms | ✓ | ✗ |
| Type of discharge | ✓ | ✗ |
| **Urinary symptoms** | | |
| Burning | ✓ | ✓ |
| Urge to pass urine | ✓ | ✓ |
| Blood | ✓ | ✓ |
| Associated with fever | ✓ | ✓ |
| No urinary symptom | ✓ | ✓ |
| Duration of urinary symptoms | ✓ | ✓ |
| **Vaginal discharge symptoms** | | |
| Unusual discharge | ✗ | ✓ |
| Unusual smell | ✗ | ✓ |
| Type of discharge | ✗ | ✓ |
| Associated with itch | ✗ | ✓ |
| **Pelvic pain** | | |
| Pain in Pelvic or genital area | ✗ | ✓ |
| Location | ✗ | ✓ |
| Pain scale | ✗ | ✓ |
| **Symptoms of Vaginal bleeding or dyspareunia** | | |
| Bleeding during sex | ✗ | ✓ |
| Spotting between period | ✗ | ✓ |
| Pain during sex | ✗ | ✓ |
| None of above symptoms | ✗ | ✓ |
| Location of pain during sex | ✗ | ✓ |
| **Outcome variables** |  |  |
| Diagnoses | Balanitis; Gonorrhoea; Herpes; Molluscum Contagiosum; Syphilis; NGU; UTI; Warts | Bacterial Vaginosis; Candidiasis; Cervicitis; Cystitis; Herpes; Molluscum Contagiosum; PID, Warts |


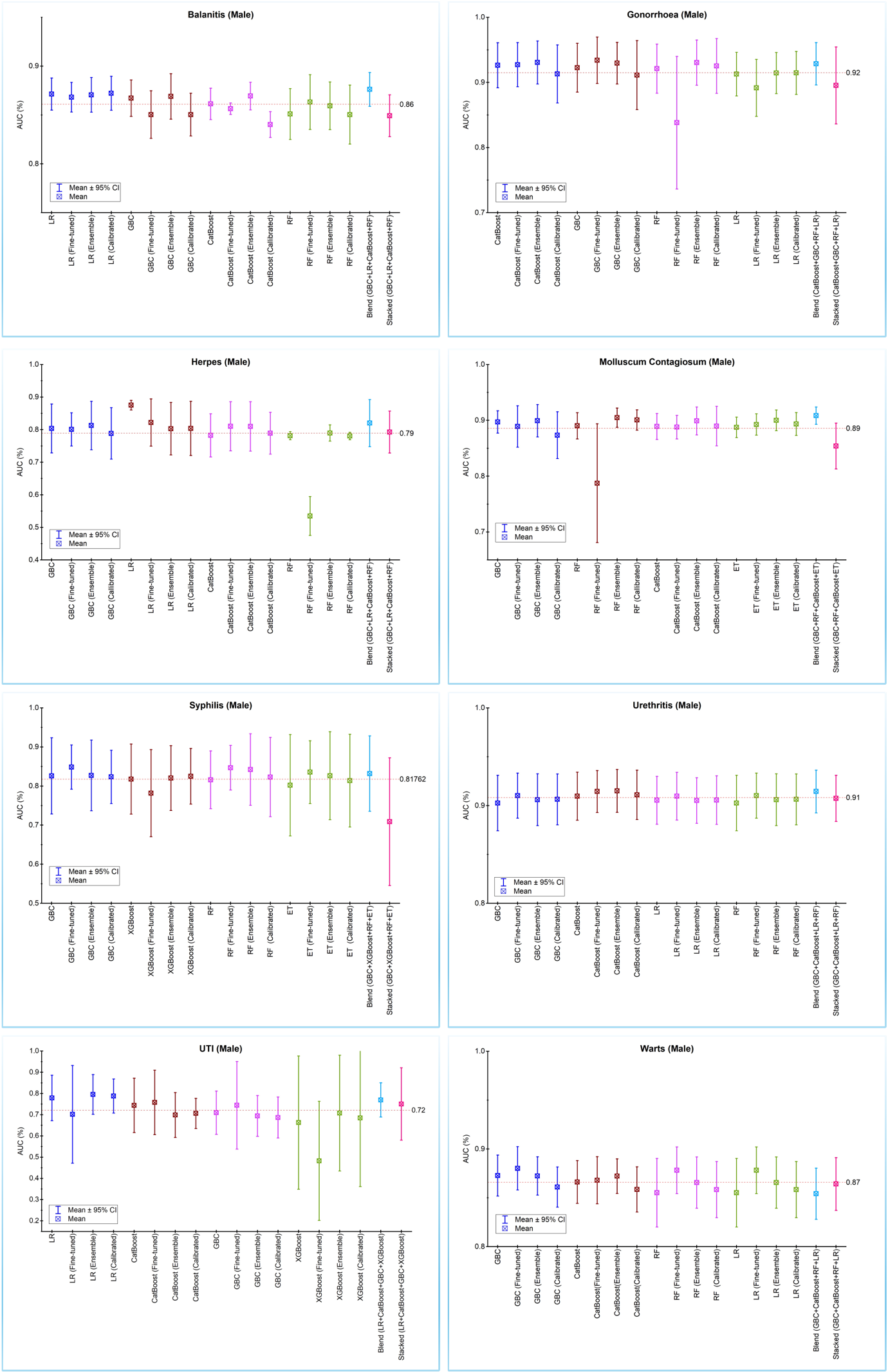


Figure S1. Performance comparison of machine learning models during training and Validation in male clients


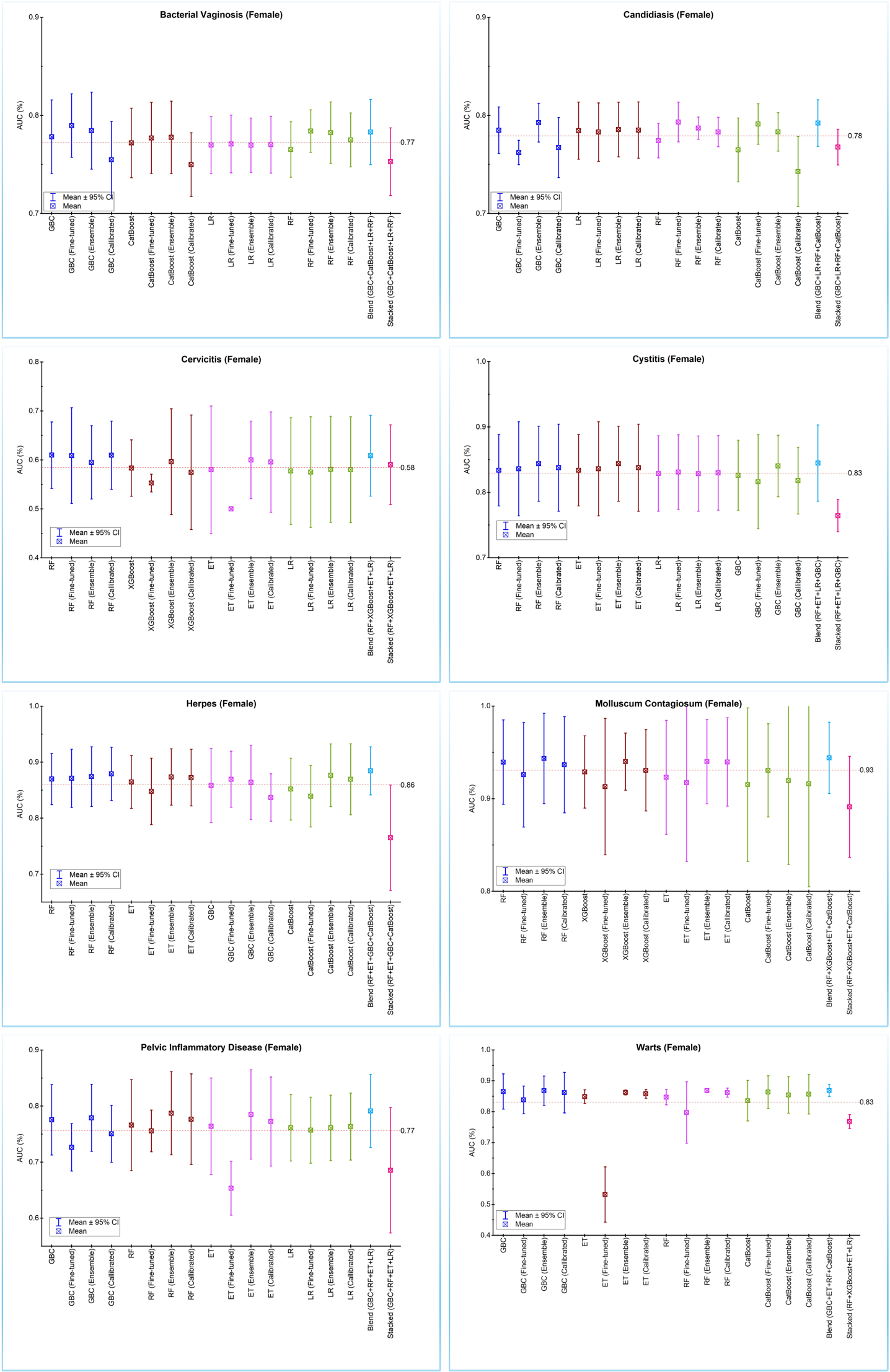


Figure S2. Performance comparison of machine learning models during training and validation in female client

Table S2. Performance difference across validation, testing and external validation in male clients

1. **Syphilis (Male)**

| Anova: Two-Factor Without Replication | |  |  |  |  |  |  |
| --- | --- | --- | --- | --- | --- | --- | --- |
| *SUMMARY* | *Validation AUC* | *Testing AUC* | *Ext Testing AUC* | *Count* | *Sum* | *Average* | *Variance* |
| GBC | 0.826 | 0.901 | 0.840 | 3 | 2.568 | 0.856 | 0.002 |
| fine-tuned GBC | 0.849 | 0.901 | 0.840 | 3 | 2.590 | 0.863 | 0.001 |
| Ensembled GBC | 0.827 | 0.903 | 0.865 | 3 | 2.595 | 0.865 | 0.001 |
| Calibrated GBC | 0.824 | 0.880 | 0.842 | 3 | 2.545 | 0.848 | 0.001 |
| XGBoost | 0.818 | 0.883 | 0.817 | 3 | 2.517 | 0.839 | 0.001 |
| fine-tuned XGBoost | 0.782 | 0.883 | 0.817 | 3 | 2.481 | 0.827 | 0.003 |
| Ensembled XGBoost | 0.820 | 0.913 | 0.855 | 3 | 2.588 | 0.863 | 0.002 |
| Calibrated XGBoost | 0.825 | 0.912 | 0.829 | 3 | 2.566 | 0.855 | 0.002 |
| RF | 0.816 | 0.889 | 0.925 | 3 | 2.631 | 0.877 | 0.003 |
| fine-tuned RF | 0.847 | 0.889 | 0.925 | 3 | 2.662 | 0.887 | 0.002 |
| **Ensembled RF** | **0.842** | **0.920** | **0.916** | **3** | **2.678** | **0.893** | **0.002** |
| Calibrated RF | 0.823 | 0.911 | 0.833 | 3 | 2.568 | 0.856 | 0.002 |
| ET | 0.802 | 0.876 | 0.849 | 3 | 2.527 | 0.842 | 0.001 |
| fine-tuned ET | 0.836 | 0.876 | 0.849 | 3 | 2.560 | 0.853 | 0.000 |
| Ensembled ET | 0.827 | 0.916 | 0.917 | 3 | 2.660 | 0.887 | 0.003 |
| Calibrated ET | 0.814 | 0.900 | 0.860 | 3 | 2.574 | 0.858 | 0.002 |
| Blended models | 0.832 | 0.918 | 0.895 | 3 | 2.645 | 0.882 | 0.002 |
| Stacked model | 0.709 | 0.786 | 0.850 | 3 | 2.344 | 0.781 | 0.005 |
| Validation AUC |  |  |  | 18 | 14.717 | **0.818** | 0.001 |
| Testing AUC |  |  |  | 18 | 16.057 | **0.892** | 0.001 |
| Ext Testing AUC |  |  |  | 18 | 15.524 | 0.862 | 0.001 |

| ANOVA |  |  |  |  |  |  |
| --- | --- | --- | --- | --- | --- | --- |
| ***Source of Variation*** | ***SS*** | ***df*** | ***MS*** | ***F*** | ***P-value*** | ***F crit*** |
| Rows | 0.03431368 | 17 | 0.002018451 | 3.26567067 | **0.00161495** | 1.93320683 |
| Columns | 0.05056868 | 2 | 0.025284341 | 40.9077612 | **8.9352E-10** | 3.27589799 |
| Error | 0.02101478 | 34 | 0.000618082 |  |  |  |
| Total | 0.10589714 | 53 |  |  |  |  |

1. **Warts (Male)**

| Anova: Two-Factor Without Replication | |  |  |  |  |  |  |
| --- | --- | --- | --- | --- | --- | --- | --- |
| *SUMMARY* | *Validation AUC* | *Testing AUC* | *Ext Testing AUC* | *Count* | *Sum* | *Average* | *Variance* |
| GBC | 0.873 | 0.877 | 0.866 | 3 | 2.616 | 0.872 | 0.000 |
| fine-tuned GBC | 0.880 | 0.877 | 0.866 | 3 | 2.624 | 0.875 | 0.000 |
| Ensembled GBC | 0.872 | 0.882 | 0.873 | 3 | 2.627 | 0.876 | 0.000 |
| Calibrated GBC | 0.861 | 0.872 | 0.862 | 3 | 2.595 | 0.865 | 0.000 |
| CatBoost | 0.866 | 0.870 | 0.864 | 3 | 2.599 | 0.866 | 0.000 |
| fine-tuned CatBoost | 0.868 | 0.877 | 0.870 | 3 | 2.615 | 0.872 | 0.000 |
| Ensembled CatBoost | 0.872 | 0.873 | 0.868 | 3 | 2.614 | 0.871 | 0.000 |
| Calibrated CatBoost | 0.859 | 0.860 | 0.856 | 3 | 2.574 | 0.858 | 0.000 |
| RF | 0.855 | 0.858 | 0.859 | 3 | 2.573 | 0.858 | 0.000 |
| fine-tuned RF | 0.878 | 0.858 | 0.859 | 3 | 2.596 | 0.865 | 0.000 |
| Ensembled RF | 0.866 | 0.869 | 0.869 | 3 | 2.604 | 0.868 | 0.000 |
| Calibrated RF | 0.858 | 0.860 | 0.857 | 3 | 2.575 | 0.858 | 0.000 |
| LR | 0.855 | 0.880 | 0.847 | 3 | 2.582 | 0.861 | 0.000 |
| fine-tuned LR | 0.864 | 0.884 | 0.860 | 3 | 2.608 | 0.869 | 0.000 |
| Ensembled LR | 0.855 | 0.880 | 0.849 | 3 | 2.584 | 0.861 | 0.000 |
| Calibrated LR | 0.855 | 0.880 | 0.848 | 3 | 2.583 | 0.861 | 0.000 |
| **Blended models** | **0.875** | **0.887** | **0.868** | **3** | **2.630** | **0.877** | **0.000** |
| Stacked model | 0.853 | 0.860 | 0.843 | 3 | 2.556 | 0.852 | 0.000 |
| Validation AUC |  |  |  | 18 | 15.565 | 0.865 | 0.000 |
| Testing AUC |  |  |  | 18 | 15.704 | 0.872 | 0.000 |
| Ext Testing AUC |  |  |  | 18 | 15.485 | 0.860 | 0.000 |

| ANOVA |  |  |  |  |  |  |
| --- | --- | --- | --- | --- | --- | --- |
| *Source of Variation* | *SS* | *df* | *MS* | *F* | *P-value* | *F crit* |
| Rows | 0.00254938 | 17 | 0.000149964 | 3.11578989 | **0.00233696** | 1.93320683 |
| Columns | 0.00136281 | 2 | 0.000681405 | 14.1575245 | **3.3656E-05** | 3.27589799 |
| Error | 0.00163643 | 34 | 4.81302E-05 |  |  |  |
| Total | 0.00554862 | 53 |  |  |  |  |

1. **Urinary Tract Infection (Male)**

| Anova: Two-Factor Without Replication | |  |  |  |  |  |  |
| --- | --- | --- | --- | --- | --- | --- | --- |
| *SUMMARY* | *Validation AUC* | *Testing AUC* | *Ext Testing AUC* | *Count* | *Sum* | *Average* | *Variance* |
| LR | 0.779 | 0.238 | 0.970 | 3 | 1.986 | 0.662 | 0.144 |
| fine-tuned LR | 0.702 | 0.227 | 0.896 | 3 | 1.825 | 0.608 | 0.118 |
| Ensembled LR | 0.796 | 0.220 | 0.973 | 3 | 1.989 | 0.663 | 0.155 |
| Calibrated LR | 0.788 | 0.243 | 0.975 | 3 | 2.005 | 0.668 | 0.145 |
| CatBoost | 0.744 | 0.000 | 0.701 | 3 | 1.445 | 0.482 | 0.174 |
| fine-tuned CatBoost | 0.758 | 0.296 | 0.701 | 3 | 1.754 | 0.585 | 0.064 |
| Ensembled CatBoost | 0.699 | 0.345 | 0.765 | 3 | 1.809 | 0.603 | 0.051 |
| Calibrated CatBoost | 0.706 | 0.434 | 0.480 | 3 | 1.620 | 0.540 | 0.021 |
| GBC | 0.710 | 0.718 | 0.973 | 3 | 2.400 | 0.800 | 0.022 |
| fine-tuned GBC | 0.745 | 0.718 | 0.973 | 3 | 2.435 | 0.812 | 0.020 |
| Ensembled GBC | 0.694 | 0.642 | 0.879 | 3 | 2.216 | 0.739 | 0.016 |
| Calibrated GBC | 0.687 | 0.573 | 0.873 | 3 | 2.133 | 0.711 | 0.023 |
| XGBoost | 0.663 | 0.818 | 0.939 | 3 | 2.420 | 0.807 | 0.019 |
| fine-tuned XGBoost | 0.483 | 0.818 | 0.939 | 3 | 2.240 | 0.747 | 0.056 |
| Ensembled XGBoost | 0.707 | 0.790 | 0.936 | 3 | 2.433 | 0.811 | 0.013 |
| Calibrated XGBoost | 0.685 | 0.777 | 0.915 | 3 | 2.377 | 0.792 | 0.013 |
| Blended models | 0.769 | 0.660 | 0.970 | 3 | 2.399 | 0.800 | 0.025 |
| Stacked model | 0.751 | 0.194 | 0.992 | 3 | 1.936 | 0.645 | 0.168 |
| Validation AUC |  |  |  | 18 | 12.862 | 0.715 | 0.005 |
| Testing AUC |  |  |  | 18 | 8.709 | 0.484 | 0.071 |
| Ext Testing AUC |  |  |  | 18 | 15.851 | 0.881 | 0.018 |

| ANOVA |  |  |  |  |  |  |
| --- | --- | --- | --- | --- | --- | --- |
| *Source of Variation* | *SS* | *df* | *MS* | *F* | *P-value* | *F crit* |
| Rows | 0.53654302 | 17 | 0.031561354 | 1.0070347 | 0.47469306 | 1.93320683 |
| Columns | 1.42963444 | 2 | 0.714817222 | 22.807822 | 5.226E-07 | 3.27589799 |
| Error | 1.06558994 | 34 | 0.03134088 |  |  |  |
| Total | 3.0317674 | 53 |  |  |  |  |

1. **Non-Gonococcal Urethritis (Male)**

| Anova: Two-Factor Without Replication | |  |  |  |  |  |  |
| --- | --- | --- | --- | --- | --- | --- | --- |
| *SUMMARY* | *Validation AUC* | *Testing AUC* | *Ext Testing AUC* | *Count* | *Sum* | *Average* | *Variance* |
| GBC | 0.903 | 0.900 | 0.912 | 3 | 2.715 | 0.905 | 0.000 |
| fine-tuned GBC | 0.910 | 0.900 | 0.912 | 3 | 2.722 | 0.907 | 0.000 |
| Ensembled GBC | 0.906 | 0.909 | 0.917 | 3 | 2.732 | 0.911 | 0.000 |
| Calibrated GBC | 0.906 | 0.907 | 0.915 | 3 | 2.728 | 0.909 | 0.000 |
| CatBoost | 0.910 | 0.914 | 0.906 | 3 | 2.730 | 0.910 | 0.000 |
| fine-tuned CatBoost | 0.914 | 0.914 | 0.906 | 3 | 2.735 | 0.912 | 0.000 |
| Ensembled CatBoost | 0.915 | 0.919 | 0.915 | 3 | 2.749 | 0.916 | 0.000 |
| Calibrated CatBoost | 0.911 | 0.919 | 0.911 | 3 | 2.741 | 0.914 | 0.000 |
| LR | 0.905 | 0.917 | 0.910 | 3 | 2.732 | 0.911 | 0.000 |
| fine-tuned LR | 0.910 | 0.920 | 0.917 | 3 | 2.747 | 0.916 | 0.000 |
| Ensembled LR | 0.905 | 0.916 | 0.909 | 3 | 2.730 | 0.910 | 0.000 |
| Calibrated LR | 0.906 | 0.918 | 0.912 | 3 | 2.735 | 0.912 | 0.000 |
| RF | 0.903 | 0.900 | 0.912 | 3 | 2.715 | 0.905 | 0.000 |
| fine-tuned RF | 0.910 | 0.900 | 0.912 | 3 | 2.722 | 0.907 | 0.000 |
| Ensembled RF | 0.906 | 0.909 | 0.917 | 3 | 2.732 | 0.911 | 0.000 |
| Calibrated RF | 0.906 | 0.907 | 0.915 | 3 | 2.728 | 0.909 | 0.000 |
| **Blended models** | **0.914** | **0.921** | **0.923** | **3** | **2.758** | **0.919** | **0.000** |
| Stacked model | 0.907 | 0.919 | 0.918 | 3 | 2.744 | 0.915 | 0.000 |
| Validation AUC |  |  |  | 18 | 16.347 | 0.908 | 0.000 |
| Testing AUC |  |  |  | 18 | 16.410 | 0.912 | 0.000 |
| Ext Testing AUC |  |  |  | 18 | 16.437 | 0.913 | 0.000 |

| ANOVA |  |  |  |  |  |  |
| --- | --- | --- | --- | --- | --- | --- |
| *Source of Variation* | *SS* | *df* | *MS* | *F* | *P-value* | *F crit* |
| Rows | 0.00074192 | 17 | 4.36425E-05 | 1.82527609 | 0.06642423 | 1.93320683 |
| Columns | 0.00023627 | 2 | 0.000118134 | 4.94077464 | 0.01307219 | 3.27589799 |
| Error | 0.00081294 | 34 | 2.39101E-05 |  |  |  |
| Total | 0.00179113 | 53 |  |  |  |  |

1. **Gonorrhoea (Male)**

| Anova: Two-Factor Without Replication | |  |  |  |  |  |  |
| --- | --- | --- | --- | --- | --- | --- | --- |
| *SUMMARY* | *Validation AUC* | *Testing AUC* | *Ext Testing AUC* | *Count* | *Sum* | *Average* | *Variance* |
| CatBoost | 0.926 | 0.944 | 0.903 | 3 | 2.773 | 0.924 | 0.000 |
| fine-tuned CatBoost | 0.927 | 0.944 | 0.903 | 3 | 2.774 | 0.925 | 0.000 |
| **Ensembled CatBoost** | **0.931** | **0.948** | **0.913** | **3** | **2.791** | **0.930** | **0.000** |
| Calibrated CatBoost | 0.913 | 0.937 | 0.914 | 3 | 2.764 | 0.921 | 0.000 |
| GBC | 0.922 | 0.938 | 0.924 | 3 | 2.785 | 0.928 | 0.000 |
| fine-tuned GBC | 0.934 | 0.935 | 0.922 | 3 | 2.791 | 0.930 | 0.000 |
| Ensembled GBC | 0.929 | 0.930 | 0.928 | 3 | 2.788 | 0.929 | 0.000 |
| Calibrated GBC | 0.911 | 0.926 | 0.935 | 3 | 2.772 | 0.924 | 0.000 |
| RF | 0.921 | 0.912 | 0.896 | 3 | 2.729 | 0.910 | 0.000 |
| fine-tuned RF | 0.838 | 0.912 | 0.896 | 3 | 2.646 | 0.882 | 0.002 |
| Ensembled RF | 0.930 | 0.939 | 0.912 | 3 | 2.781 | 0.927 | 0.000 |
| Calibrated RF | 0.925 | 0.929 | 0.891 | 3 | 2.744 | 0.915 | 0.000 |
| LR | 0.912 | 0.950 | 0.880 | 3 | 2.742 | 0.914 | 0.001 |
| fine-tuned LR | 0.891 | 0.928 | 0.871 | 3 | 2.690 | 0.897 | 0.001 |
| Ensembled LR | 0.914 | 0.948 | 0.881 | 3 | 2.744 | 0.915 | 0.001 |
| Calibrated LR | 0.915 | 0.952 | 0.882 | 3 | 2.749 | 0.916 | 0.001 |
| Blended model | 0.928 | 0.950 | 0.906 | 3 | 2.784 | 0.928 | 0.000 |
| Stacked model | 0.895 | 0.946 | 0.859 | 3 | 2.701 | 0.900 | 0.002 |
| Validation AUC |  |  |  | 18 | 16.465 | 0.915 | 0.001 |
| Testing AUC |  |  |  | 18 | 16.868 | 0.937 | 0.000 |
| Ext Testing AUC |  |  |  | 18 | 16.217 | 0.901 | 0.000 |

| ANOVA |  |  |  |  |  |  |
| --- | --- | --- | --- | --- | --- | --- |
| *Source of Variation* | *SS* | *df* | *MS* | *F* | *P-value* | *F crit* |
| Rows | 0.00906796 | 17 | 0.000533409 | 1.92300409 | **0.05136377** | 1.93320683 |
| Columns | 0.01198537 | 2 | 0.005992683 | 21.6043343 | **8.8068E-07** | 3.27589799 |
| Error | 0.00943103 | 34 | 0.000277383 |  |  |  |
| Total | 0.03048436 | 53 |  |  |  |  |

1. **Herpes (Male)**

| Anova: Two-Factor Without Replication | |  |  |  |  |  |  |
| --- | --- | --- | --- | --- | --- | --- | --- |
| *SUMMARY* | *Validation AUC* | *Testing AUC* | *Ext Testing AUC* | *Count* | *Sum* | *Average* | *Variance* |
| GBC | 0.804 | 0.811 | 0.810 | 3 | 2.425 | 0.808 | 0.000 |
| fine-tuned GBC | 0.801 | 0.811 | 0.810 | 3 | 2.422 | 0.807 | 0.000 |
| Ensembled GBC | 0.813 | 0.811 | 0.805 | 3 | 2.429 | 0.810 | 0.000 |
| Calibrated GBC | 0.788 | 0.787 | 0.794 | 3 | 2.369 | 0.790 | 0.000 |
| LR | 0.802 | 0.776 | 0.807 | 3 | 2.386 | 0.795 | 0.000 |
| fine-tuned LR | 0.822 | 0.810 | 0.808 | 3 | 2.439 | 0.813 | 0.000 |
| Ensembled LR | 0.803 | 0.780 | 0.806 | 3 | 2.388 | 0.796 | 0.000 |
| Calibrated LR | 0.804 | 0.780 | 0.807 | 3 | 2.391 | 0.797 | 0.000 |
| CatBoost | 0.783 | 0.737 | 0.787 | 3 | 2.306 | 0.769 | 0.001 |
| fine-tuned CatBoost | 0.810 | 0.793 | 0.799 | 3 | 2.402 | 0.801 | 0.000 |
| Ensembled CatBoost | 0.810 | 0.773 | 0.799 | 3 | 2.382 | 0.794 | 0.000 |
| Calibrated CatBoost | 0.789 | 0.767 | 0.783 | 3 | 2.339 | 0.780 | 0.000 |
| RF | 0.781 | 0.824 | 0.802 | 3 | 2.407 | 0.802 | 0.000 |
| fine-tuned RF | 0.535 | 0.824 | 0.802 | 3 | 2.161 | 0.720 | 0.026 |
| Ensembled RF | 0.790 | 0.832 | 0.808 | 3 | 2.430 | 0.810 | 0.000 |
| Calibrated RF | 0.781 | 0.824 | 0.790 | 3 | 2.394 | 0.798 | 0.001 |
| **Blended model** | **0.820** | **0.819** | **0.816** | **3** | **2.455** | **0.818** | **0.000** |
| Stacked model | 0.793 | 0.785 | 0.806 | 3 | 2.383 | 0.794 | 0.000 |
| Validation AUC |  |  |  | 18 | 14.126 | 0.785 | 0.004 |
| Testing AUC |  |  |  | 18 | 14.344 | 0.797 | 0.001 |
| Ext Testing AUC |  |  |  | 18 | 14.438 | 0.802 | 0.000 |

| ANOVA |  |  |  |  |  |  |
| --- | --- | --- | --- | --- | --- | --- |
| *Source of Variation* | *SS* | *df* | *MS* | *F* | *P-value* | *F crit* |
| Rows | 0.02482672 | 17 | 0.001460395 | 0.88109626 | **0.59836963** | 1.93320683 |
| Columns | 0.0028439 | 2 | 0.001421948 | 0.85789991 | **0.43302715** | 3.27589799 |
| Error | 0.05635415 | 34 | 0.001657475 |  |  |  |
| Total | 0.08402476 | 53 |  |  |  |  |

1. **Balanitis (Male)**

| Anova: Two-Factor Without Replication | |  |  |  |  |  |  |
| --- | --- | --- | --- | --- | --- | --- | --- |
| *SUMMARY* | *Validation AUC* | *Testing AUC* | *Ext Testing AUC* | *Count* | *Sum* | *Average* | *Variance* |
| LR | 0.872 | 0.890 | 0.864 | 3 | 2.625 | 0.875 | 0.000 |
| fine-tuned LR | 0.868 | 0.890 | 0.862 | 3 | 2.620 | 0.873 | 0.000 |
| Ensembled LR | 0.871 | 0.891 | 0.864 | 3 | 2.626 | 0.875 | 0.000 |
| Calibrated LR | 0.872 | 0.890 | 0.864 | 3 | 2.627 | 0.876 | 0.000 |
| GBC | 0.867 | 0.883 | 0.884 | 3 | 2.634 | 0.878 | 0.000 |
| fine-tuned GBC | 0.850 | 0.883 | 0.884 | 3 | 2.617 | 0.872 | 0.000 |
| Ensembled GBC | 0.869 | 0.889 | 0.893 | 3 | 2.650 | 0.883 | 0.000 |
| Calibrated GBC | 0.850 | 0.878 | 0.862 | 3 | 2.591 | 0.864 | 0.000 |
| CatBoost | 0.861 | 0.887 | 0.903 | 3 | 2.651 | 0.884 | 0.000 |
| fine-tuned CatBoost | 0.856 | 0.887 | 0.903 | 3 | 2.646 | 0.882 | 0.001 |
| Ensembled CatBoost | 0.869 | 0.893 | 0.886 | 3 | 2.648 | 0.883 | 0.000 |
| Calibrated CatBoost | 0.840 | 0.853 | 0.804 | 3 | 2.498 | 0.833 | 0.001 |
| RF | 0.851 | 0.852 | 0.882 | 3 | 2.584 | 0.861 | 0.000 |
| fine-tuned RF | 0.863 | 0.852 | 0.882 | 3 | 2.596 | 0.865 | 0.000 |
| Ensembled RF | 0.859 | 0.860 | 0.901 | 3 | 2.620 | 0.873 | 0.001 |
| Calibrated RF | 0.851 | 0.842 | 0.905 | 3 | 2.597 | 0.866 | 0.001 |
| **Blended model** | **0.876** | **0.891** | **0.884** | **3** | **2.651** | **0.884** | **0.000** |
| Stacked model | 0.849 | 0.853 | 0.861 | 3 | 2.564 | 0.855 | 0.000 |
| Validation AUC |  |  |  | 18 | 15.496 | 0.861 | 0.000 |
| Testing AUC |  |  |  | 18 | 15.762 | 0.876 | 0.000 |
| Ext Testing AUC |  |  |  | 18 | 15.786 | 0.877 | 0.001 |

| ANOVA |  |  |  |  |  |  |
| --- | --- | --- | --- | --- | --- | --- |
| *Source of Variation* | *SS* | *df* | *MS* | *F* | *P-value* | *F crit* |
| Rows | 0.00840321 | 17 | 0.000494306 | 1.94789101 | **0.04809974** | 1.93320683 |
| Columns | 0.00288949 | 2 | 0.001444746 | 5.69324941 | **0.00736865** | 3.27589799 |
| Error | 0.008628 | 34 | 0.000253765 |  |  |  |
| Total | 0.0199207 | 53 |  |  |  |  |

1. **Molluscum Contagiosum (Male)**

| Anova: Two-Factor Without Replication | |  |  |  |  |  |  |
| --- | --- | --- | --- | --- | --- | --- | --- |
| *SUMMARY* | *Validation AUC* | *Testing AUC* | *Ext Testing AUC* | *Count* | *Sum* | *Average* | *Variance* |
| GBC | 0.897 | 0.898 | 0.783 | 3 | 2.578 | 0.859 | 0.004 |
| fine-tuned GBC | 0.889 | 0.898 | 0.783 | 3 | 2.571 | 0.857 | 0.004 |
| Ensembled GBC | 0.899 | 0.895 | 0.764 | 3 | 2.558 | 0.853 | 0.006 |
| Calibrated GBC | 0.873 | 0.896 | 0.750 | 3 | 2.519 | 0.840 | 0.006 |
| RF | 0.890 | 0.862 | 0.743 | 3 | 2.495 | 0.832 | 0.006 |
| fine-tuned RF | 0.787 | 0.862 | 0.743 | 3 | 2.392 | 0.797 | 0.004 |
| Ensembled RF | 0.905 | 0.866 | 0.763 | 3 | 2.534 | 0.845 | 0.005 |
| Calibrated RF | 0.901 | 0.869 | 0.750 | 3 | 2.520 | 0.840 | 0.006 |
| CatBoost | 0.889 | 0.910 | 0.794 | 3 | 2.592 | 0.864 | 0.004 |
| fine-tuned CatBoost | 0.888 | 0.910 | 0.794 | 3 | 2.591 | 0.864 | 0.004 |
| **Ensembled CatBoost** | **0.899** | **0.914** | **0.813** | **3** | **2.626** | **0.875** | **0.003** |
| Calibrated CatBoost | 0.889 | 0.906 | 0.796 | 3 | 2.592 | 0.864 | 0.004 |
| ET | 0.887 | 0.827 | 0.750 | 3 | 2.464 | 0.821 | 0.005 |
| fine-tuned ET | 0.893 | 0.827 | 0.750 | 3 | 2.470 | 0.823 | 0.005 |
| Ensembled ET | 0.900 | 0.868 | 0.724 | 3 | 2.492 | 0.831 | 0.009 |
| Calibrated ET | 0.893 | 0.850 | 0.756 | 3 | 2.499 | 0.833 | 0.005 |
| Blended model | 0.909 | 0.886 | 0.489 | 3 | 2.284 | 0.761 | 0.056 |
| Stacked model | 0.854 | 0.806 | 0.607 | 3 | 2.266 | 0.755 | 0.017 |
| Validation AUC |  |  |  | 18 | 15.942 | 0.886 | 0.001 |
| Testing AUC |  |  |  | 18 | 15.749 | 0.875 | 0.001 |
| Ext Testing AUC |  |  |  | 18 | 13.352 | 0.742 | 0.006 |

| ANOVA |  |  |  |  |  |  |
| --- | --- | --- | --- | --- | --- | --- |
| *Source of Variation* | *SS* | *df* | *MS* | *F* | *P-value* | *F crit* |
| Rows | 0.05757681 | 17 | 0.003386871 | 1.5690491 | **0.12900801** | 1.93320683 |
| Columns | 0.23124965 | 2 | 0.115624826 | 53.5659708 | **3.1011E-11** | 3.27589799 |
| Error | 0.0733907 | 34 | 0.00215855 |  |  |  |
| Total | 0.36221716 | 53 |  |  |  |  |

Table S3. Performance difference across validation, testing and external validation in female clients

1. **Pelvic Inflammatory Disease (Female)**

| Anova: Two-Factor Without Replication | |  |  |  |  |  |  |
| --- | --- | --- | --- | --- | --- | --- | --- |
| *SUMMARY* | *Validation AUC* | *Testing AUC* | *Ext Testing AUC* | *Count* | *Sum* | *Average* | *Variance* |
| GBC | 0.775 | 0.797 | 0.772 | 3 | 2.344 | 0.781 | 0.000 |
| fine-tuned GBC | 0.726 | 0.797 | 0.772 | 3 | 2.295 | 0.765 | 0.001 |
| **Ensembled GBC** | **0.779** | **0.801** | **0.768** | **3** | **2.348** | **0.783** | **0.000** |
| Calibrated GBC | 0.751 | 0.793 | 0.758 | 3 | 2.301 | 0.767 | 0.001 |
| RF | 0.766 | 0.764 | 0.703 | 3 | 2.233 | 0.744 | 0.001 |
| fine-tuned RF | 0.756 | 0.764 | 0.703 | 3 | 2.222 | 0.741 | 0.001 |
| Ensembled RF | 0.787 | 0.780 | 0.728 | 3 | 2.295 | 0.765 | 0.001 |
| Calibrated RF | 0.777 | 0.768 | 0.689 | 3 | 2.234 | 0.745 | 0.002 |
| ET | 0.764 | 0.725 | 0.692 | 3 | 2.181 | 0.727 | 0.001 |
| fine-tuned ET | 0.653 | 0.725 | 0.692 | 3 | 2.070 | 0.690 | 0.001 |
| Ensembled ET | 0.785 | 0.767 | 0.729 | 3 | 2.280 | 0.760 | 0.001 |
| Calibrated ET | 0.773 | 0.734 | 0.742 | 3 | 2.248 | 0.749 | 0.000 |
| LR | 0.761 | 0.769 | 0.768 | 3 | 2.298 | 0.766 | 0.000 |
| fine-tuned LR | 0.757 | 0.769 | 0.768 | 3 | 2.294 | 0.765 | 0.000 |
| Ensembled LR | 0.761 | 0.768 | 0.770 | 3 | 2.299 | 0.766 | 0.000 |
| Calibrated LR | 0.763 | 0.771 | 0.770 | 3 | 2.305 | 0.768 | 0.000 |
| Blended model | 0.791 | 0.781 | 0.769 | 3 | 2.341 | 0.780 | 0.000 |
| Stacked model | 0.686 | 0.618 | 0.647 | 3 | 1.950 | 0.650 | 0.001 |
| Validation AUC |  |  |  | 18 | 13.611 | 0.756 | 0.001 |
| Testing AUC |  |  |  | 18 | 13.687 | 0.760 | 0.002 |
| Ext Testing AUC |  |  |  | 18 | 13.240 | 0.736 | 0.002 |

| ANOVA |  |  |  |  |  |  |
| --- | --- | --- | --- | --- | --- | --- |
| *Source of Variation* | *SS* | *df* | *MS* | *F* | *P-value* | *F crit* |
| Rows | 0.05739292 | 17 | 0.003376054 | 5.73667648 | **7.8563E-06** | 1.93320683 |
| Columns | 0.00635328 | 2 | 0.003176641 | 5.39782866 | **0.00920727** | 3.27589799 |
| Error | 0.02000912 | 34 | 0.000588504 |  |  |  |
| Total | 0.08375533 | 53 |  |  |  |  |

1. **Cervicitis (Female)**

| Anova: Two-Factor Without Replication | |  |  |  |  |  |  |
| --- | --- | --- | --- | --- | --- | --- | --- |
| *SUMMARY* | *Validation AUC* | *Testing AUC* | *Ext Testing AUC* | *Count* | *Sum* | *Average* | *Variance* |
| RF | 0.610 | 0.602 | 0.547 | 3 | 1.759 | 0.586 | 0.001 |
| fine-tuned RF | 0.609 | 0.602 | 0.547 | 3 | 1.758 | 0.586 | 0.001 |
| Ensembled RF | 0.595 | 0.673 | 0.543 | 3 | 1.811 | 0.604 | 0.004 |
| Calibrated RF | 0.610 | 0.659 | 0.575 | 3 | 1.843 | 0.614 | 0.002 |
| XGBoost | 0.583 | 0.599 | 0.551 | 3 | 1.734 | 0.578 | 0.001 |
| fine-tuned XGBoost | 0.553 | 0.599 | 0.551 | 3 | 1.704 | 0.568 | 0.001 |
| Ensembled XGBoost | 0.596 | 0.662 | 0.466 | 3 | 1.724 | 0.575 | 0.010 |
| Calibrated XGBoost | 0.575 | 0.650 | 0.454 | 3 | 1.678 | 0.559 | 0.010 |
| ET | 0.580 | 0.622 | 0.533 | 3 | 1.734 | 0.578 | 0.002 |
| fine-tuned ET | 0.500 | 0.500 | 0.500 | 3 | 1.500 | 0.500 | 0.000 |
| Ensembled ET | 0.600 | 0.680 | 0.544 | 3 | 1.823 | 0.608 | 0.005 |
| Calibrated ET | 0.596 | 0.616 | 0.581 | 3 | 1.792 | 0.597 | 0.000 |
| LR | 0.577 | 0.605 | 0.516 | 3 | 1.698 | 0.566 | 0.002 |
| fine-tuned LR | 0.575 | 0.613 | 0.511 | 3 | 1.699 | 0.566 | 0.003 |
| Ensembled LR | 0.581 | 0.610 | 0.509 | 3 | 1.700 | 0.567 | 0.003 |
| Calibrated LR | 0.580 | 0.606 | 0.513 | 3 | 1.699 | 0.566 | 0.002 |
| Blended model | 0.609 | 0.666 | 0.508 | 3 | 1.782 | 0.594 | 0.006 |
| **Stacked model** | **0.590** | **0.648** | **0.635** | **3** | **1.873** | **0.624** | **0.001** |
| Validation AUC |  |  |  | 18 | 10.518 | 0.584 | 0.001 |
| Testing AUC |  |  |  | 18 | 11.211 | 0.623 | 0.002 |
| Ext Testing AUC |  |  |  | 18 | 9.584 | 0.532 | 0.002 |

| ANOVA |  |  |  |  |  |  |
| --- | --- | --- | --- | --- | --- | --- |
| *Source of Variation* | *SS* | *df* | *MS* | *F* | *P-value* | *F crit* |
| Rows | 0.03844093 | 17 | 0.002261231 | 2.35261271 | **0.01652444** | 1.93320683 |
| Columns | 0.07413523 | 2 | 0.037067617 | 38.5656016 | **1.8027E-09** | 3.27589799 |
| Error | 0.03267935 | 34 | 0.000961157 |  |  |  |
| Total | 0.14525552 | 53 |  |  |  |  |

1. **Warts (Female)**

| Anova: Two-Factor Without Replication | |  |  |  |  |  |  |
| --- | --- | --- | --- | --- | --- | --- | --- |
| *SUMMARY* | *Validation AUC* | *Testing AUC* | *Ext Testing AUC* | *Count* | *Sum* | *Average* | *Variance* |
| GBC | 0.865 | 0.900 | 0.923 | 3 | 2.689 | 0.896 | 0.001 |
| fine-tuned GBC | 0.838 | 0.900 | 0.923 | 3 | 2.662 | 0.887 | 0.002 |
| Ensembled GBC | 0.868 | 0.904 | 0.904 | 3 | 2.676 | 0.892 | 0.000 |
| Calibrated GBC | 0.862 | 0.897 | 0.887 | 3 | 2.646 | 0.882 | 0.000 |
| ET | 0.849 | 0.895 | 0.897 | 3 | 2.640 | 0.880 | 0.001 |
| fine-tuned ET | 0.532 | 0.895 | 0.897 | 3 | 2.324 | 0.775 | 0.044 |
| Ensembled ET | 0.863 | 0.925 | 0.931 | 3 | 2.719 | 0.906 | 0.001 |
| Calibrated ET | 0.858 | 0.920 | 0.894 | 3 | 2.671 | 0.890 | 0.001 |
| RF | 0.847 | 0.938 | 0.928 | 3 | 2.714 | 0.905 | 0.002 |
| fine-tuned RF | 0.797 | 0.938 | 0.928 | 3 | 2.664 | 0.888 | 0.006 |
| Ensembled RF | 0.868 | 0.927 | 0.931 | 3 | 2.726 | 0.909 | 0.001 |
| Calibrated RF | 0.862 | 0.936 | 0.899 | 3 | 2.697 | 0.899 | 0.001 |
| CatBoost | 0.836 | 0.906 | 0.891 | 3 | 2.633 | 0.878 | 0.001 |
| fine-tuned CatBoost | 0.864 | 0.898 | 0.915 | 3 | 2.677 | 0.892 | 0.001 |
| Ensembled CatBoost | 0.854 | 0.902 | 0.925 | 3 | 2.681 | 0.894 | 0.001 |
| Calibrated CatBoost | 0.857 | 0.868 | 0.868 | 3 | 2.593 | 0.864 | 0.000 |
| **Blended model** | **0.869** | **0.929** | **0.932** | **3** | **2.730** | **0.910** | **0.001** |
| Stacked model | 0.768 | 0.808 | 0.905 | 3 | 2.480 | 0.827 | 0.005 |
| Validation AUC |  |  |  | 18 | 14.956 | 0.831 | 0.006 |
| Testing AUC |  |  |  | 18 | 16.288 | 0.905 | 0.001 |
| Ext Testing AUC |  |  |  | 18 | 16.377 | 0.910 | 0.000 |

| ANOVA |  |  |  |  |  |  |
| --- | --- | --- | --- | --- | --- | --- |
| *Source of Variation* | *SS* | *df* | *MS* | *F* | *P-value* | *F crit* |
| Rows | 0.05540206 | 17 | 0.003258945 | 1.51758042 | **0.14697239** | 1.93320683 |
| Columns | 0.07037222 | 2 | 0.03518611 | 16.3849818 | **1.0405E-05** | 3.27589799 |
| Error | 0.07301367 | 34 | 0.002147461 |  |  |  |
| Total | 0.19878796 | 53 |  |  |  |  |

1. **Herpes (Female)**

| Anova: Two-Factor Without Replication | |  |  |  |  |  |  |
| --- | --- | --- | --- | --- | --- | --- | --- |
| *SUMMARY* | *Validation AUC* | *Testing AUC* | *Ext Testing AUC* | *Count* | *Sum* | *Average* | *Variance* |
| RF | 0.870 | 0.881 | 0.933 | 3 | 2.684 | 0.895 | 0.001 |
| fine-tuned RF | 0.871 | 0.881 | 0.933 | 3 | 2.686 | 0.895 | 0.001 |
| Ensembled RF | 0.874 | 0.891 | 0.938 | 3 | 2.703 | 0.901 | 0.001 |
| Calibrated RF | 0.879 | 0.877 | 0.935 | 3 | 2.691 | 0.897 | 0.001 |
| ET | 0.865 | 0.868 | 0.932 | 3 | 2.664 | 0.888 | 0.001 |
| fine-tuned ET | 0.848 | 0.868 | 0.932 | 3 | 2.647 | 0.882 | 0.002 |
| Ensembled ET | 0.873 | 0.891 | 0.936 | 3 | 2.701 | 0.900 | 0.001 |
| Calibrated ET | 0.872 | 0.879 | 0.929 | 3 | 2.680 | 0.893 | 0.001 |
| GBC | 0.858 | 0.881 | 0.899 | 3 | 2.638 | 0.879 | 0.000 |
| fine-tuned GBC | 0.869 | 0.881 | 0.899 | 3 | 2.649 | 0.883 | 0.000 |
| Ensembled GBC | 0.864 | 0.891 | 0.922 | 3 | 2.677 | 0.892 | 0.001 |
| Calibrated GBC | 0.837 | 0.849 | 0.907 | 3 | 2.592 | 0.864 | 0.001 |
| XGBoost | 0.852 | 0.880 | 0.896 | 3 | 2.629 | 0.876 | 0.001 |
| fine-tuned XGBoost | 0.840 | 0.880 | 0.896 | 3 | 2.616 | 0.872 | 0.001 |
| Ensembled XGBoost | 0.876 | 0.894 | 0.906 | 3 | 2.676 | 0.892 | 0.000 |
| Calibrated XGBoost | 0.869 | 0.885 | 0.939 | 3 | 2.693 | 0.898 | 0.001 |
| **Blended model** | **0.885** | **0.887** | **0.936** | **3** | **2.707** | **0.902** | **0.001** |
| Stacked model | 0.765 | 0.776 | 0.859 | 3 | 2.400 | 0.800 | 0.003 |
| Validation AUC |  |  |  | 18 | 15.467 | 0.859 | 0.001 |
| Testing AUC |  |  |  | 18 | 15.741 | 0.875 | 0.001 |
| Ext Testing AUC |  |  |  | 18 | 16.527 | 0.918 | 0.000 |

| ANOVA |  |  |  |  |  |  |
| --- | --- | --- | --- | --- | --- | --- |
| *Source of Variation* | *SS* | *df* | *MS* | *F* | *P-value* | *F crit* |
| Rows | 0.02819825 | 17 | 0.001658721 | 12.4131432 | **5.6633E-10** | 1.93320683 |
| Columns | 0.03367332 | 2 | 0.016836658 | 125.998217 | **1.8924E-16** | 3.27589799 |
| Error | 0.00454329 | 34 | 0.000133626 |  |  |  |
| Total | 0.06641486 | 53 |  |  |  |  |

1. **Cystitis (Female)**

| Anova: Two-Factor Without Replication | |  |  |  |  |  |  |
| --- | --- | --- | --- | --- | --- | --- | --- |
| *SUMMARY* | *Validation AUC* | *Testing AUC* | *Ext Testing AUC* | *Count* | *Sum* | *Average* | *Variance* |
| RF | 0.834 | 0.887 | 0.791 | 3 | 2.512 | 0.837 | 0.002 |
| fine-tuned RF | 0.836 | 0.887 | 0.791 | 3 | 2.514 | 0.838 | 0.002 |
| Ensembled RF | 0.844 | 0.902 | 0.799 | 3 | 2.545 | 0.848 | 0.003 |
| Calibrated RF | 0.838 | 0.901 | 0.785 | 3 | 2.524 | 0.841 | 0.003 |
| ET | 0.834 | 0.887 | 0.791 | 3 | 2.512 | 0.837 | 0.002 |
| fine-tuned ET | 0.836 | 0.887 | 0.791 | 3 | 2.514 | 0.838 | 0.002 |
| Ensembled ET | 0.844 | 0.902 | 0.799 | 3 | 2.545 | 0.848 | 0.003 |
| Calibrated ET | 0.838 | 0.901 | 0.785 | 3 | 2.524 | 0.841 | 0.003 |
| LR | 0.829 | 0.907 | 0.819 | 3 | 2.555 | 0.852 | 0.002 |
| fine-tuned LR | 0.831 | 0.907 | 0.819 | 3 | 2.557 | 0.852 | 0.002 |
| Ensembled LR | 0.828 | 0.909 | 0.820 | 3 | 2.557 | 0.852 | 0.002 |
| Calibrated LR | 0.830 | 0.908 | 0.819 | 3 | 2.556 | 0.852 | 0.002 |
| GBC | 0.826 | 0.922 | 0.842 | 3 | 2.590 | 0.863 | 0.003 |
| fine-tuned GBC | 0.816 | 0.888 | 0.845 | 3 | 2.550 | 0.850 | 0.001 |
| **Ensembled GBC** | **0.840** | **0.928** | **0.843** | **3** | **2.611** | **0.870** | **0.003** |
| Calibrated GBC | 0.818 | 0.894 | 0.821 | 3 | 2.533 | 0.844 | 0.002 |
| Blended model | 0.845 | 0.921 | 0.825 | 3 | 2.591 | 0.864 | 0.003 |
| Stacked model | 0.764 | 0.828 | 0.704 | 3 | 2.296 | 0.765 | 0.004 |
| Validation AUC |  |  |  | 18 | 14.930 | 0.829 | 0.000 |
| Testing AUC |  |  |  | 18 | 16.165 | 0.898 | 0.000 |
| Ext Testing AUC |  |  |  | 18 | 14.488 | 0.805 | 0.001 |

| ANOVA |  |  |  |  |  |  |
| --- | --- | --- | --- | --- | --- | --- |
| *Source of Variation* | *SS* | *df* | *MS* | *F* | *P-value* | *F crit* |
| Rows | 0.02448955 | 17 | 0.001440562 | 7.16420787 | **6.3625E-07** | 1.93320683 |
| Columns | 0.08396041 | 2 | 0.041980203 | 208.776121 | **8.0372E-20** | 3.27589799 |
| Error | 0.00683664 | 34 | 0.000201078 |  |  |  |
| Total | 0.11528659 | 53 |  |  |  |  |

1. **Bacterial Vaginosis (Female)**

| Anova: Two-Factor Without Replication | |  |  |  |  |  |  |
| --- | --- | --- | --- | --- | --- | --- | --- |
| *SUMMARY* | *Validation AUC* | *Testing AUC* | *Ext Testing AUC* | *Count* | *Sum* | *Average* | *Variance* |
| GBC | 0.778 | 0.773 | 0.735 | 3 | 2.286 | 0.762 | 0.001 |
| fine-tuned GBC | 0.790 | 0.773 | 0.735 | 3 | 2.298 | 0.766 | 0.001 |
| Ensembled GBC | 0.784 | 0.788 | 0.735 | 3 | 2.307 | 0.769 | 0.001 |
| Calibrated GBC | 0.755 | 0.745 | 0.732 | 3 | 2.231 | 0.744 | 0.000 |
| CatBoost | 0.772 | 0.790 | 0.742 | 3 | 2.303 | 0.768 | 0.001 |
| fine-tuned CatBoost | 0.777 | 0.790 | 0.742 | 3 | 2.308 | 0.769 | 0.001 |
| Ensembled CatBoost | 0.778 | 0.794 | 0.748 | 3 | 2.320 | 0.773 | 0.001 |
| Calibrated CatBoost | 0.750 | 0.750 | 0.733 | 3 | 2.232 | 0.744 | 0.000 |
| LR | 0.770 | 0.794 | 0.763 | 3 | 2.327 | 0.776 | 0.000 |
| fine-tuned LR | 0.771 | 0.794 | 0.763 | 3 | 2.328 | 0.776 | 0.000 |
| Ensembled LR | 0.769 | 0.793 | 0.762 | 3 | 2.324 | 0.775 | 0.000 |
| Calibrated LR | 0.770 | 0.794 | 0.763 | 3 | 2.327 | 0.776 | 0.000 |
| RF | 0.765 | 0.780 | 0.746 | 3 | 2.291 | 0.764 | 0.000 |
| fine-tuned RF | 0.784 | 0.780 | 0.746 | 3 | 2.310 | 0.770 | 0.000 |
| Ensembled RF | 0.782 | 0.784 | 0.755 | 3 | 2.322 | 0.774 | 0.000 |
| Calibrated RF | 0.775 | 0.772 | 0.754 | 3 | 2.301 | 0.767 | 0.000 |
| **Blended model** | **0.783** | **0.800** | **0.750** | **3** | **2.333** | **0.778** | **0.001** |
| Stacked model | 0.753 | 0.786 | 0.752 | 3 | 2.290 | 0.763 | 0.000 |
| Validation AUC |  |  |  | 18 | 13.905 | 0.773 | 0.000 |
| Testing AUC |  |  |  | 18 | 14.080 | 0.782 | 0.000 |
| Ext Testing AUC |  |  |  | 18 | 13.455 | 0.747 | 0.000 |

| ANOVA |  |  |  |  |  |  |
| --- | --- | --- | --- | --- | --- | --- |
| *Source of Variation* | *SS* | *df* | *MS* | *F* | *P-value* | *F crit* |
| Rows | 0.00486242 | 17 | 0.000286025 | 3.01135752 | **0.00303271** | 1.93320683 |
| Columns | 0.0115536 | 2 | 0.0057768 | 60.8199284 | **5.8761E-12** | 3.27589799 |
| Error | 0.00322939 | 34 | 9.4982E-05 |  |  |  |
| Total | 0.01964541 | 53 |  |  |  |  |

1. **Candidiasis (Female)**

| Anova: Two-Factor Without Replication | |  |  |  |  |  |  |
| --- | --- | --- | --- | --- | --- | --- | --- |
| *SUMMARY* | *Validation AUC* | *Testing AUC* | *Ext Testing AUC* | *Count* | *Sum* | *Average* | *Variance* |
| GBC | 0.785 | 0.763 | 0.772 | 3 | 2.320 | 0.773 | 0.000 |
| fine-tuned GBC | 0.762 | 0.763 | 0.772 | 3 | 2.298 | 0.766 | 0.000 |
| Ensembled GBC | 0.792 | 0.766 | 0.777 | 3 | 2.336 | 0.779 | 0.000 |
| Calibrated GBC | 0.767 | 0.726 | 0.773 | 3 | 2.266 | 0.755 | 0.001 |
| LR | 0.784 | 0.766 | 0.778 | 3 | 2.328 | 0.776 | 0.000 |
| fine-tuned LR | 0.783 | 0.765 | 0.779 | 3 | 2.327 | 0.776 | 0.000 |
| Ensembled LR | 0.786 | 0.768 | 0.777 | 3 | 2.330 | 0.777 | 0.000 |
| Calibrated LR | 0.785 | 0.766 | 0.779 | 3 | 2.330 | 0.777 | 0.000 |
| RF | 0.774 | 0.754 | 0.738 | 3 | 2.266 | 0.755 | 0.000 |
| fine-tuned RF | 0.793 | 0.754 | 0.738 | 3 | 2.285 | 0.762 | 0.001 |
| Ensembled RF | 0.787 | 0.762 | 0.751 | 3 | 2.300 | 0.767 | 0.000 |
| Calibrated RF | 0.783 | 0.741 | 0.729 | 3 | 2.253 | 0.751 | 0.001 |
| CatBoost | 0.765 | 0.762 | 0.756 | 3 | 2.282 | 0.761 | 0.000 |
| fine-tuned CatBoost | 0.791 | 0.761 | 0.771 | 3 | 2.323 | 0.774 | 0.000 |
| Ensembled CatBoost | 0.783 | 0.758 | 0.767 | 3 | 2.308 | 0.769 | 0.000 |
| Calibrated CatBoost | 0.743 | 0.700 | 0.739 | 3 | 2.182 | 0.727 | 0.001 |
| **Blended model** | **0.792** | **0.773** | **0.775** | **3** | **2.340** | **0.780** | **0.000** |
| Stacked model | 0.768 | 0.753 | 0.732 | 3 | 2.253 | 0.751 | 0.000 |
| Validation AUC |  |  |  | 18 | 14.024 | 0.779 | 0.000 |
| Testing AUC |  |  |  | 18 | 13.602 | 0.756 | 0.000 |
| Ext Testing AUC |  |  |  | 18 | 13.703 | 0.761 | 0.000 |

| ANOVA |  |  |  |  |  |  |
| --- | --- | --- | --- | --- | --- | --- |
| *Source of Variation* | *SS* | *df* | *MS* | *F* | *P-value* | *F crit* |
| Rows | 0.0094018 | 17 | 0.000553047 | 4.11052903 | **0.00022284** | 1.93320683 |
| Columns | 0.00539059 | 2 | 0.002695294 | 20.0328061 | **1.7851E-06** | 3.27589799 |
| Error | 0.0045745 | 34 | 0.000134544 |  |  |  |
| Total | 0.01936689 | 53 |  |  |  |  |

1. **Molluscum Contagiosum (Female)**

| Anova: Two-Factor Without Replication | |  |  |  |  |  |  |
| --- | --- | --- | --- | --- | --- | --- | --- |
| *SUMMARY* | *Validation AUC* | *Testing AUC* | *Ext Testing AUC* | *Count* | *Sum* | *Average* | *Variance* |
| **RF** | **0.939** | **0.901** | **0.950** | **3** | **2.790** | **0.930** | **0.001** |
| fine-tuned RF | 0.926 | 0.901 | 0.950 | 3 | 2.777 | 0.926 | 0.001 |
| Ensembled RF | 0.943 | 0.893 | 0.949 | 3 | 2.784 | 0.928 | 0.001 |
| Calibrated RF | 0.937 | 0.889 | 0.949 | 3 | 2.774 | 0.925 | 0.001 |
| XGBoost | 0.929 | 0.841 | 0.943 | 3 | 2.713 | 0.904 | 0.003 |
| fine-tuned XGBoost | 0.913 | 0.841 | 0.943 | 3 | 2.697 | 0.899 | 0.003 |
| Ensembled XGBoost | 0.940 | 0.871 | 0.947 | 3 | 2.758 | 0.919 | 0.002 |
| Calibrated XGBoost | 0.931 | 0.876 | 0.882 | 3 | 2.688 | 0.896 | 0.001 |
| ET | 0.923 | 0.875 | 0.938 | 3 | 2.736 | 0.912 | 0.001 |
| fine-tuned ET | 0.917 | 0.875 | 0.938 | 3 | 2.731 | 0.910 | 0.001 |
| Ensembled ET | 0.940 | 0.891 | 0.945 | 3 | 2.775 | 0.925 | 0.001 |
| Calibrated ET | 0.940 | 0.900 | 0.943 | 3 | 2.783 | 0.928 | 0.001 |
| CatBoost | 0.915 | 0.879 | 0.955 | 3 | 2.750 | 0.917 | 0.001 |
| fine-tuned CatBoost | 0.931 | 0.879 | 0.955 | 3 | 2.765 | 0.922 | 0.001 |
| Ensembled CatBoost | 0.919 | 0.885 | 0.968 | 3 | 2.773 | 0.924 | 0.002 |
| Calibrated CatBoost | 0.916 | 0.855 | 0.959 | 3 | 2.730 | 0.910 | 0.003 |
| Blended model | 0.944 | 0.869 | 0.947 | 3 | 2.760 | 0.920 | 0.002 |
| Stacked model | 0.891 | 0.849 | 0.922 | 3 | 2.662 | 0.887 | 0.001 |
| Validation AUC |  |  |  | 18 | 16.693 | 0.927 | 0.000 |
| Testing AUC |  |  |  | 18 | 15.771 | 0.876 | 0.000 |
| Ext Testing AUC |  |  |  | 18 | 16.983 | 0.943 | 0.000 |

| ANOVA |  |  |  |  |  |  |
| --- | --- | --- | --- | --- | --- | --- |
| *Source of Variation* | *SS* | *df* | *MS* | *F* | *P-value* | *F crit* |
| Rows | 0.00774457 | 17 | 0.000455563 | 2.11482808 | **0.03094029** | 1.93320683 |
| Columns | 0.04449948 | 2 | 0.022249739 | 103.288452 | **3.5795E-15** | 3.27589799 |
| Error | 0.00732406 | 34 | 0.000215414 |  |  |  |
| Total | 0.05956811 | 53 |  |  |  |  |

**Figure S3. How to interpret the SHAP analysis**

The y-axis shows features ranked from highest to lowest importance. In the feature visualisation, red indicates higher values for continuous variables (e.g., older age, higher number of sexual partners) and the presence of symptoms for categorical variables (e.g., unusual urethral discharge, Painful urination). The x-axis shows the SHAP value, which measures how much each feature affects the prediction. A lower SHAP value means the feature has a negative impact on predicting STIs, while a higher value means it has a positive impact.

For example, the presence of unusual urethral discharge has a positive influence on predicting gonorrhoea in Figure S3A. A higher number of casual female sexual partners within the past 12 months has a positive influence on predicting molluscum contagiosum in Figure S3D. A wide range of values on the x-axis indicates a larger variation in the impact of the feature on the prediction of STIs. In contrast, a narrow range on the x-axis indicates a more consistent impact of that feature on STI prediction.

**Figure** **S3. Interpretation of machine learning models for conditions in males**


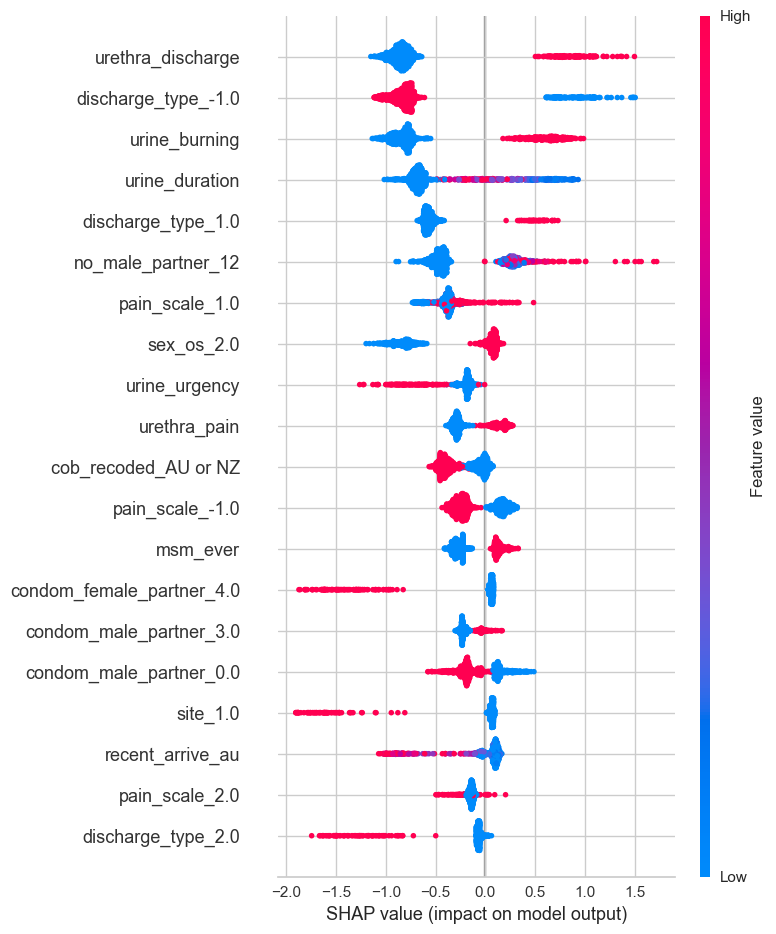


1. **Random Forest (Male, Gonorrhoea)**

The Random Forest model identified the following factors in order of importance as strongly indicative of a positive gonorrhoea diagnosis in males:

- Presence of unusual urethral discharge
- Painful or uncomfortable urination
- Thick, green, or yellow urethra discharge occurring 1-2 days after sexual intercourse
- Mild pain during urination
- Pain or discomfort at the tip of urethra
- Country of birth outside Australia and New Zealand
- Being a man who has sex with men (MSM)
- Never using condom with casual male partners within the past 12 months

The presence of these factors increased the SHAP (Shapley Additive exPlanations) values, which contributed to a higher likelihood of a positive gonorrhoea prediction by the model.


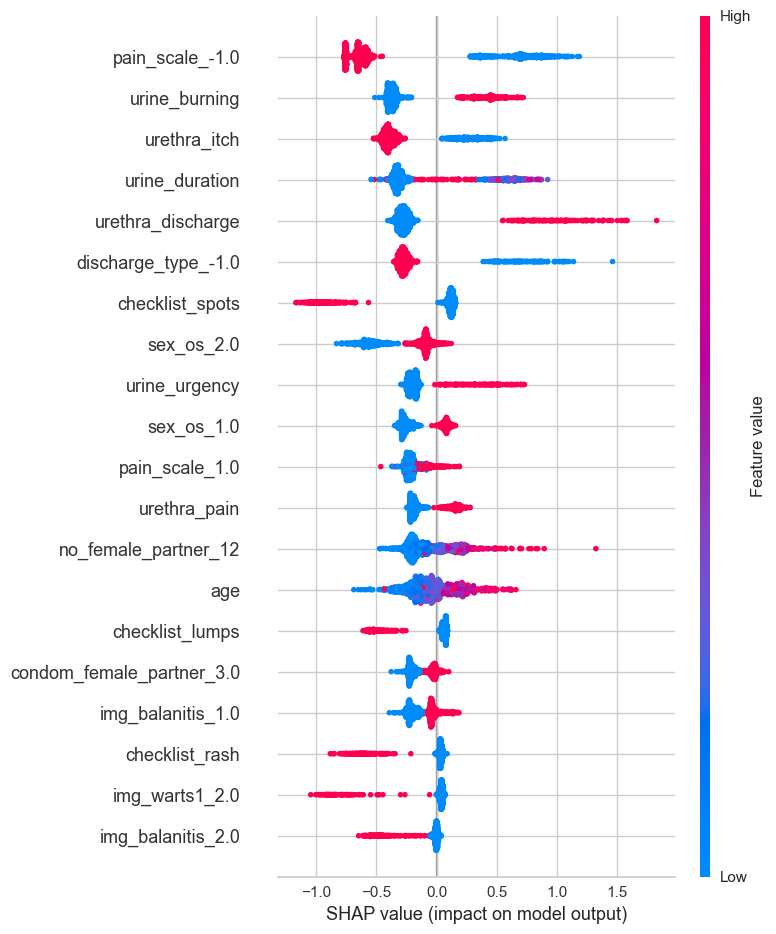


1. **CatBoost (Male, Non-gonococcal Urethritis)**

The CatBoost model identified the following factors in order of importance as strongly indicative of a positive urethritis diagnosis in males:

- Painful or uncomfortable urination
- Absence of itchiness at the tip of urethra
- Presence of unusual urethral discharge
- Absence of anogenital skin lesion (spot, lumps)
- Urge or frequent urination
- Having had sexual intercourse oversea in the past 12 months
- Mild pain during urination
- Pain or discomfort at the tip of urethra
- Higher number of casual female sexual partners within the past 12 months

The presence of these factors increased the SHAP values, which contributed to a higher likelihood of a positive NGU prediction by the model.


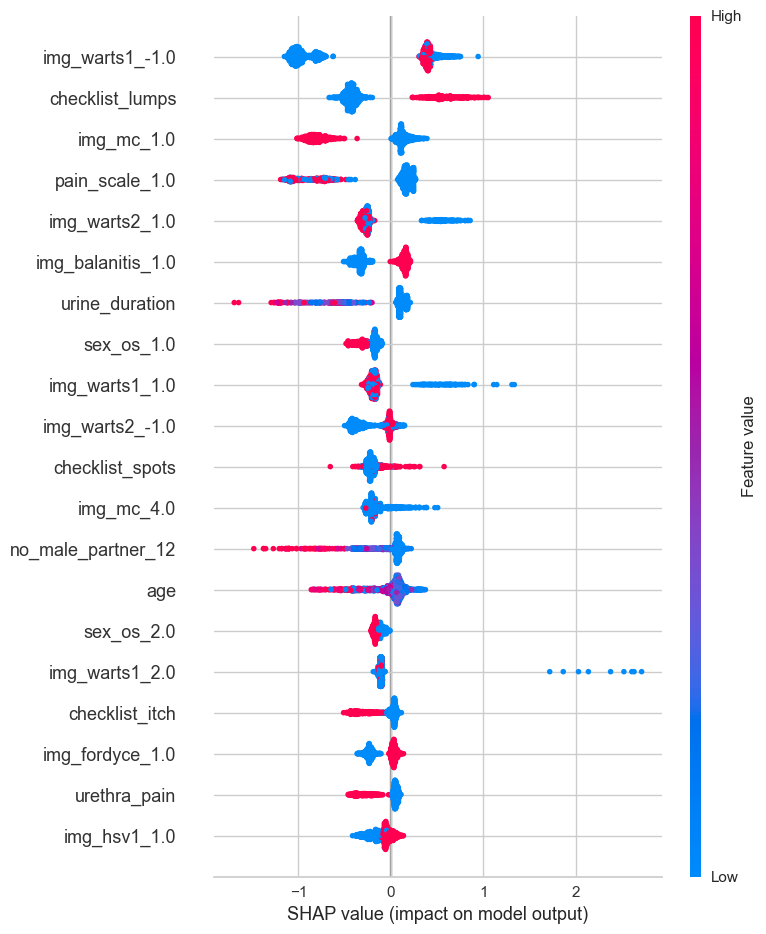


1. **CatBoost (Male, Genital Warts)**

The CatBoost model identified the following factors in order of importance as strongly indicative of a positive genital warts diagnosis in males:

- Presence of anogenital skin lesion (lumps, spots)
- Lesions not resembling the presented images of balanitis, herpes and Fordyce spots
- Lesion resembling the presented image of warts
- Absence of itchiness and urethral pain

The presence of these factors increased the SHAP values, which contributed to a higher likelihood of a positive genital warts prediction by the model.


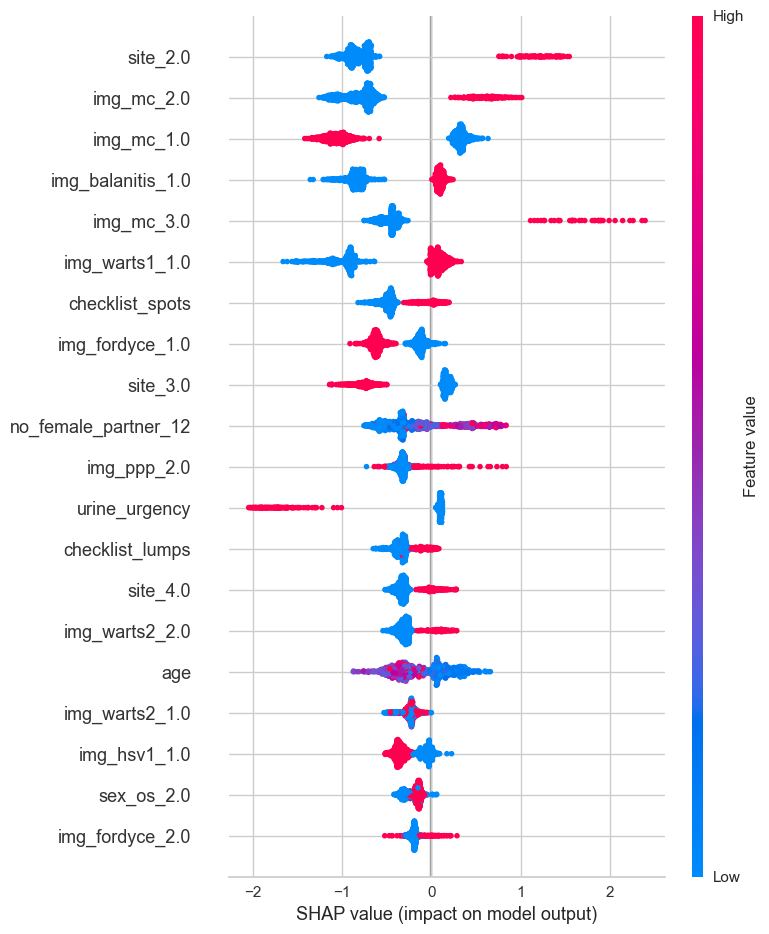


1. **CatBoost (Male, Molluscum Contagiosum)**

The CatBoost model identified the following factors in order of importance as strongly indicative of a positive molluscum contagiosum diagnosis in males:

- Presence of lesions at the groin crease
- Lesions closely resembling or an exact match to the presented molluscum image
- Lesions not resembling the presented images of balanitis or warts
- Presence of anogenital skin lesion (spots)
- Absence of lesions at the glans penis
- Higher number of casual female sexual partners within the past 12 months
- Lesions partially resembling penile papules of warts images
- Absence of urinary symptoms (urgency or frequency)

The presence of these factors increased the SHAP values, which contributed to a higher likelihood of a positive molluscum contagiosum prediction by the model.


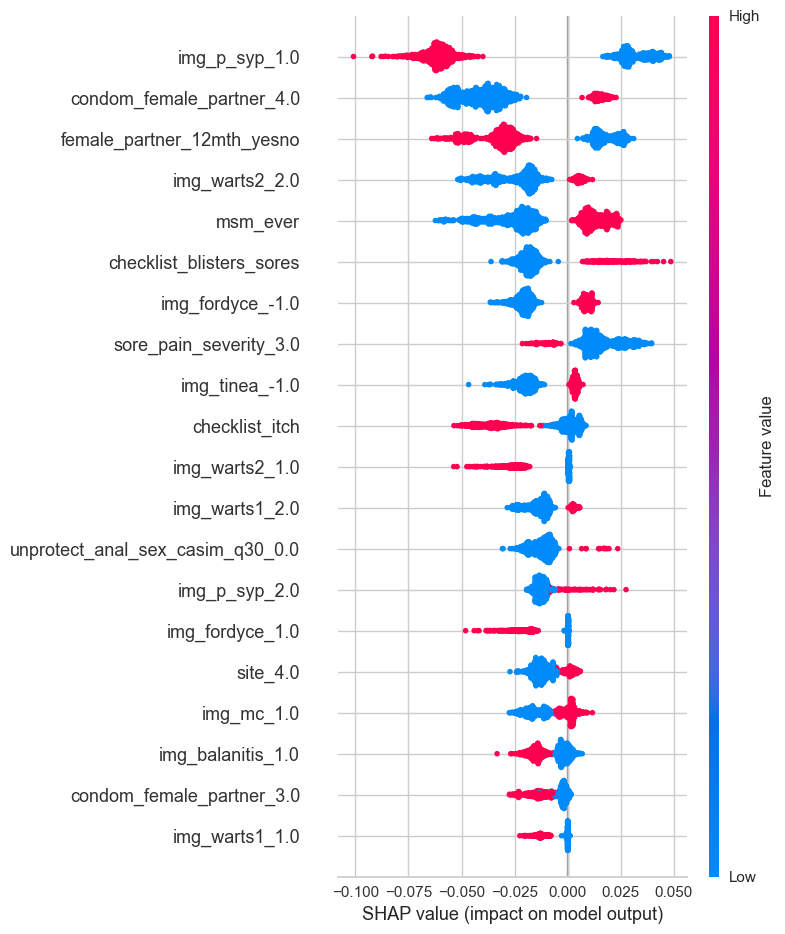


1. **Random Forest (Male, Syphilis)**

The Random Forest model identified the following factors in order of importance as strongly indicative of a positive balanitis diagnosis in males:

- Lesions resembling the presented images of syphilis
- Lesion partially resembling to the presented images of warts
- Being a MSM
- Lesions presented as blisters or sores
- Absence of itchiness at the lesion site
- Lesions not resembling Fordyce spots, warts, molluscum contagiosum or balanitis
- Having had unprotected anal sex

The presence of these factors increased the SHAP values, which contributed to a higher likelihood of a positive syphilis prediction by the model.


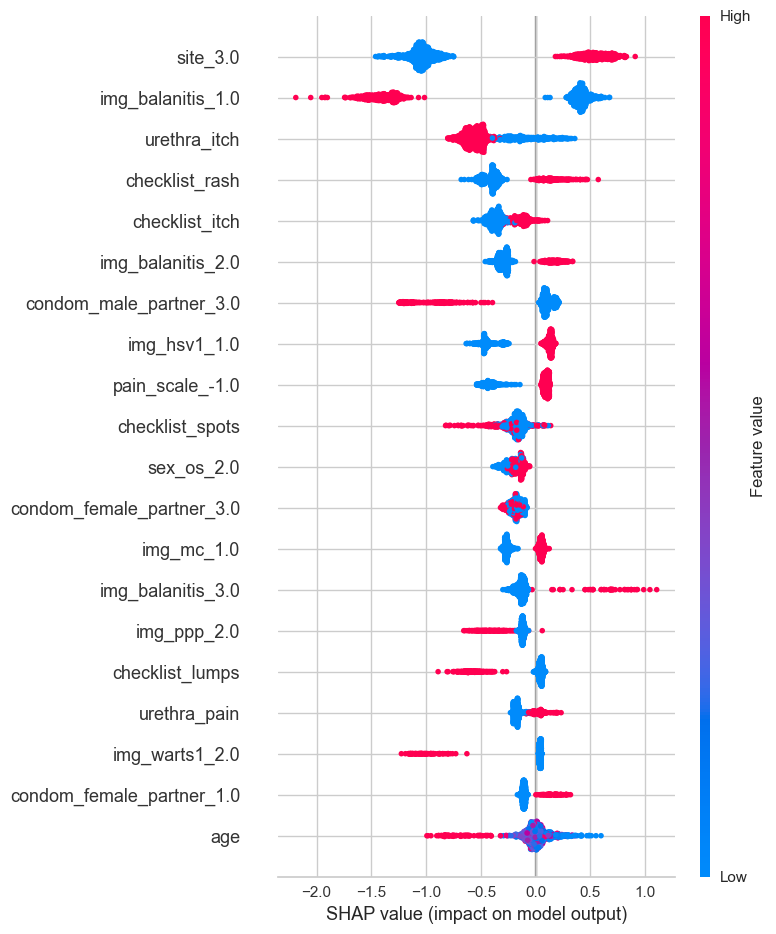


1. **CatBoost (Male, Balanitis)**

The CatBoost model identified the following factors in order of importance as strongly indicative of a positive balanitis diagnosis in males:

- Presence of lesions on the glans penis
- Lesions resembling the presented images of balanitis
- Presence of anogenital skin lesion (rash and itching)
- Consistent condom use with casual female sexual partners with the past 12 months
- Lesions not resembling the presented images of herpes and molluscum
- Presence of pain at the tip of urethra
- older age

The presence of these factors increased the SHAP values, which contributed to a higher likelihood of a positive balanitis prediction by the model.


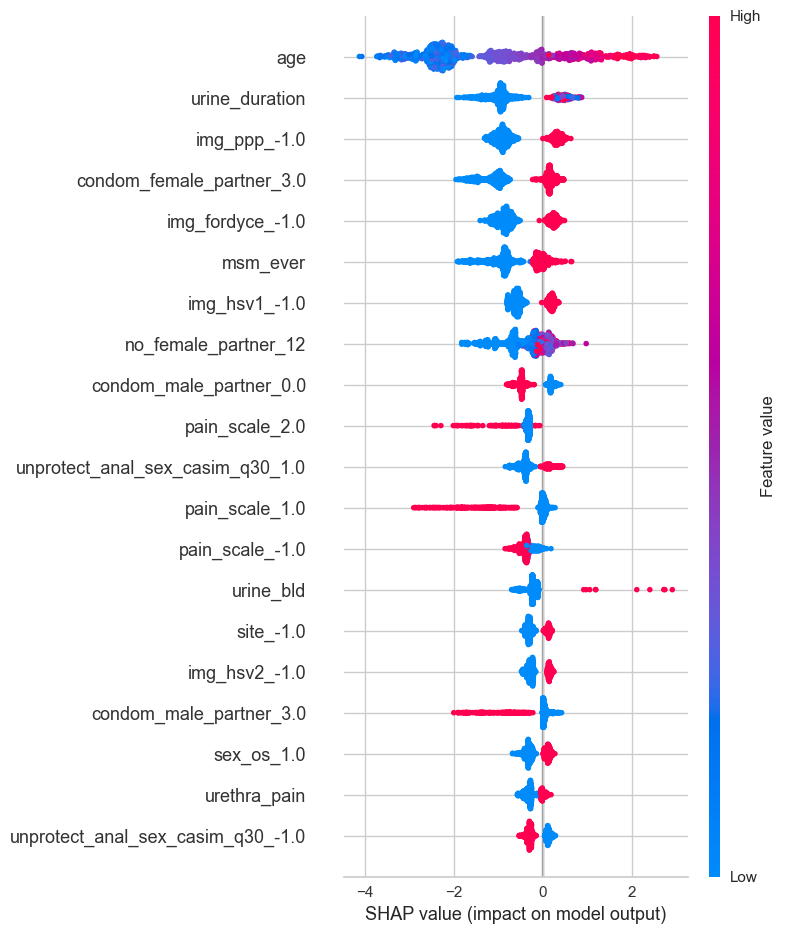


1. **CatBoost (Male, UTI)**

The CatBoost model identified the following factors in order of importance as strongly indicative of a positive UTI diagnosis in males:

- Older age
- Longer duration of urinary symptoms
- Consistent condom uses with casual female sexual partners within past 12 months
- Being a MSM
- Higher number of casual female sexual partners
- Having had unprotected anal sex
- Inconsistent condom uses with casual male sexual partners within past 12 months

The presence of these factors increased the SHAP values, which contributed to a higher likelihood of a positive UTI prediction by the model.


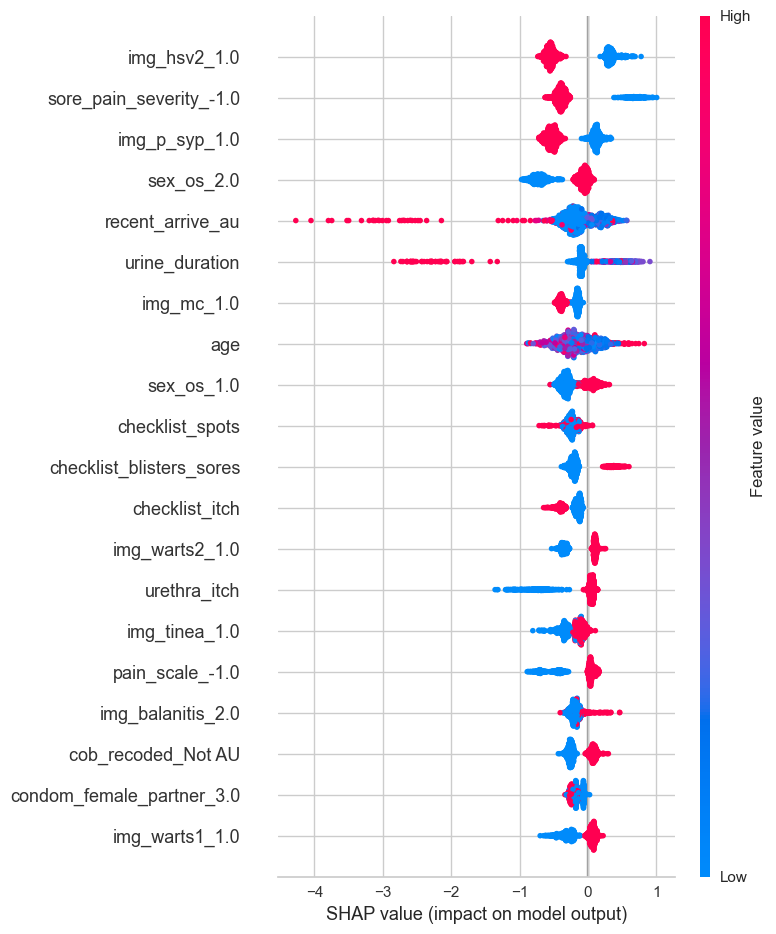


1. **CatBoost (Male, Herpes)**

The CatBoost model identified the following factors in order of importance as strongly indicative of a positive herpes diagnosis in males:

- Lesions resembling the presented images of herpes
- Presence of painful sores or blisters
- Shorter duration of urinary symptoms
- Lesions not resembling syphilis, molluscum, warts, or tinea cruris
- Having had sexual intercourse oversea with 12 months
- Presence of itchiness at urethra
- Country of birth outside Australia or New Zealand

The presence of these factors increased the SHAP values, which contributed to a higher likelihood of a positive herpes prediction by the model.


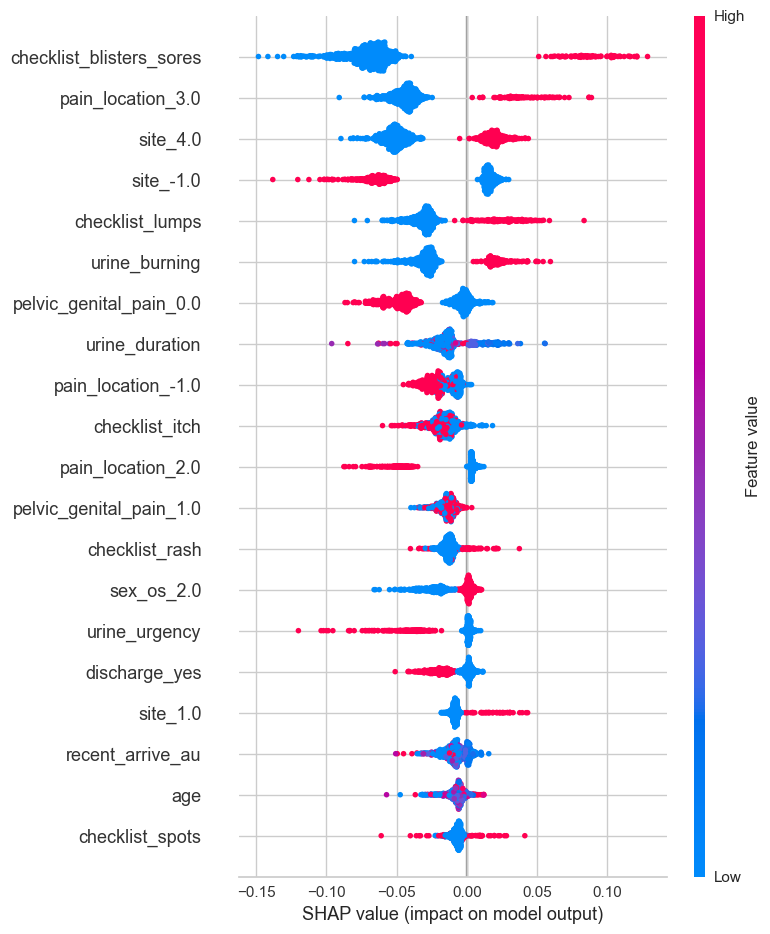


1. **Random Forest (Female, Herpes)**

The Random Forest model identified the following factors in order of importance as strongly indicative of a positive herpes diagnosis in females:

- Presence of anogenital lesions (blister, sores, lump or rash)
- Lesion location near public hair area, vulva, around the bottom or anus
- Presence of painful or uncomfortable urination
- Absence of pelvic pain
- Absence of itchiness at the lesion area
- Presence of pain at vulva or vagina
- Absence of urinary symptoms (frequency or urgency) and unusual vaginal discharge

The presence of these factors increased the SHAP values, which contributed to a higher likelihood of a positive herpes contagiosum prediction by the model.

1. **Random Forest (Female, Molluscum Contagiosum)**

The Random Forest model identified the following factors in order of importance as strongly indicative of a positive molluscum contagiosum diagnosis in females:

- Lesions partially resembling the presented images of molluscum contagiosum or warts
- Lesions not resembling herpes or vestibular glands
- Presence of anogenital lesion (lumps or spots)
- Absence of itchiness at the lesion site
- Absence of painful or uncomfortable urination

The presence of these factors increased the SHAP values, which contributed to a higher likelihood of a positive molluscum contagiosum prediction by the model.


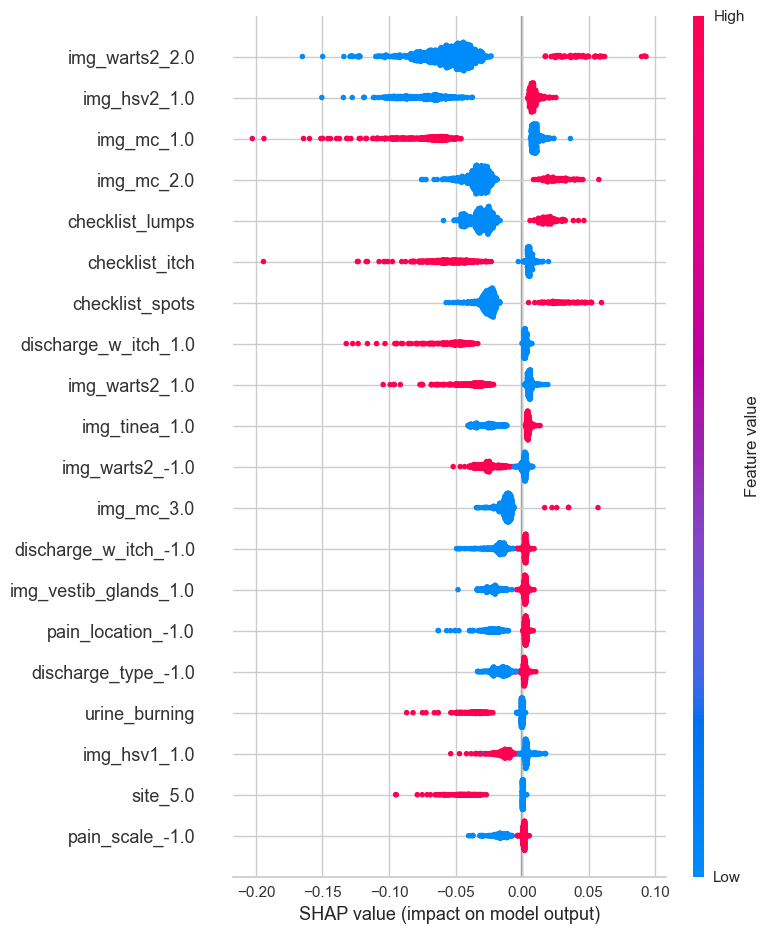

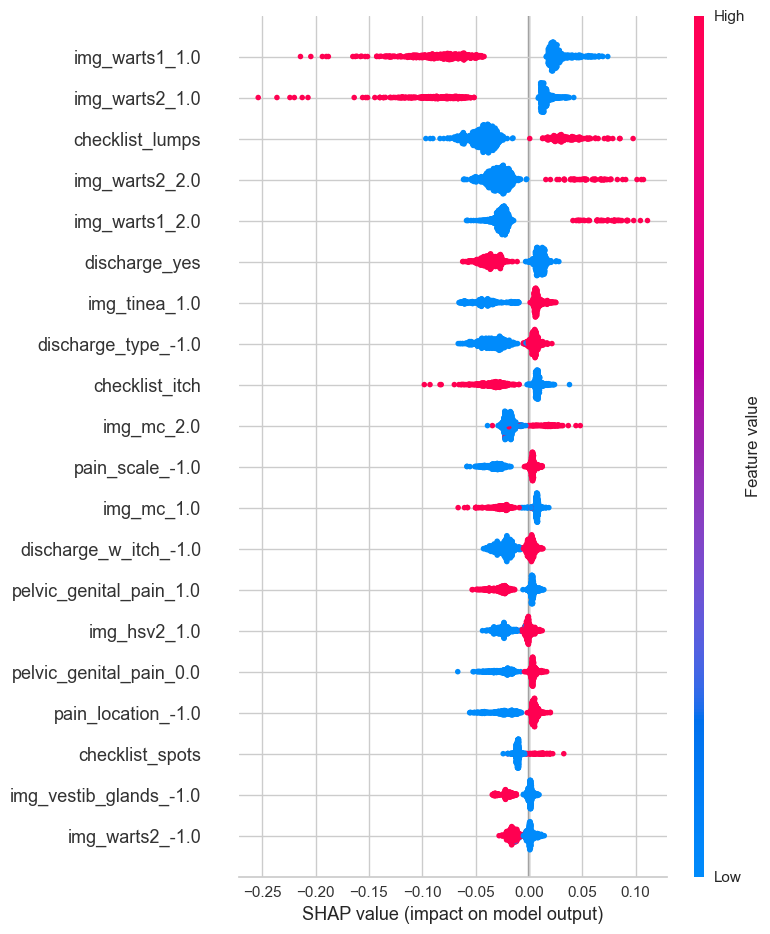


1. **Random Forest (Female, Warts)**

The Random Forest model identified the following factors in order of importance as strongly indicative of a positive warts diagnosis in females:

- Lesions resembling the presented image of genital warts
- Presence of anogenital lesion (lump or spots)
- Absence of unusual vaginal discharge
- Lesions not resembling to the images of tinea cruris, herpes or vestibular glands
- Lesions partially resembling molluscum contagiosum
- Absence of itchiness at the lesion area or pelvic pain

The presence of these factors increased the SHAP values, which contributed to a higher likelihood of a positive warts prediction by the model.


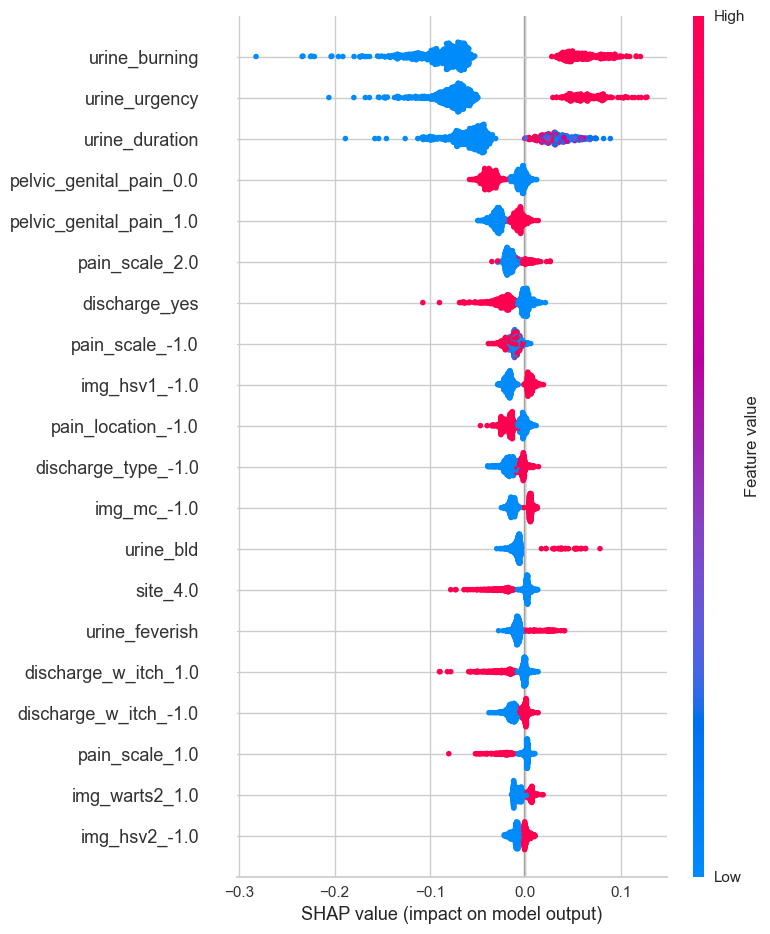


1. **Random Forest (Female, Cystitis)**

The Random Forest model identified the following factors in order of importance as strongly indicative of a positive cystitis diagnosis in females:

- Presence of painful or uncomfortable urination and increased frequency/urgency of urination
- Presence of moderate pain in pelvic or genital area
- Absence of itchy discharge
- Presence of blood in the urine
- Absence of pain in the vulva
- Associated with fever

The presence of these factors increased the SHAP values, which contributed to a higher likelihood of a positive cystitis contagiosum prediction by the model.


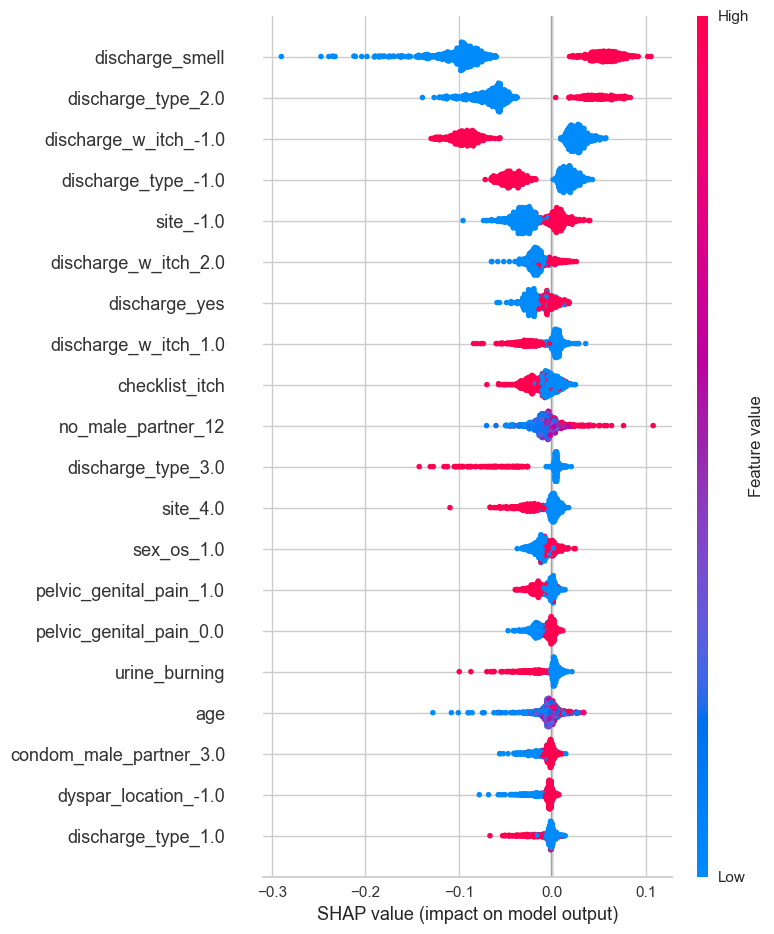


1. **Random Forest (Female, Bacterial Vaginosis)**

The Random Forest model identified the following factors in order of importance as strongly indicative of a positive bacterial vaginosis diagnosis in females:

- Presence of unusual vaginal discharge with unpleasant smell
- Presence of pale or greyish discharge with fishy smell
- Absence of discharge associated with itchiness
- Absence of anogenital skin lesion (itching)
- Higher number of casual male sexual partners within 12 months
- Absence of pelvic pain
- Absence of painful or uncomfortable urination
- Consistent condom use with casual male partners within the past 12 months
- Absence of thickish, clumpy or white discharge

The presence of these factors increased the SHAP values, which contributed to a higher likelihood of a positive bacterial vaginosis prediction by the model.


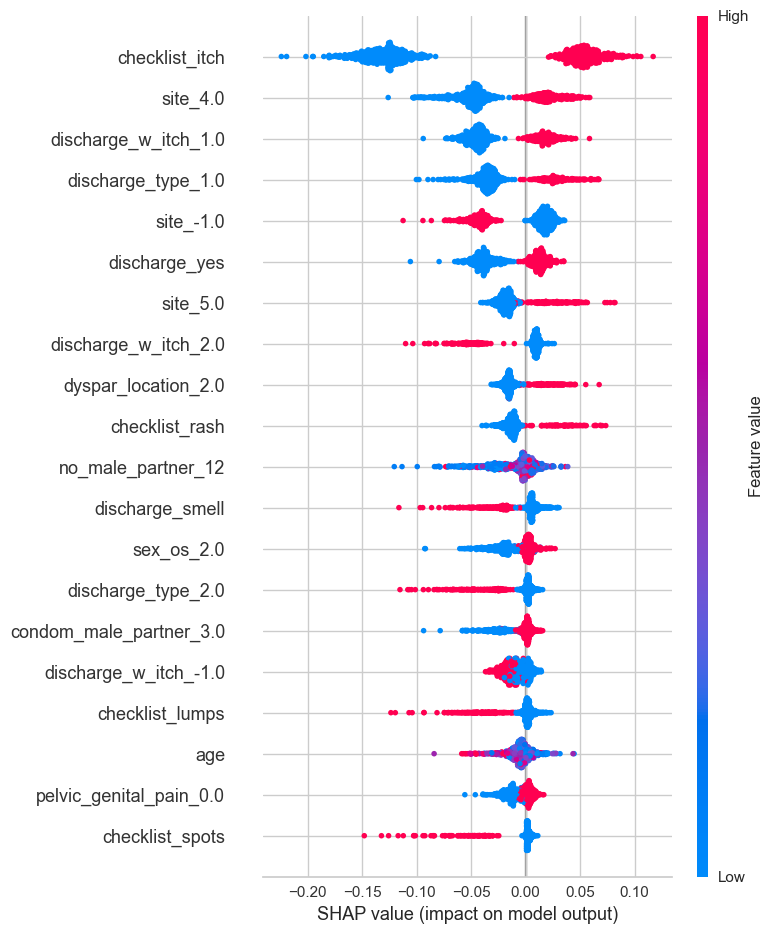


1. **Random Forest (Female, Candidiasis)**

The Random Forest model identified the following factors in order of importance as strongly indicative of a positive candidiasis diagnosis in females:

- Presence of itchiness at the vulva or inside the vagina
- Presence of discharge associated with itchiness
- Absence of dyspareunia at vulva or vagina
- Presence of anogenital rash
- Absence of unusual vaginal discharge with unpleasant smell
- No sexual intercourse overseas within past 12 months
- Absence of pale or greyish discharge with fishy smell
- Consistent condom use with casual male partners within the past 12 months
- Absence of pelvic pain

The presence of these factors increased the SHAP values, which contributed to a higher likelihood of a positive candidiasis prediction by the model.


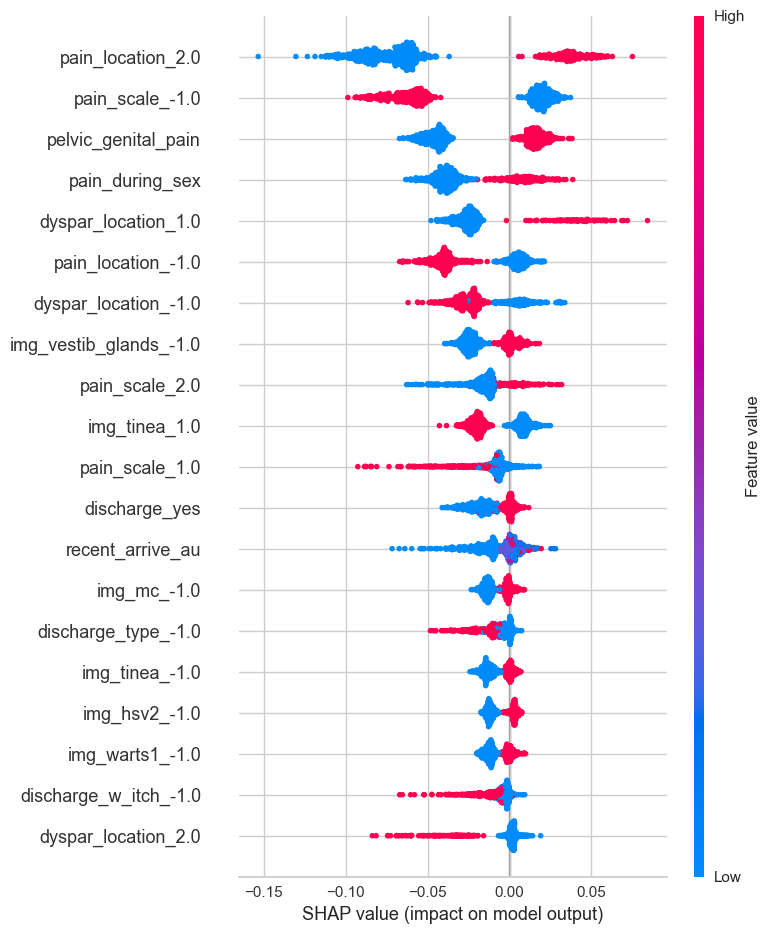


1. **Random Forest (Female, Pelvic Inflammatory Disease)**

The Random Forest model identified the following factors in order of importance as strongly indicative of a positive PID diagnosis in females:

- Presence of pain in pelvis or lower part of tummy
- Presence of pain during sex
- Presence of moderate pain
- Presence of unusual vaginal discharge
- Absence of anogenital skin lesion

The presence of these factors increased the SHAP values, which contributed to a higher likelihood of a positive PID prediction by the model.


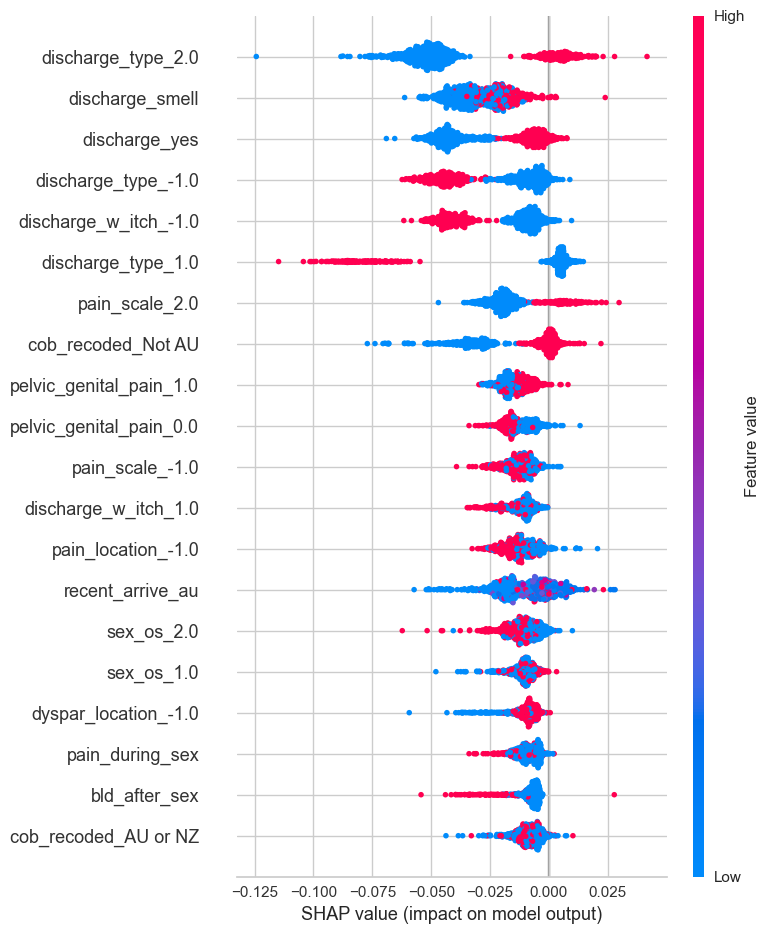


1. **Random Forest (Female, Cervicitis)**

The Random Forest model identified the following factors in order of importance as strongly indicative of a positive cervicitis diagnosis in females:

- Presence of pale or greyish discharge with fishy smell
- Presence of moderate pain
- Country of birth outside Australia or New Zealand

The presence of these factors increased the SHAP values, which contributed to a higher likelihood of a positive cervicitis prediction by the model.

Figure S4. Precision-Recall and Sensitivity-Specificity curves for Bayesian and Machine learning models for each disease condition

A.1. Bayesian Model (Male, Syphilis)

**
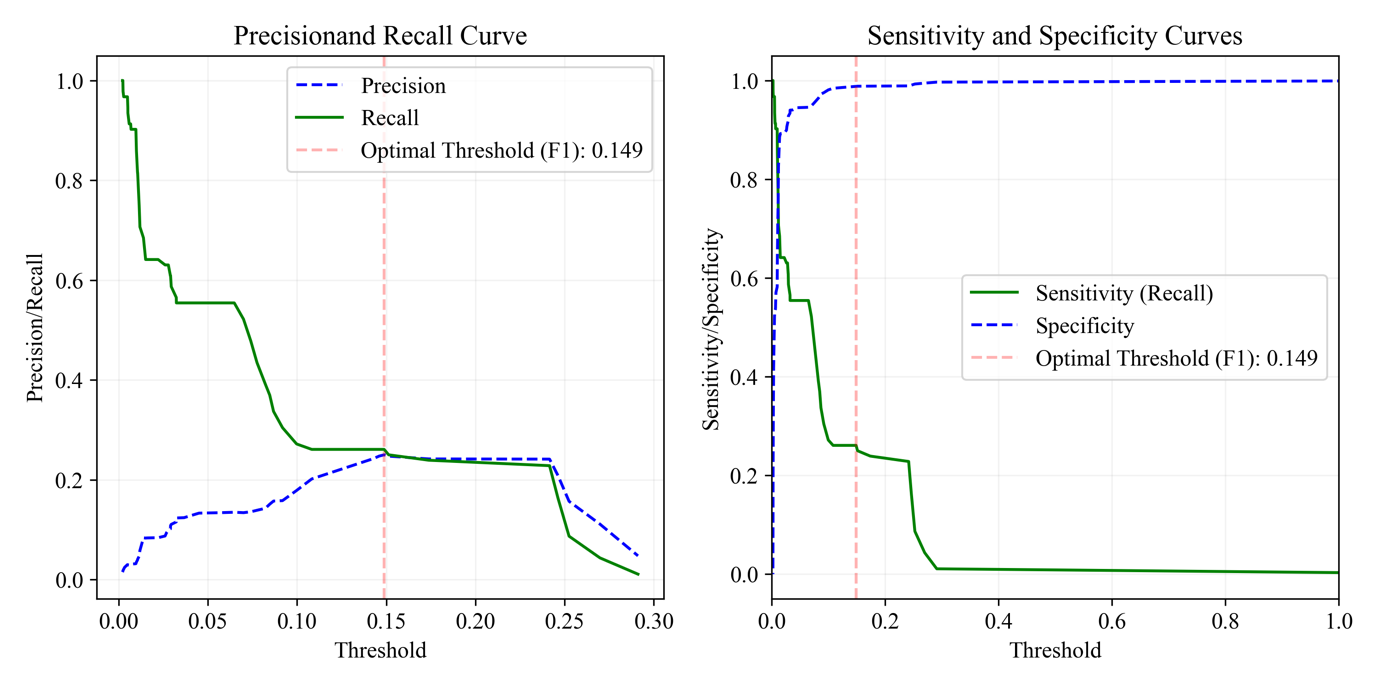
**

A.2. Machine Learning Model (Male, Syphilis)

**
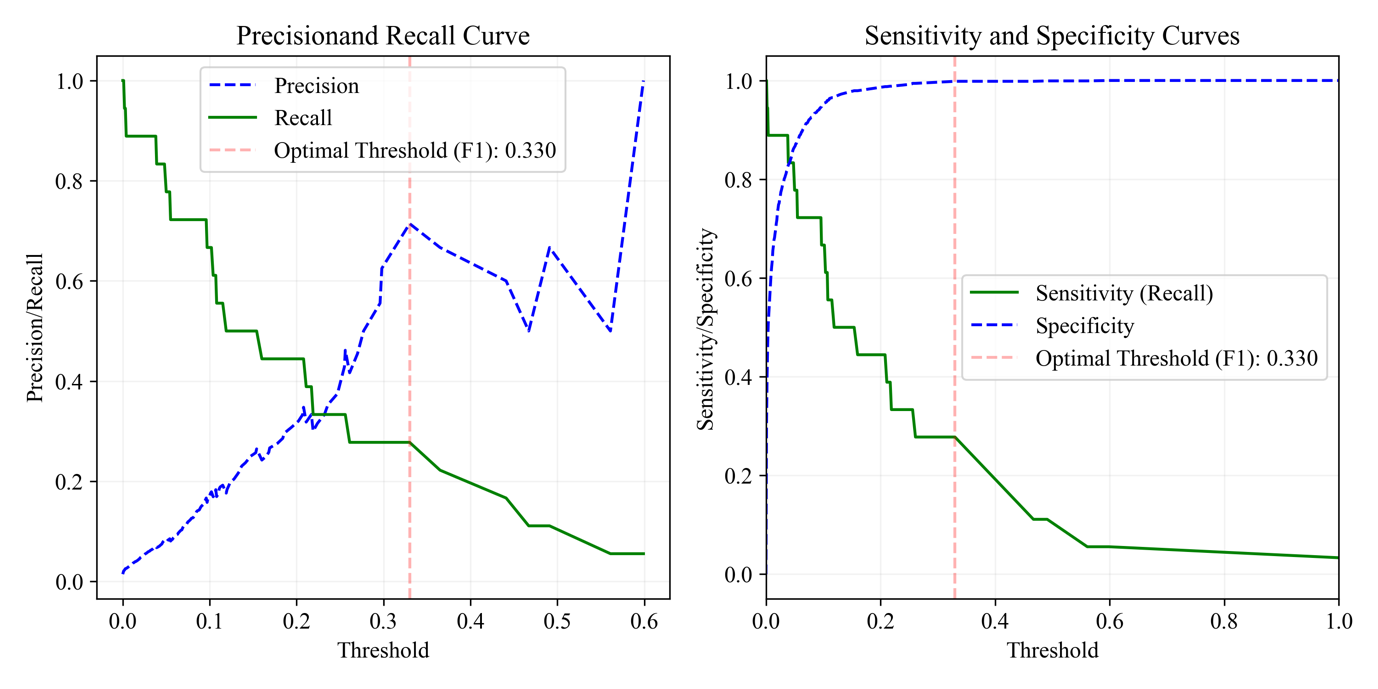
**

B.1. Bayesian Model (Male, Warts)

**
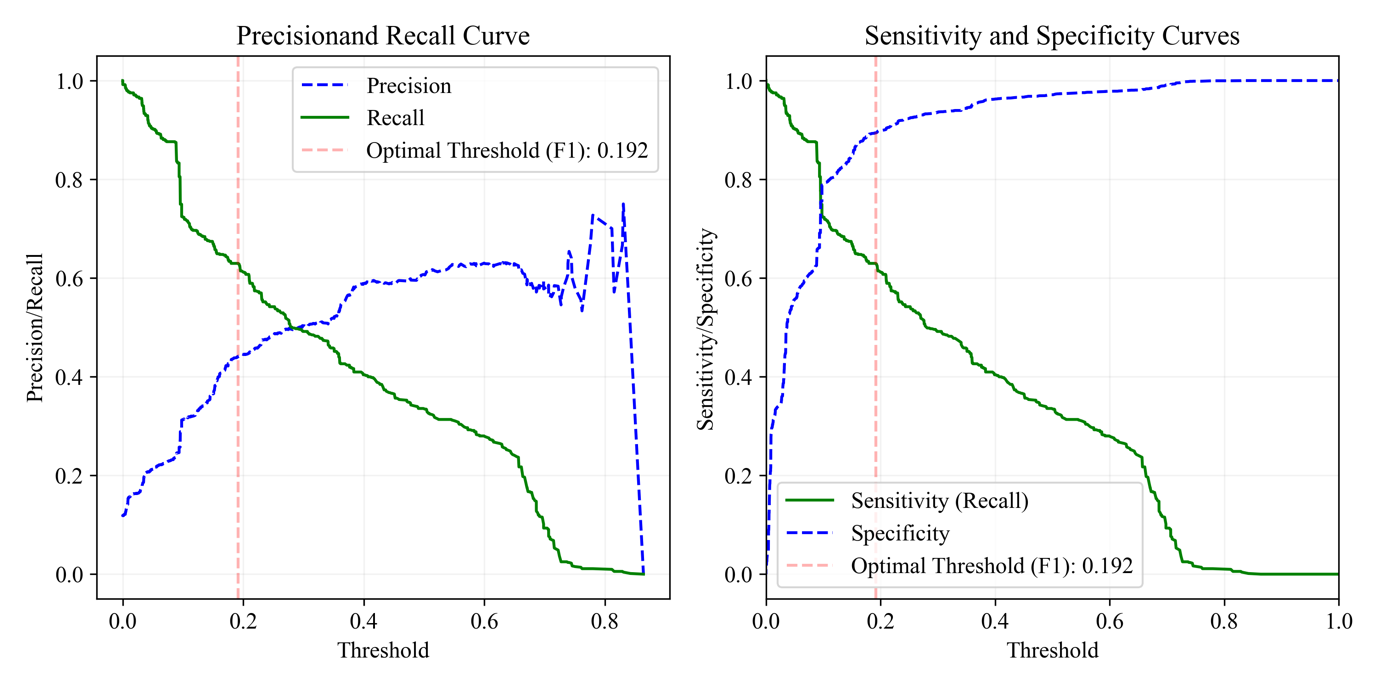
**

B.2. Machine Learning Model (Male, Warts)

**
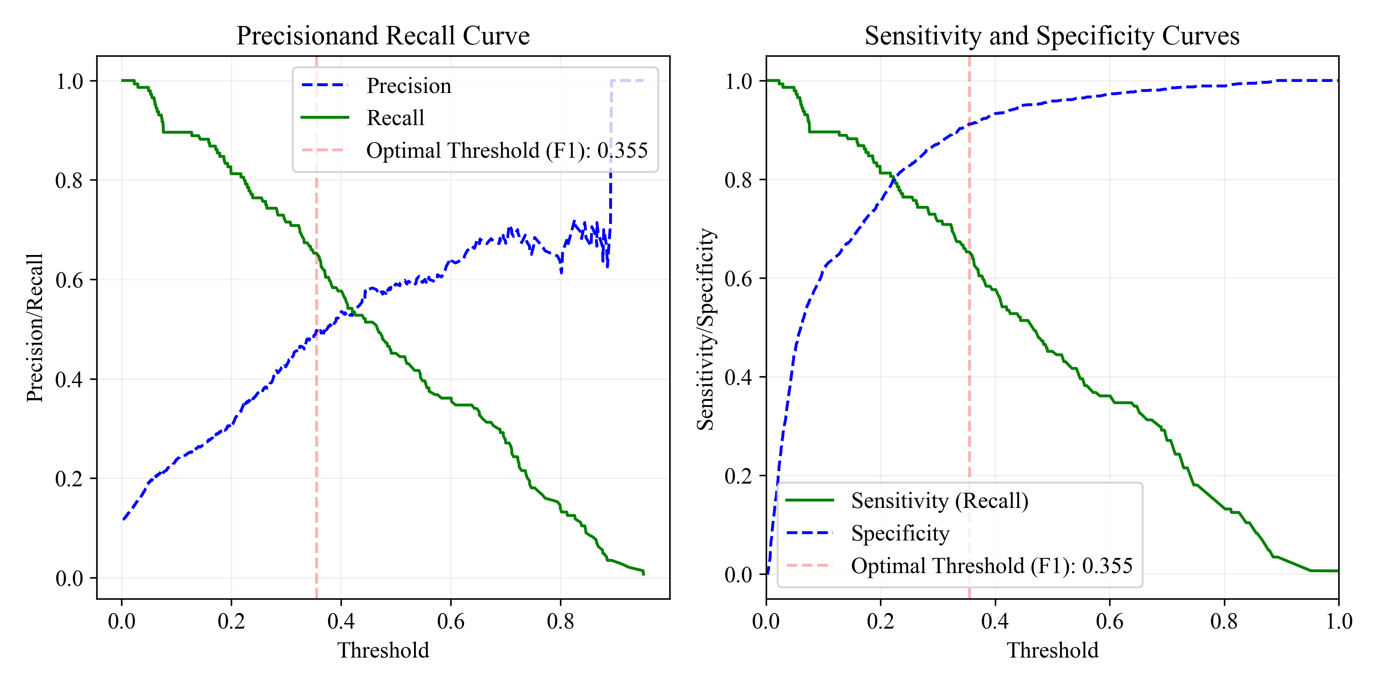
**

C.1. Bayesian Model (Male, NGU)


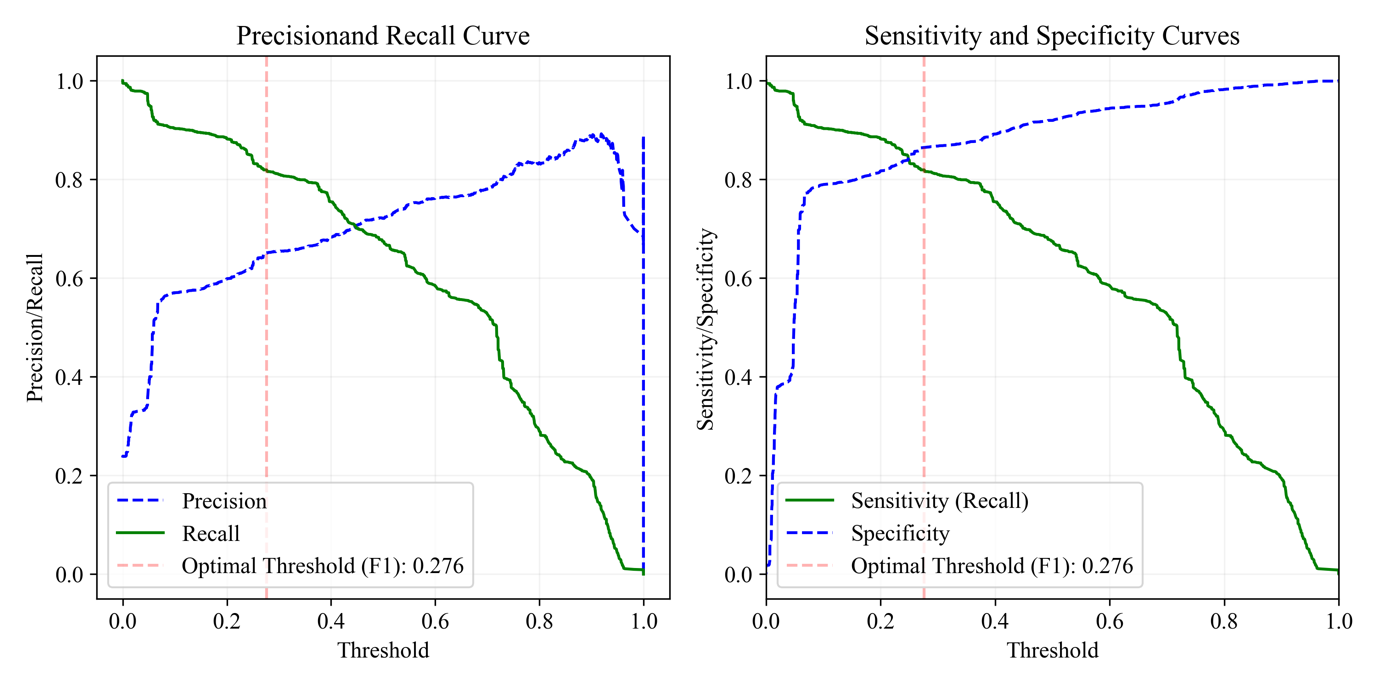


C.2. Machine Learning Model (Male, NGU)


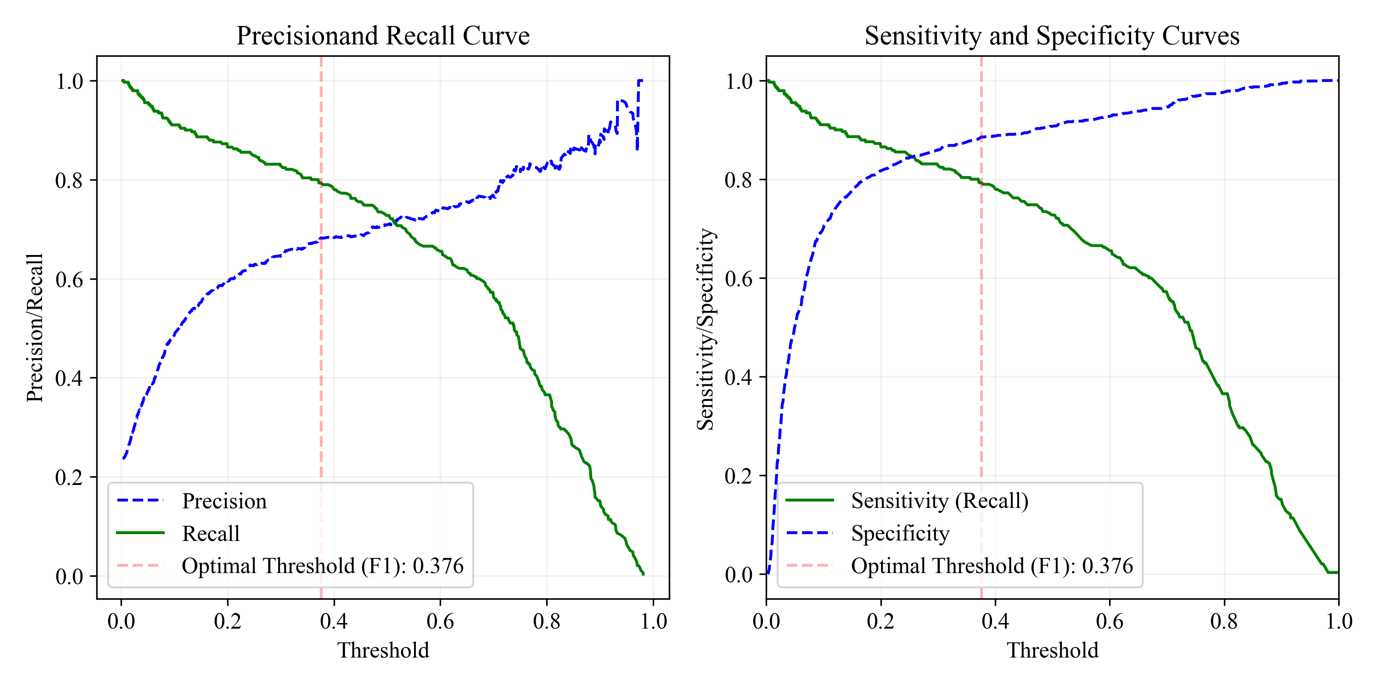


D.1. Bayesian Model (Male, Gonorrhoea)


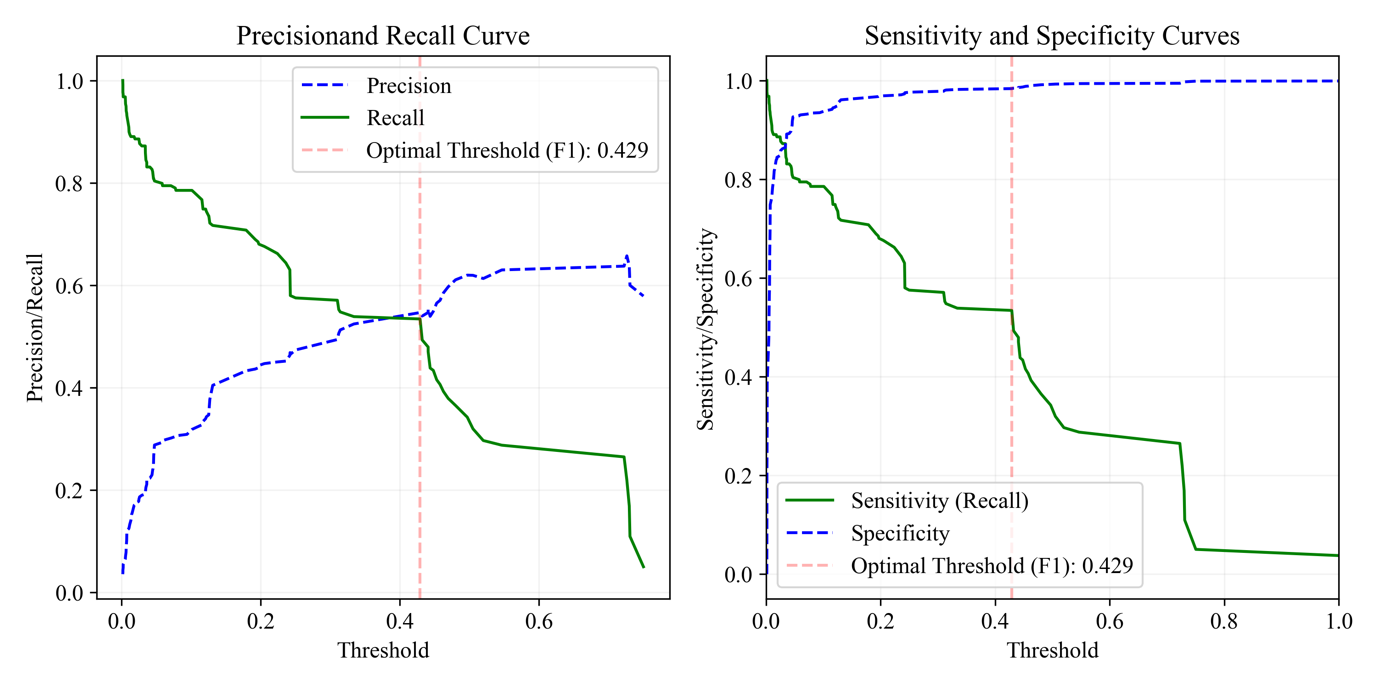


D.2. Machine Learning Model (Male, Gonorrhoea)


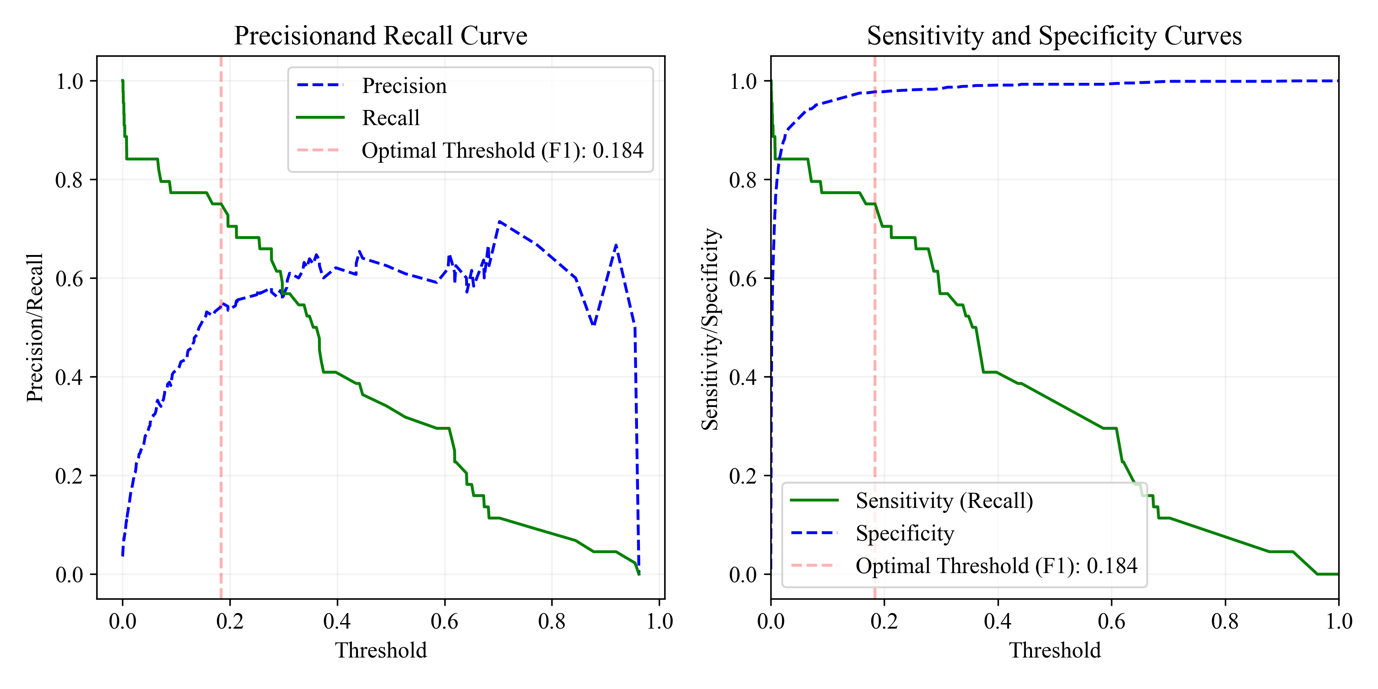


E.1. Bayesian Model (Male, Herpes)


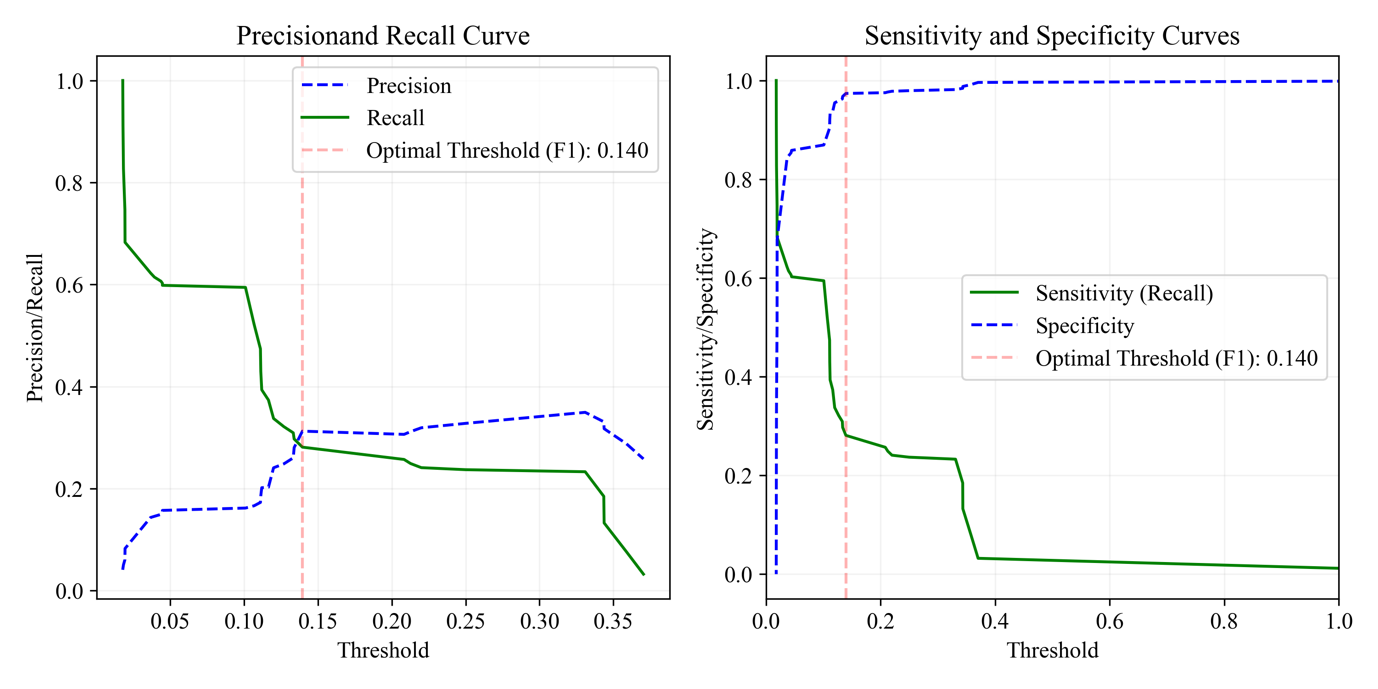


E.1. Machine Learning Model (Male, Herpes)


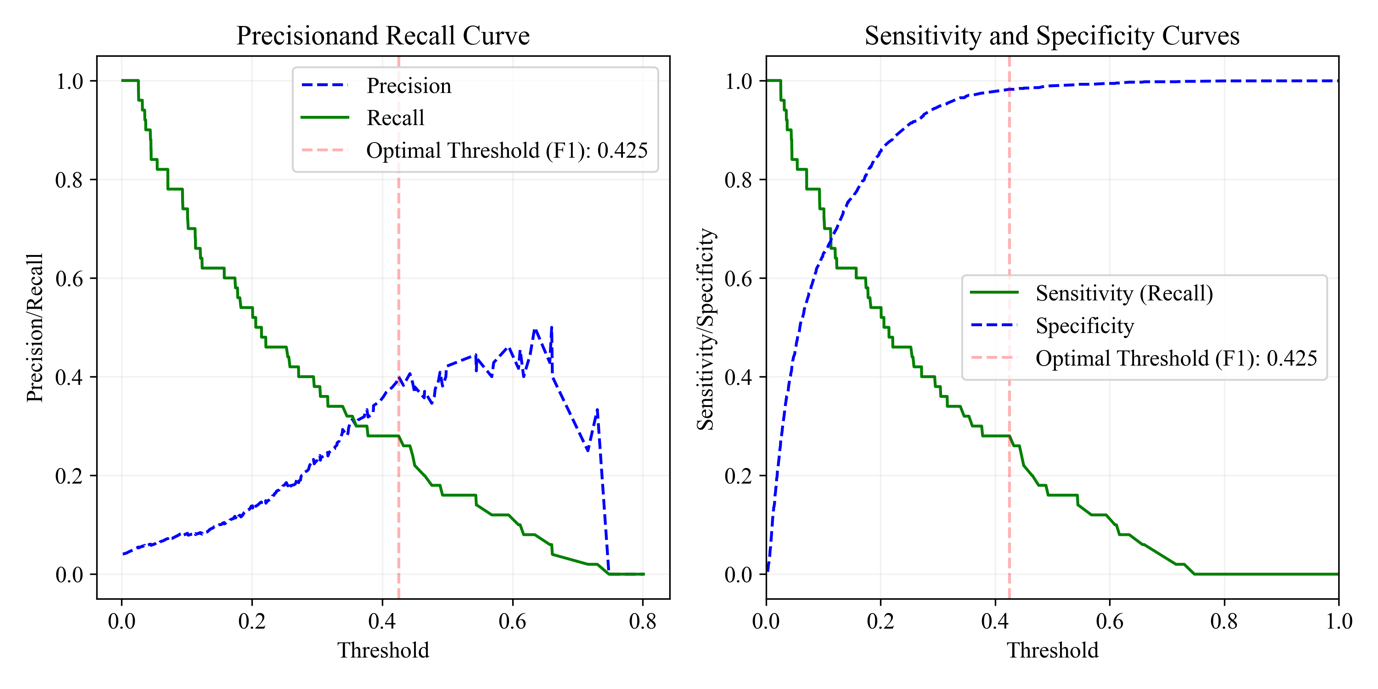


F.1. Bayesian Model (Male, Balanitis)


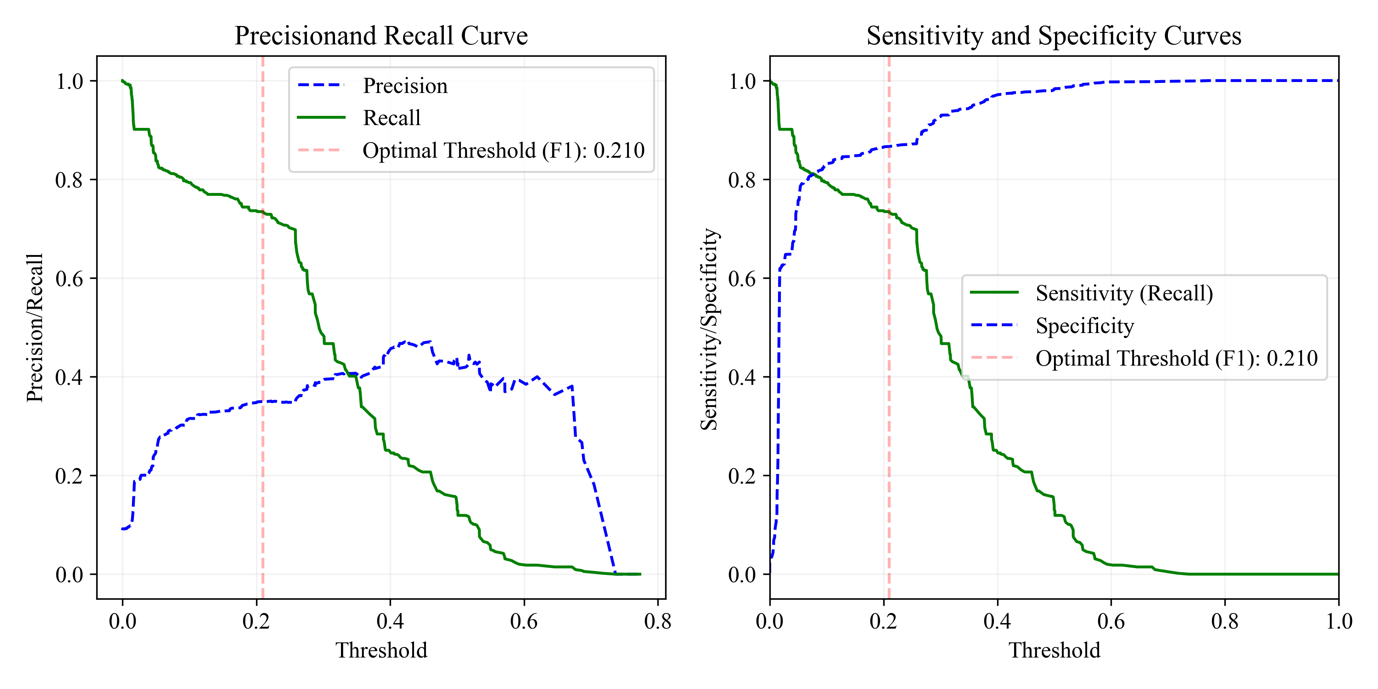


F.1. Machine Learning Model (Male, Balanitis)


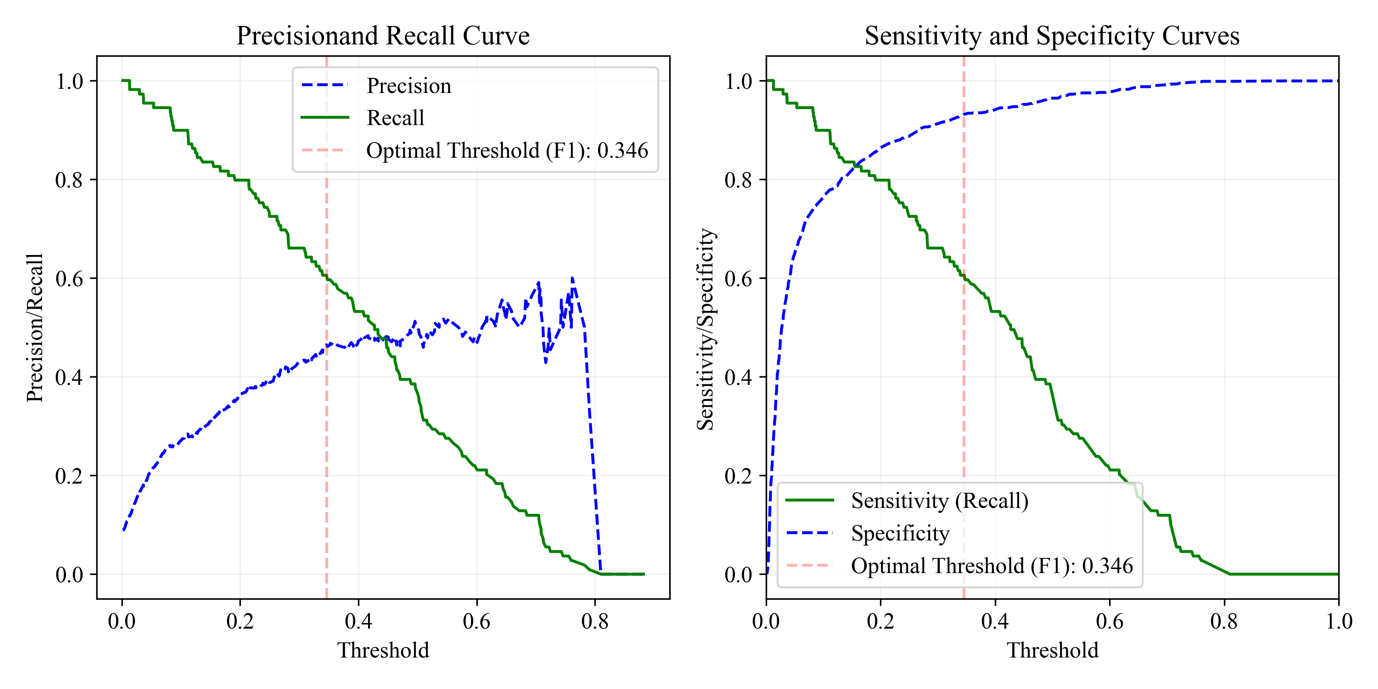


G.1. Bayesian Model (Male, Molluscum Contagiosum)


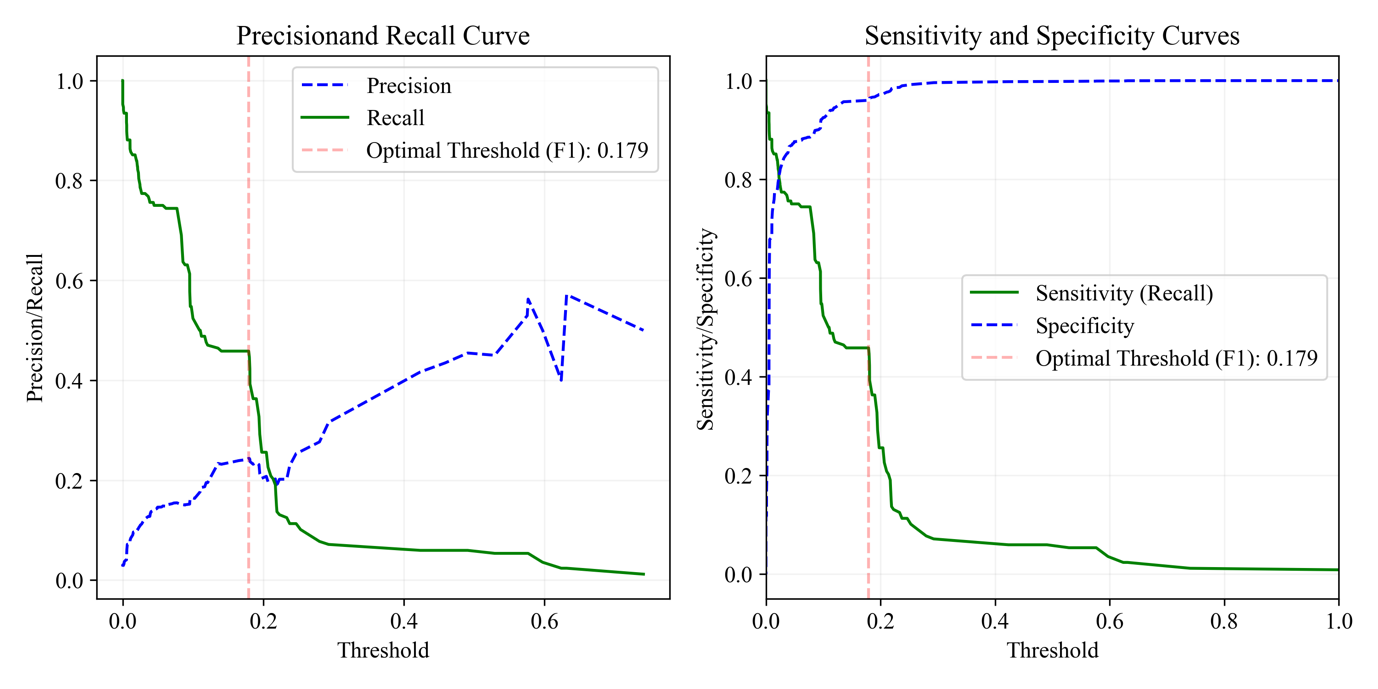


G.2. Machine Learning Model (Male, Molluscum Contagiosum)


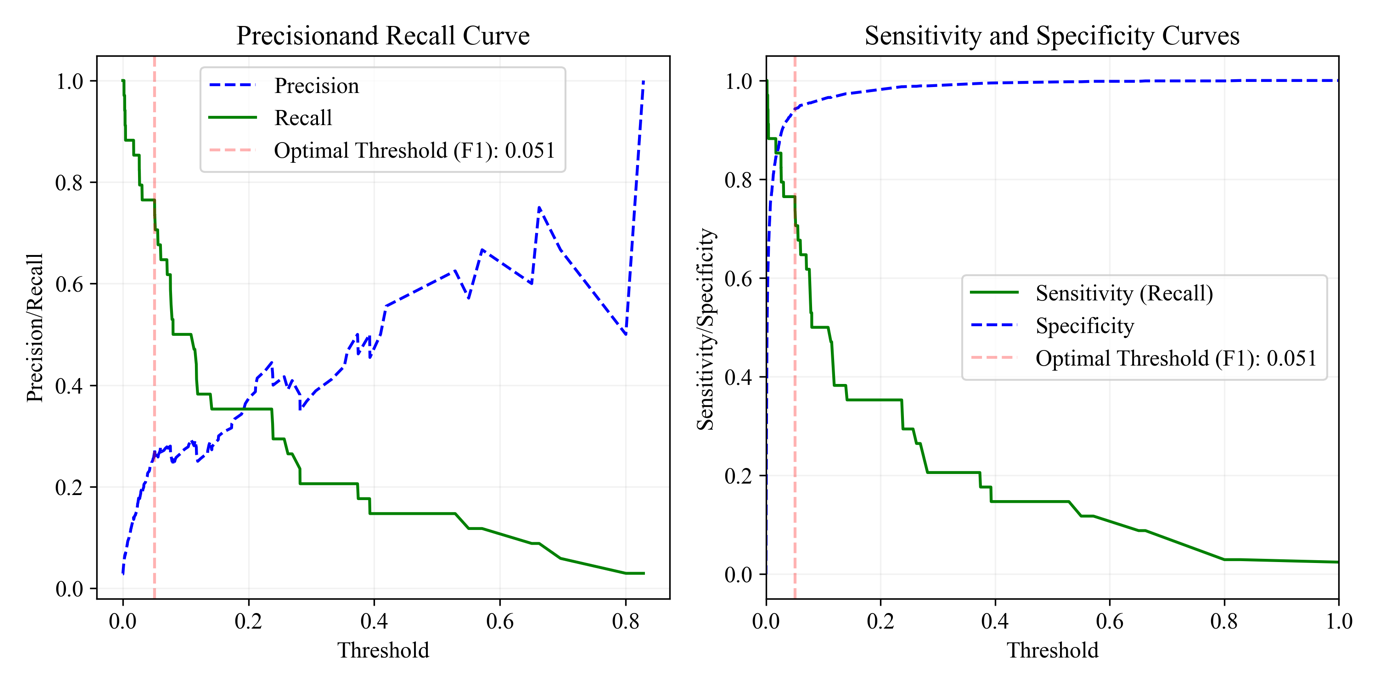


H.1. Bayesian Model (Female, PID)


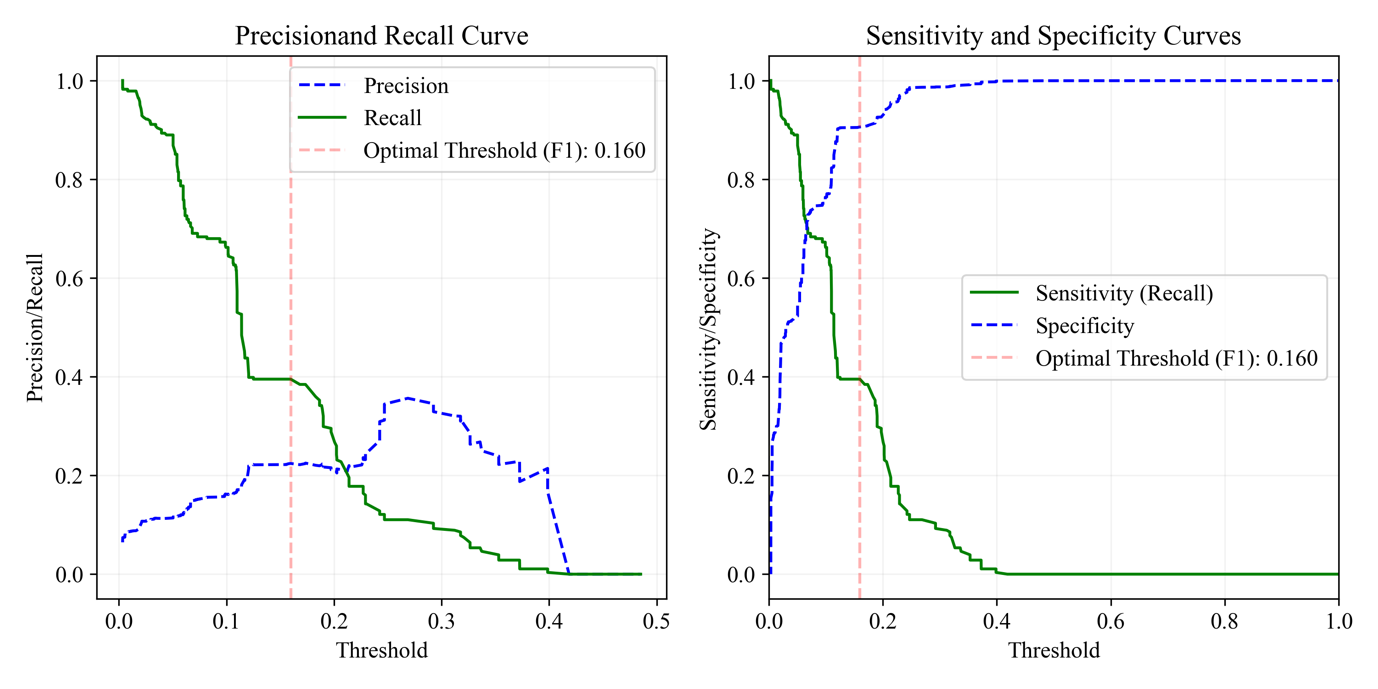


H.2. Machine Learning Model (Female, PID)


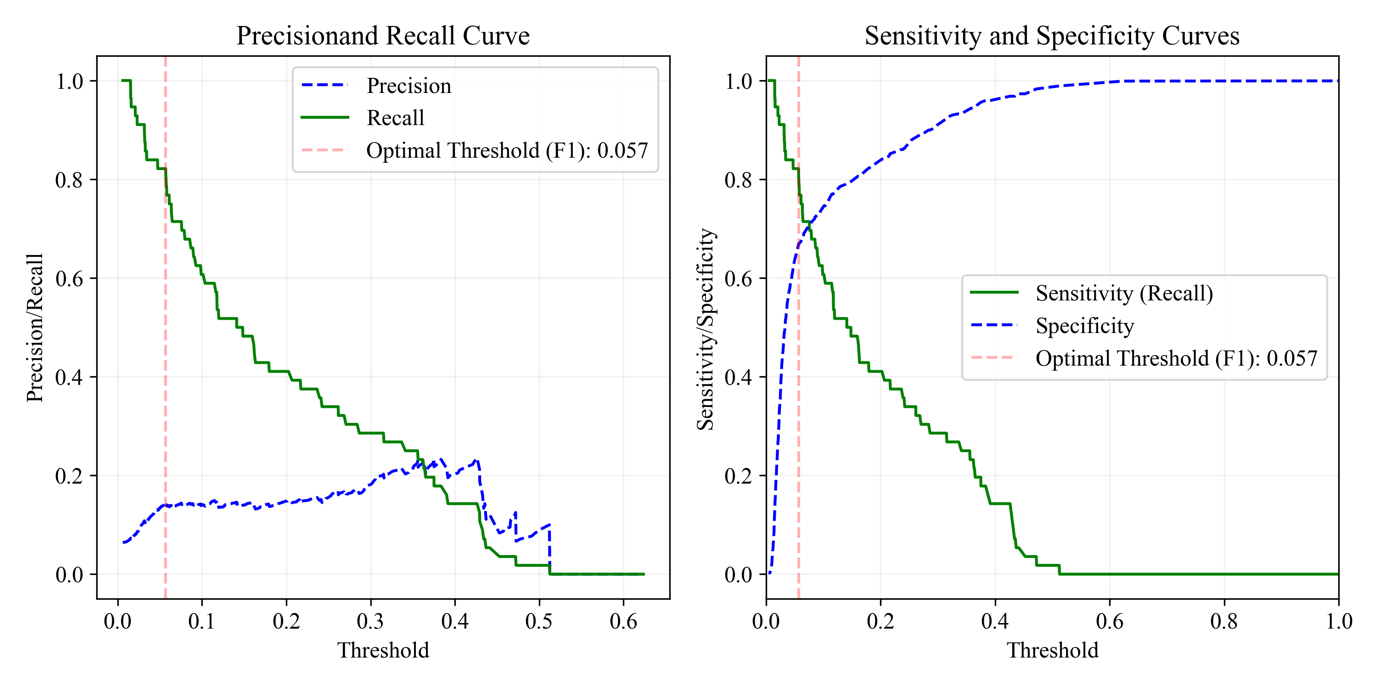


I.1. Bayesian Model (Female, Warts)


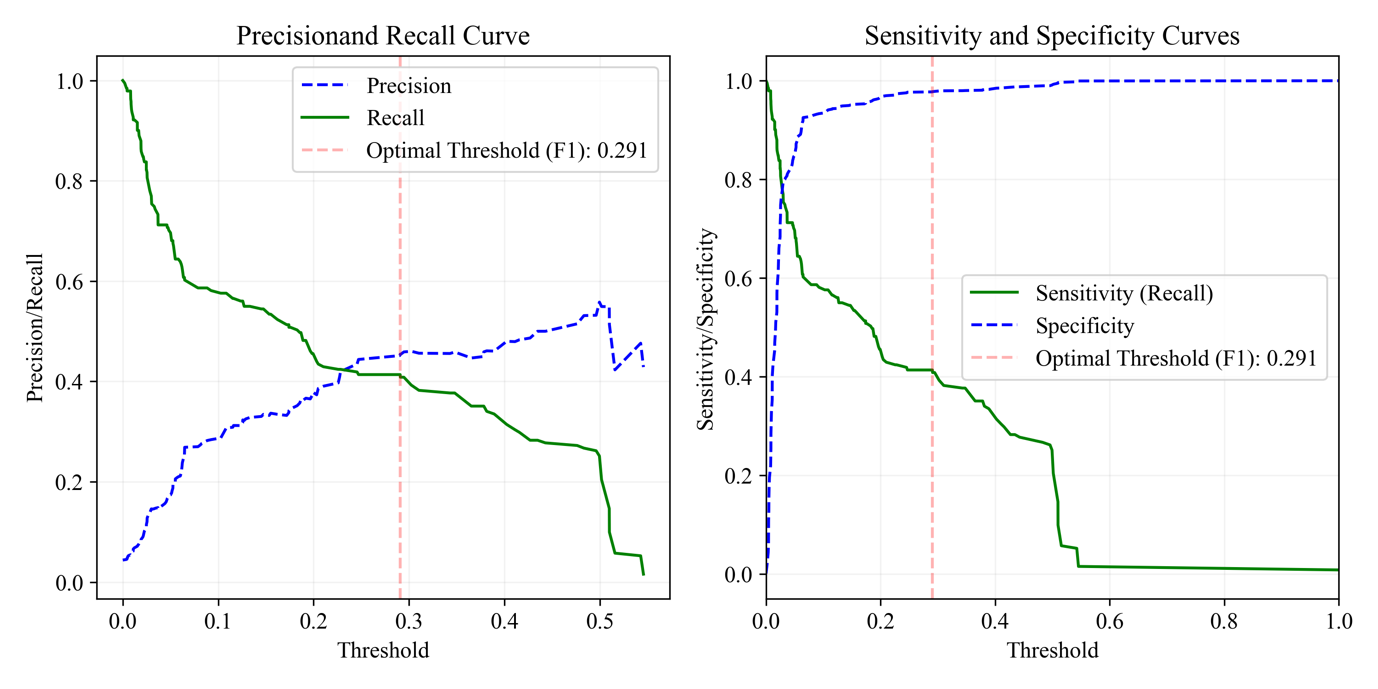


I.2. Machine Learning Model (Female, Warts)


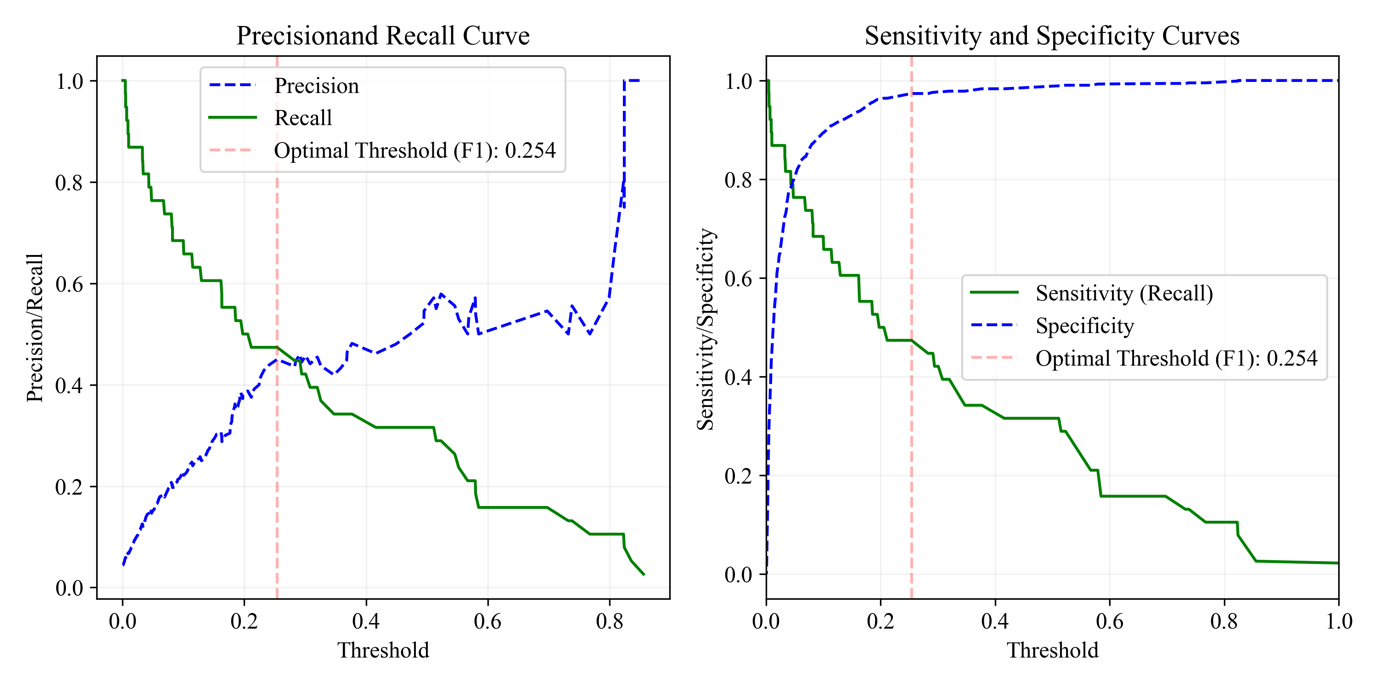


J.1. Bayesian Model (Female, Herpes)


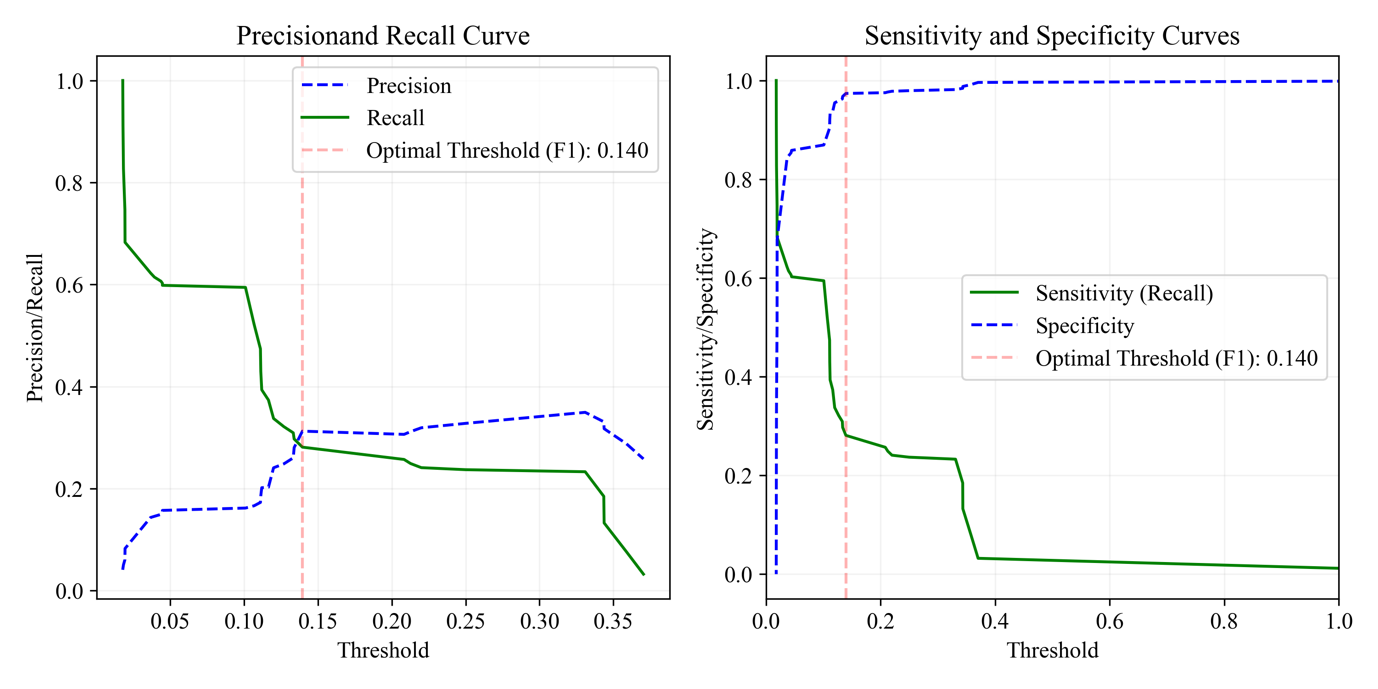


J.2. Machine Learning Model (Female, Herpes)


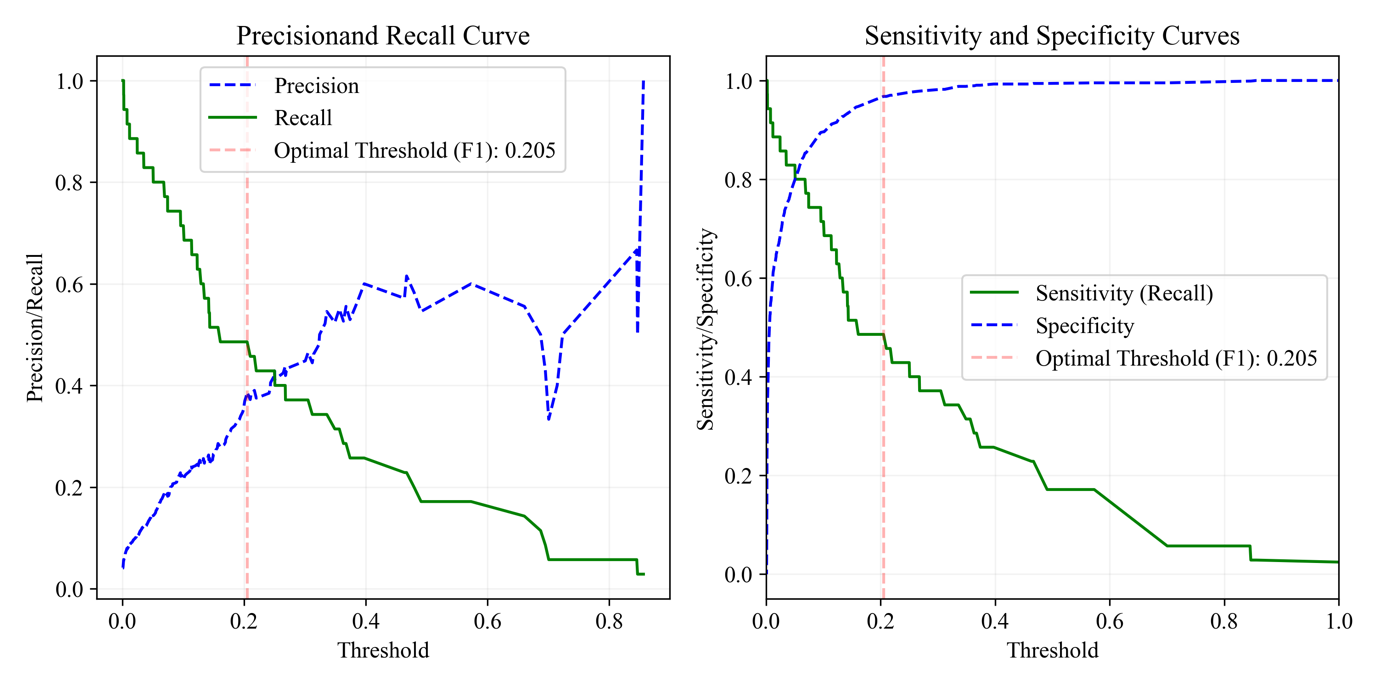


K.1. Bayesian Model (Female, Cystitis)


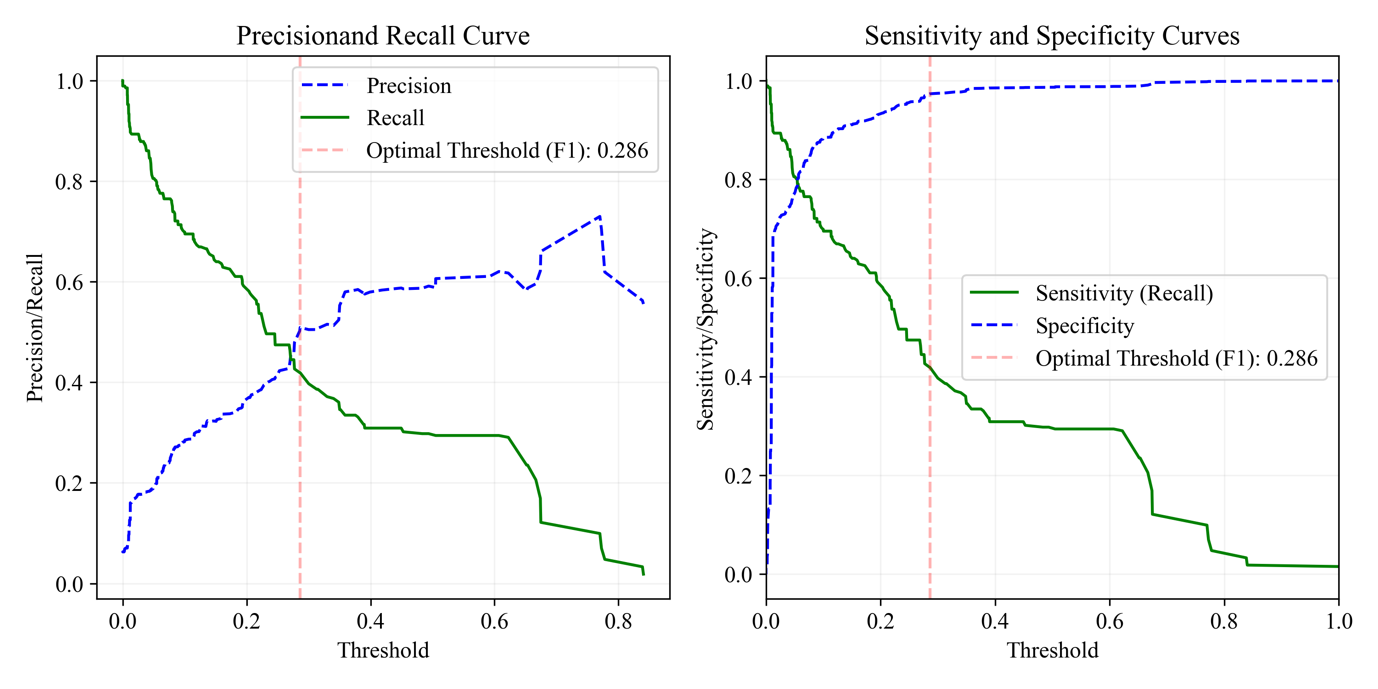


K.2. Machine Learning Model (Female, Cystitis)


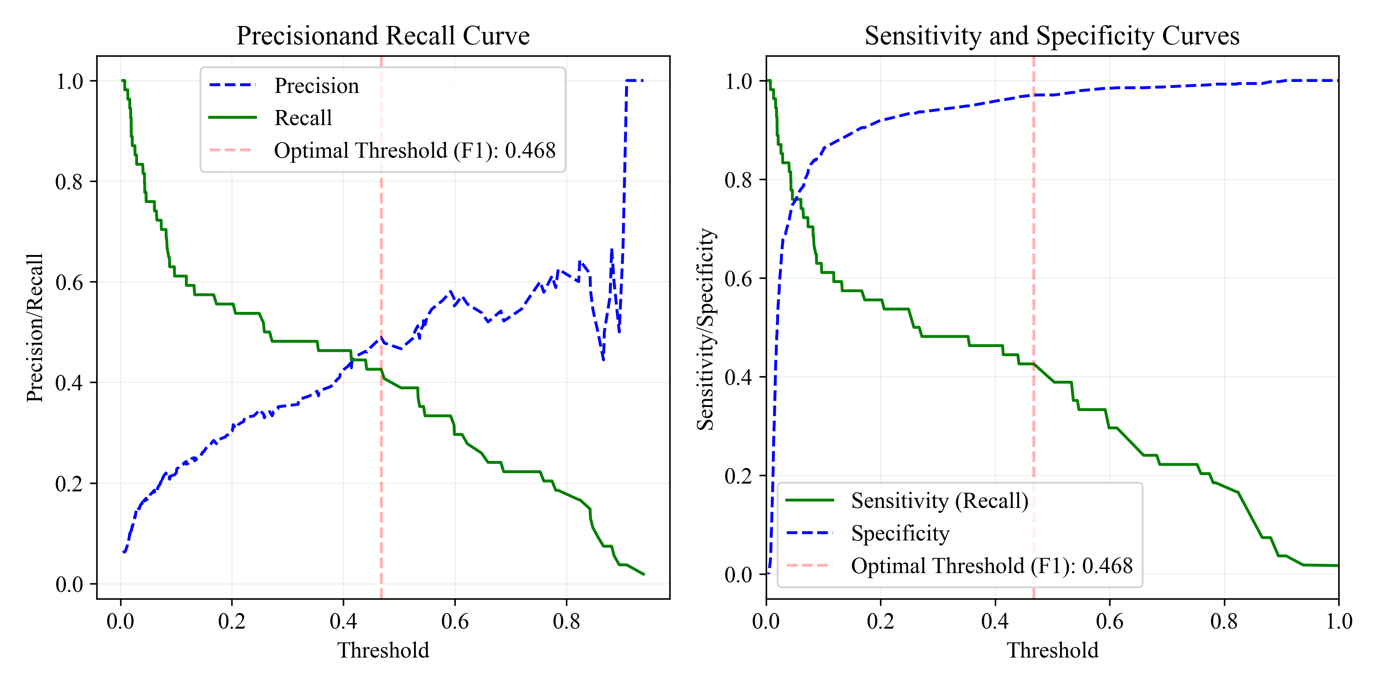


L.1. Bayesian Model (Female, Bacterial Vaginosis)


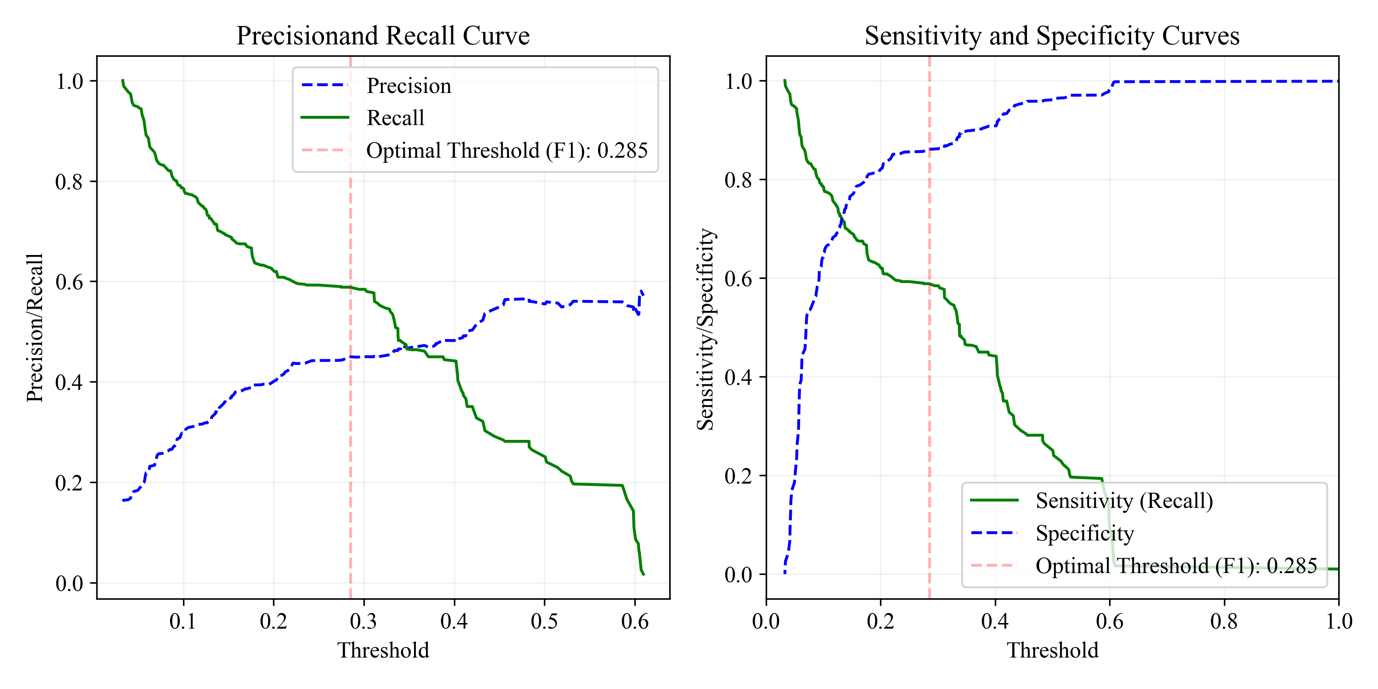


L.2. Machine Learning Model (Female, Bacterial Vaginosis)


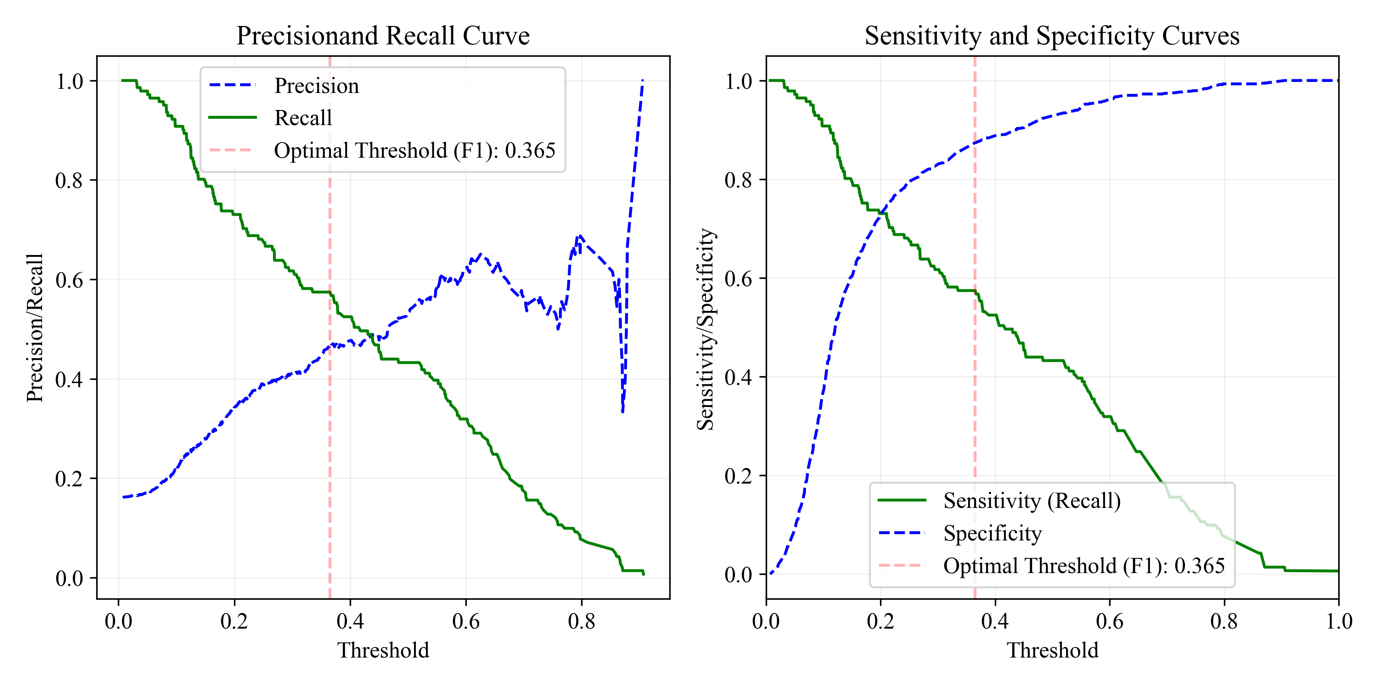


M.1. Bayesian Model (Female, Candidiasis)


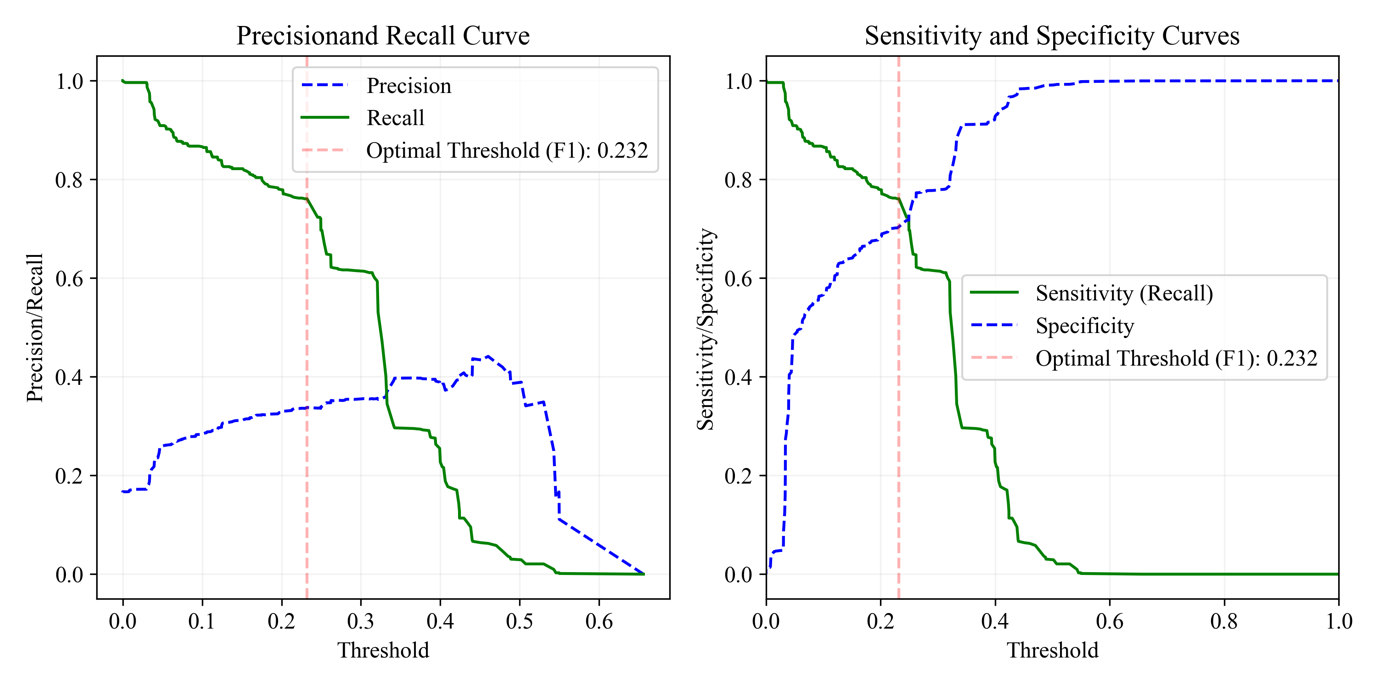


M.2. Machine Learning Model (Female, Candidiasis)


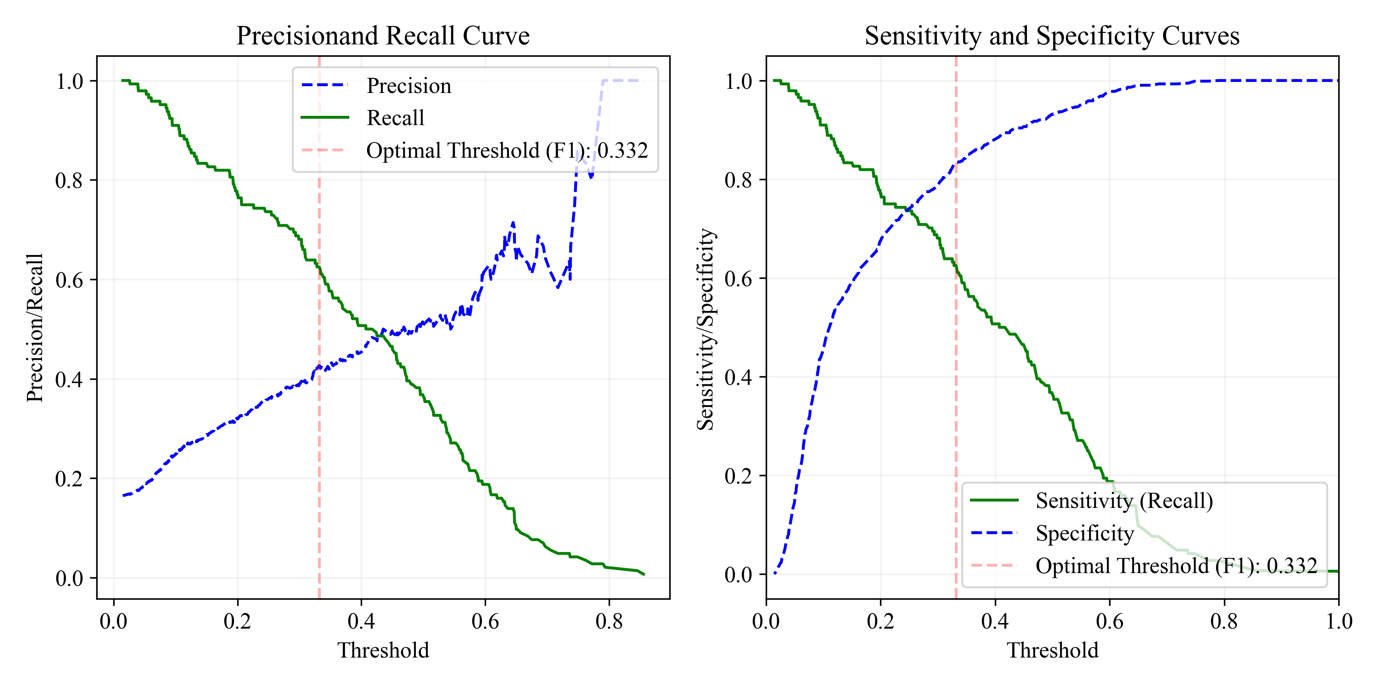


N.1. Bayesian Model (Female, Molluscum Contagiosum)


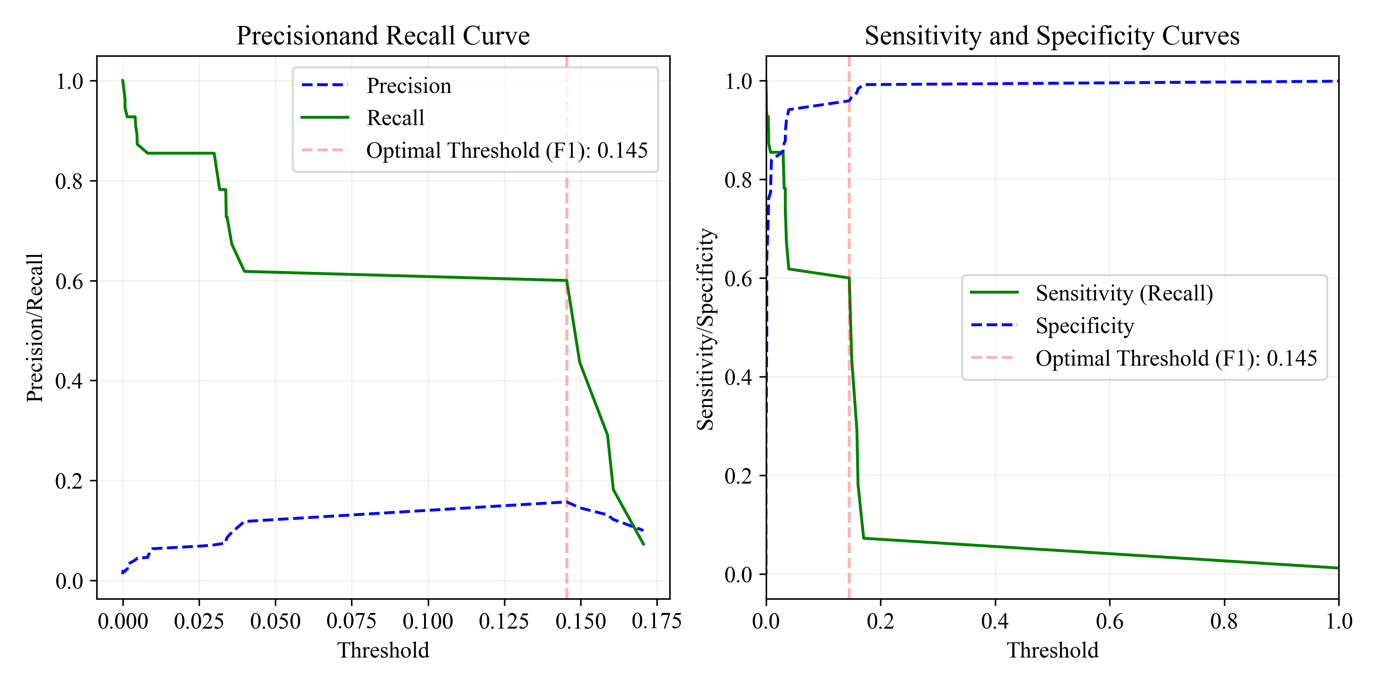


N.2. Machine Learning Model (Female, Molluscum Contagiosum)


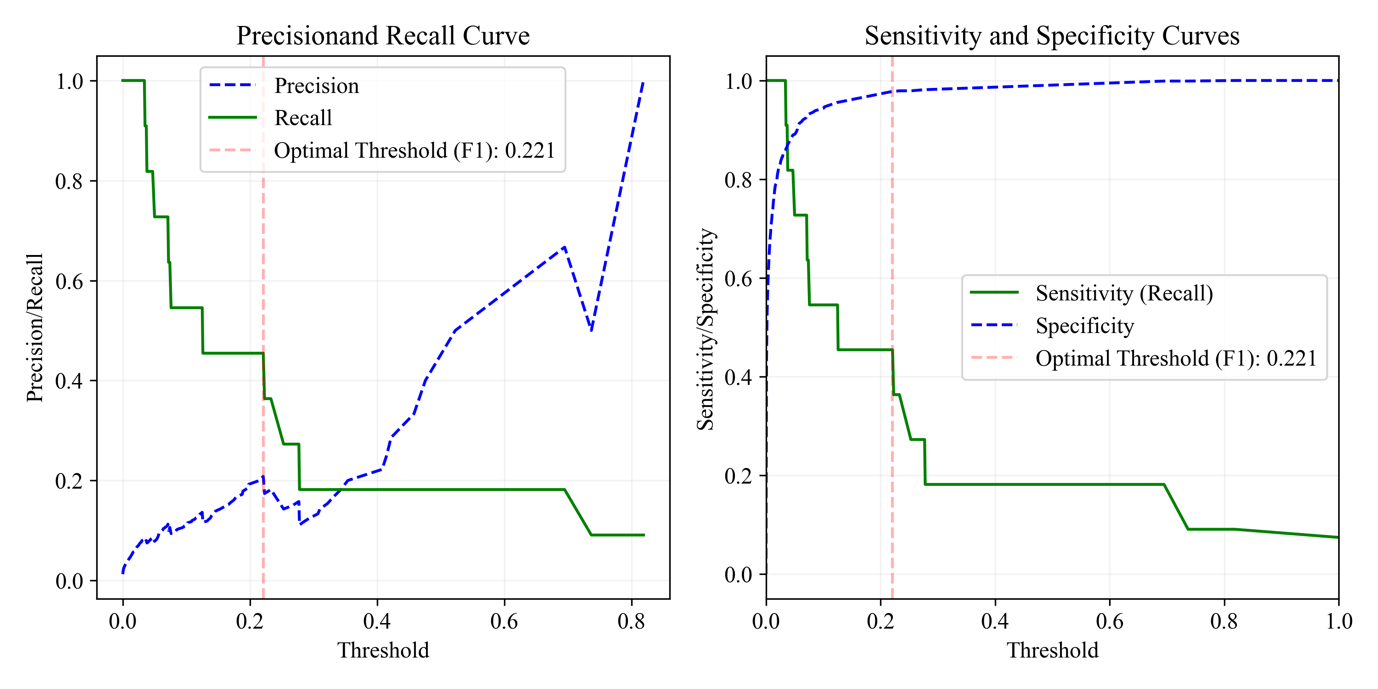

Supplement: Supplementary file 1 — Supplementary Material 1. Table 1. Demographic characteristics and presenting symptoms by diagnosis in male clients. Table 2. Demographic characteristics and presenting symptoms by diagnosis in female clients. Table 3. Performance comparison between machine learning and Bayesian Network models. Figure 1. Workflows for model development, evaluation and predictions. Figure 2. The AUC for the best performing machine learning models throughout validation, testing and external testing across different diseases. Table S1. Predictor variables and outcome variables. Table S2. Performance difference across validation, testing and external validation in male clients. Table S3. Performance difference across validation, testing and external validation in female clients. Figure S1. Performance comparison of machine learning models during training and Validation in male clients. Figure S2. Performance comparison of machine learning models during training and validation in female clients. Figure S3. Interpretation of machine learning models for conditions in males. Figure S4. Precision-Recall and Sensitivity-Specificity curves for Bayesian and Machine learning models for each disease condition. [file 12879_2024_10285_MOESM1_ESM.docx]
